# Population coding under the scale invariance of high-dimensional noise

**DOI:** 10.1126/sciadv.adz9632

**Published:** 2026-09-25

**Authors:** S. Amin Moosavi, Sai Sumedh R. Hindupur, Hideaki Shimazaki

**Affiliations:** ^1^ Department of Neurobiology, David Geffen School of Medicine, University of California, Los Angeles, Los Angeles, CA 90095, USA.; ^2^ School of Engineering and Applied Sciences, Harvard University, Cambridge, MA 02138, USA.; ^3^ Graduate School of Informatics, Kyoto University, Kyoto 606-8501, Japan.; ^4^ Center for Human Nature, Artificial Intelligence, and Neuroscience (CHAIN), Hokkaido University, Sapporo 060-0812, Japan.

## Abstract

High-dimensional scale-invariant neural activity is ubiquitous across brain regions and species, but its implications for information coding remain unclear. Here, we ask how stimulus information in the high-dimensional activity of mouse V1 scales with neuron number: Does it saturate due to noise correlations or increase without bound as subpopulations grow? Contrary to previous reports, we find that leading noise components that scale linearly with population size, and thus can limit information, are not sufficiently aligned with the signal to impose a bound. This conclusion follows from two scale-invariant power-law properties of neuronal responses in mouse V1: the noise eigenspectrum and alignment of noise components with the signal. We show that population subsampling links the observed power-law exponents to information boundedness and that information scaling depends on the full eigenspectrum rather than its leading modes. Last, we prove that, under subsampling, information-limiting correlations, if present, are differential correlations. Our findings clarify how information scales in high-dimensional neuronal activity under scale-invariant noise.

## INTRODUCTION

Neural activity is highly variable yet structured in a high-dimensional space (*1*–*3*), ubiquitously exhibiting quasi-universal, scale-invariant power-law spectra of noise components across various brain regions and species (*4*–*6*). Recent advances in theoretical and empirical studies suggest the potential mechanistic and functional origins of these scale-invariant properties (*4*–*8*). However, their impact on population coding remains poorly understood.

Classic works on neuronal activity in the monkey middle temporal area have revealed that the activity of single neurons is highly informative about the animal’s perceptual sensitivity in discriminating stimulus directions (*9*, *10*), suggesting that neurons encode stimuli in an overly redundant manner. In redundant coding, the information conveyed about a stimulus increases sublinearly as the size of the neural population grows, in contrast to the linear increase observed with independent coding (*10*, *11*). A hallmark of redundant coding is shared noise in neural activity across repeated trials, which obscures the signal quantified by the change in the average population activity in response to a change in stimulus (*12*–*14*). Recent high-throughput techniques for the simultaneous recording of hundreds or thousands of neurons have made it possible to visualize the sublinear increase in the conveyed information as a function of the observed number of neurons (*15*–*18*).

Redundant coding can be so strong that it may limit information: Coded information may saturate at its maximum value in the limit of a large number of neurons (*10*, *19*, *20*). Here, we assessed information scaling in large-scale recordings of neurons in mouse primary visual cortex (V1) (*21*) and tested the boundedness in substantially larger networks beyond observed populations. To this end, we used two scale-invariant properties revealed by the data: the scale-invariant noise spectrum and the scale-invariant relationship between the magnitude of noise components and their alignment with the signal. The approach transcends earlier large-scale studies (*15*–*18*, *21*) by replacing oversimplified assumptions about covariance scaling with the quasi-universal scaling observed in the data. Consequently, while these studies reported that information is bounded, we show that noise correlations, despite their detrimental effect on stimulus coding, do not limit the information. Therefore, neural populations have the capacity to encode stimulus information without a bound. These results demonstrate that the quasi-universal scaling of neural noise covariance provides a foundation for revealing the scaling and boundedness of population codes.

To establish the general principles underlying these findings, we developed a population coding theory based on the scaling properties of noise covariance under population subsampling. We derive a necessary and sufficient condition expressed in terms of covariance-eigenvalue scaling and the corresponding signal angular occupations, under which information remains bounded in the large-population limit. This condition highlights the critical need to consider the full spectrum of high-dimensional noise when predicting the scaling and boundedness of information coding in large neuronal populations. We further elucidate the constraints imposed by the covariance scaling on the power-law exponent of its eigenvalue spectrum and clarify how this exponent relates to the boundedness of information. Last, we characterize the incremental information gain by adding a neuron and relate the bounded regime to the specific noise structure known as differential correlations, which has been proposed to fundamentally limit information (*22*).

## RESULTS

### Scaling laws for subsampled neuronal populations

**Fig. 1.**
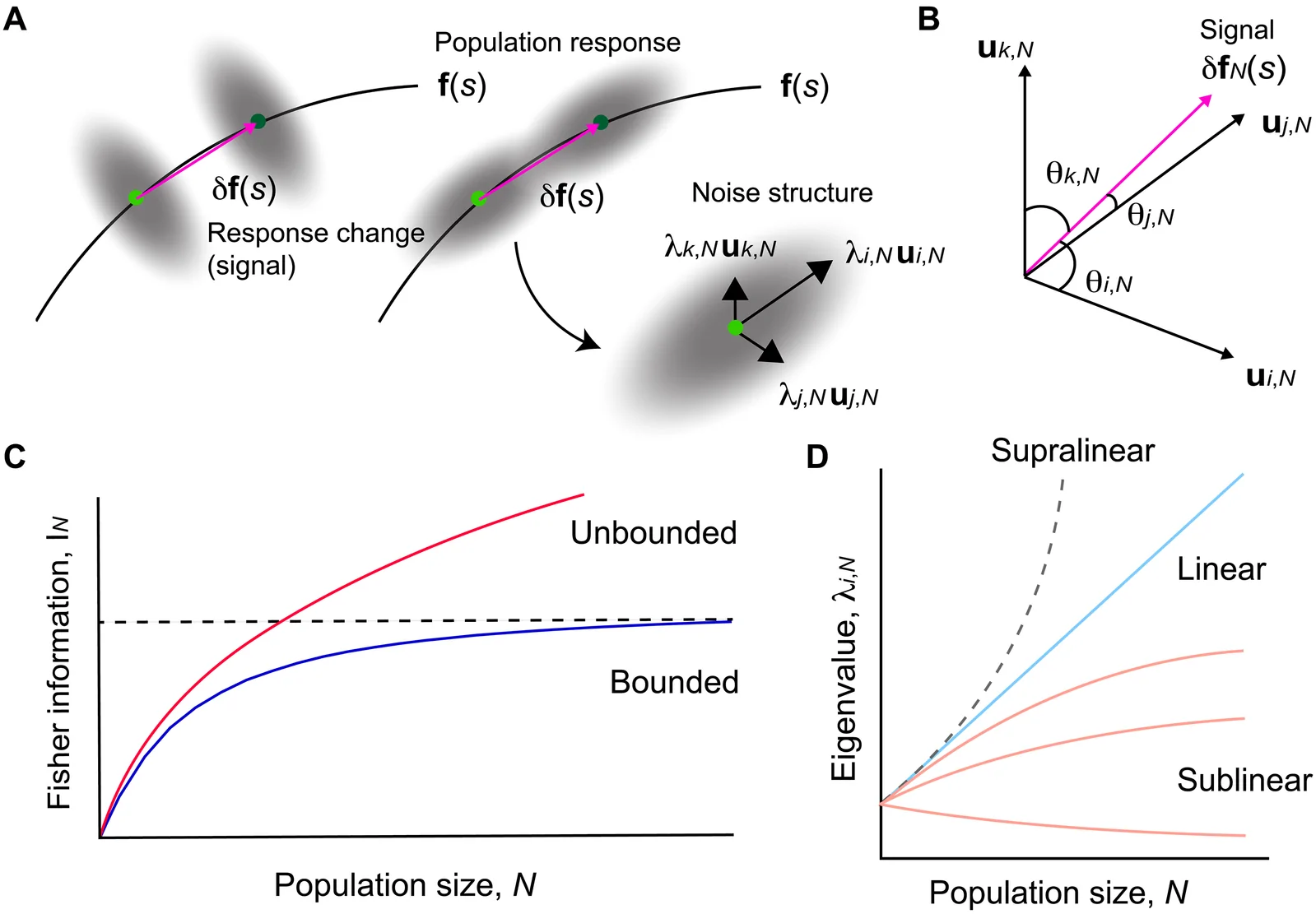
Schematic illustrating necessary and sufficient conditions for bounded and unbounded LFI. (**A**) A schematic of a subspace spanned by three neurons. The mean activity of neurons $f_{N}\left(s\right)$ encodes the stimulus *s* as first-order statistics (solid black line). In addition to the change in the mean responses (green dots) to the two close stimuli [$\delta f_{N}\left(s\right)$, magenta arrow, a finite-size analog of $f_{N}^{\;\prime }\left(s\right)$], the covariability (gray areas) affects stimulus coding beneficially (left) or detrimentally (right) when compared to circular independent noise. Noise covariance is quantified by eigenvectors $u_{i,N},u_{j,N}$, $u_{k,N}$, and their respective noise strengths $\lambda_{i,N},\lambda_{j,N},\lambda_{k,N}$. (**B**) A three-dimensional view of the orientation of $\delta f_{N}\left(s\right)$ with respect to the covariance eigenvectors. $\theta_{i,N}$ is the angle between $\delta f_{N}\left(s\right)$ and the eigenvector $u_{i,N}$. (**C**) The scaling of bounded (blue) and unbounded (red) LFI. (**D**) Supralinear, linear, and sublinear eigenvalues as a function of *N*. Supralinear eigenvalues are prohibited for subsampled populations.

The relationship between a signal and noise in the activity of *N* neurons evoked by multiple presentations of a stimulus (*s*) is represented in an *N*-dimensional space, where each dimension corresponds to the activity of a neuron (Fig. 1A). In this space, a vector $f_{N}\left(s\right)={\left[f_{1}\left(s\right),f_{2}\left(s\right),\ldots ,f_{N}\left(s\right)\right]}^{T}$ represents trial-averaged ensemble activity, and the signal denoted by $f_{N}^{\;\prime }\left(s\right)$ is the change in $f_{N}\left(s\right)$ caused by an infinitesimal change in the stimulus. Noise is the deviation from $f_{N}\left(s\right)$ during each stimulus presentation, quantified by a noise covariance matrix $\Sigma_{N}$ that is represented by its eigenvectors $u_{i,N}$ and eigenvalues $\lambda_{i,N}$ where i=1,…,N. These eigenvalues are indexed in descending order: $\lambda_{1,N}\geq \lambda_{2,N}\geq \ldots \geq \lambda_{N,N}$. The overlap between $f_{N}^{\;\prime }\left(s\right)$ and each $u_{i,N}$ is measured by the angle $\theta_{i,N}$ between the two vectors (Fig. 1B). Throughout this work, we focus on how these eigenvalues and angles scale as the population size *N* increases.

The coding capacity is quantitatively measured by the linear Fisher information (LFI) defined as $I_{N}=f_{N}^{\;\prime }{\left(s\right)}^{T}{\left(\Sigma_{N}\right)}^{-1}f_{N}^{\;\prime }\left(s\right)$ (*13*, *19*, *20*, *22*, *23*). We note that, although $I_{N}$ depends on the stimulus, in this study, we focus on its scaling with population size rather than variations with stimulus. Therefore, we constrained our analysis to small stimulus variations around a fixed value *s*. Using the noise components’ eigenvalues and the corresponding angles with $f_{N}^{\;\prime }\left(s\right)$, the LFI can equivalently be expressed as (text S1)

$$I_{N}=\sum_{i=1}^{N}\frac{{|f_{N}^{\;\prime }\left(s\right)|}^{2}}{\lambda_{i,N}}{\text{cos}}^{2}\theta_{i,N}$$ (1)

The LFI gives an upper bound on the information that linear decoders can read out. In other words, the optimal linear decoder—obtained by multiplying the inverse of the covariance matrix $\Sigma_{N}^{-1}$ with the signal vector $f_{N}^{\;\prime }\left(s\right)$—achieves a discrimination precision equal to the LFI (*13*).

The alignment of each noise component with the signal $f_{N}^{\;\prime }\left(s\right)$ is measured by ${\text{cos}}^{2}\theta_{i,N}$ termed angular occupation, which, because of the completeness of the orthonormal basis, satisfies

$$\sum_{i=1}^{N}{\text{cos}}^{2}\theta_{i,N}=1$$ (2)

Noise components that are not orthogonal to the signal $f_{N}^{\;\prime }\left(s\right)$ (${\text{cos}}^{2}\theta_{i,N}\neq 0$) contribute to the LFI. In correlated population responses, the alignment of $f_{N}^{\;\prime }\left(s\right)$ with larger components impairs the coding accuracy, and alignment with smaller ones improves the coding accuracy, compared to independent neurons.

With the above representation, we examined the scaling of the LFI with increasing subpopulation size *N* (Fig. 1C). Each subpopulation is constructed by randomly subsampling *N* neurons from a total of $N_{t}$ recorded neurons ($N\leq N_{t}$). Three factors contribute to the scaling of LFI: the signal magnitude, the noise components’ strength, and their alignments. The signal magnitude ${|f_{N}^{\;\prime }\left(s\right)|}^{2}$ scales linearly with *N* under the random subsampling

$${|f_{N}^{\;\prime }\left(s\right)|}^{2}∼N$$ (3)

This is because ${|f_{N}^{\;\prime }\left(s\right)|}^{2}$ is a summation of *N* nonnegative variables, which increases linearly with *N* if these variables have a finite, nonzero mean (text S1). Thus, whether the LFI is limited or not depends on how $\lambda_{i,N}$ scales against the linear ${|f_{N}^{\;\prime }\left(s\right)|}^{2}$ and how the angular occupation ${\text{cos}}^{2}\theta_{i,N}$ changes with *N*. Therefore, to assess the LFI scaling, from here onward, we categorized the covariance eigenvalues based on their behavior versus *N* as supralinear, linear, and sublinear eigenvalues (Fig. 1D; see text S2 for definition). Linear eigenvalues, if they exist, appear at early indices in the eigenspectrum in descending order, followed by the sublinear eigenvalues. These linear eigenvalues can potentially limit information growth if their associated noise directions sufficiently overlap with the signal direction.

The eigenvalues of the covariance matrix of subsampled populations are fundamentally constrained: The total variance must grow linearly with the population size *N*

$$\sum_{i=1}^{N}\lambda_{i,N}∼N$$ (4)

This follows from the fact that the sum of the covariance eigenvalues equals the total variance, which is itself the sum of the individual variances of all *N* neurons. Because each neuron’s variance is independent of the subsample size *N*, the total variance, like the signal magnitude, scales proportionally with *N*.

This introduces several crucial constraints on the eigenspectrum of the covariance matrix (text S2). First, the subsampled systems cannot have supralinear eigenvalues. Second, consider eigenvalue sequences that, for a fixed rank *i*, scale linearly with *N*. If their slopes remain above a fixed positive value, then their number must converge to a finite value; otherwise, their sum grows faster than *N*. The remaining eigenvalues, whose number then grows linearly with *N*, are sublinear and thus dominate the spectrum by number in the large-*N* limit. Third, if the slopes of the linear eigenvalues are allowed to shrink with the eigenvalue ranking such that their sum over rank is finite (i.e., summable), then the spectrum may contain a countably infinite family of fixed-rank linear eigenvalue sequences. In this case, linear total-variance scaling alone does not constrain the number of sublinear eigenvalues.

Having established the spectral scaling constraints that govern information boundedness under population subsampling, we then turned to experimental data to assess how the noise covariance spectrum scales in mouse V1 and how its alignment with $f_{N}^{\;\prime }\left(s\right)$ influences information boundedness.

### Unbounded information scaling in mouse V1 neurons

**Fig. 2.**
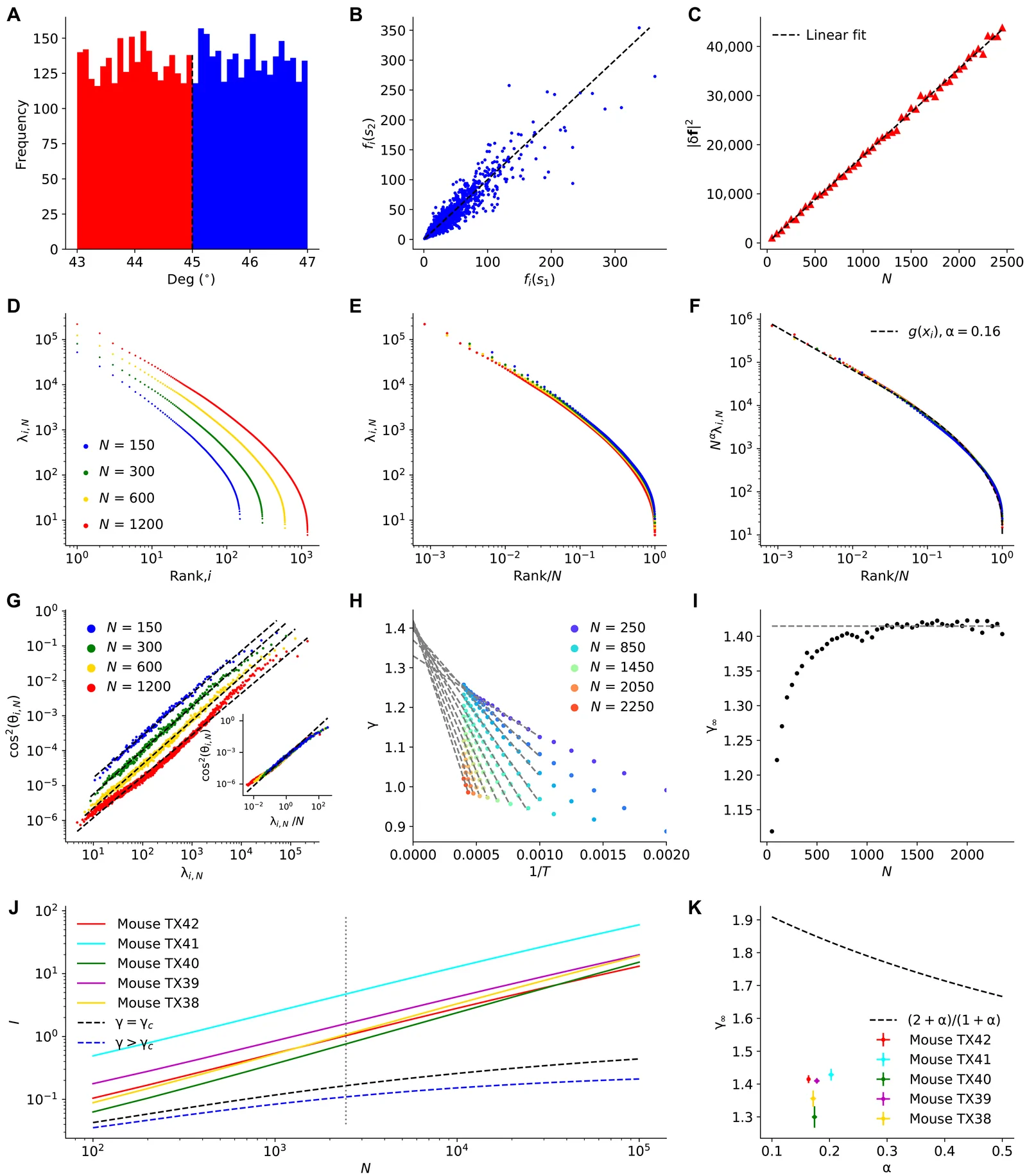
Scaling analysis of mouse V1 neurons. (**A**) Histogram of grating stimulus orientations presented to mouse TX42. Stimuli with orientations from 43° to 45°, including 45° (red), are categorized as stimulus 1 and those above 45° and up to 47° (blue) as stimulus 2. (**B**) Mean responses $f_{i}\left(s\right)$ of neurons (i=1,…,18,982) to stimuli 1 ($s=s_{1}$) versus 2 ($s=s_{2}$) for TX42. $f_{i}\left(s\right)$ is the mean response over trials in each stimulus category. Dashed line, identity. (**C**) Mean response changes ${|\delta f_{N}|}^{2}$ versus subpopulation size *N*, where $\delta f_{N}=f_{N}\left(s_{2}\right)-f_{N}\left(s_{1}\right)$, for TX42. Values are averaged over 200 randomly chosen subpopulations. Dashed line, linear fit. (**D**) Noise eigenspectra $\lambda_{i,N}$ for different subpopulation sizes *N* in TX42. (**E**) Eigenspectra versus the normalized rank, $x_{i}=i/N$. (**F**) Rescaled eigenspectra $N^{\alpha }\lambda_{i,N}$, showing collapse onto the fitted scaling function $g\left(x_{i}\right)$ (dashed line; Eq. 6). For TX42, α=0.164±0.002; fitted parameters of $g\left(x_{i}\right)$ are a=2.752, exp(b)=9.611, c=2.298, and ν=0.254. (**G**) ${\text{cos}}^{2}\theta_{i,N}$ versus $\lambda_{i,N}$ for different *N* in TX42 using all trials (2500 per stimulus). Dashed lines, linear fits. Inset: ${\text{cos}}^{2}\theta_{i,N}$ versus $\lambda_{i,N}/N$. (**H**) Fitted exponent γ in Eq. 7 versus 1/T for different *N*, where *T* is the number of trials. Solid lines, linear fits; intercepts estimate γ at an infinite trial number. (**I**) Intercept $\gamma_{\infty }$ versus *N*. Mean $\gamma_{\infty }$ for *N* = 1000 to 2500 (dashed line) was 1.415±0.006. (**J**) Inferred LFI versus *N* for TX42 and four other mice (solid lines). The LFI was computed from Eqs. 5 and 7, using the parameters fitted to each mouse. Dashed lines show theoretical LFI resulting in critical (black, $\gamma =\gamma_{c}$) and bounded information (blue, $\gamma >\gamma_{c}$) for TX42. Vertical dashed line, neuron number used for parameter estimation. (**K**) Estimated $\gamma_{\infty }$ versus α for all mice. Dashed line, critical boundary (Eq. 8); error bars, 2 SD.

We analyzed stimulus-evoked responses of a population of mouse V1 neurons recorded by two-photon imaging (see Materials and Methods). The experiments were designed to examine the population’s ability to detect small changes in orientations of grating stimuli (*21*). In each trial, the stimulus orientation was randomly drawn from a uniform distribution in the range [43°, 47°]. Following Stringer *et al.* (*21*), we grouped the trials into two adjacent angular ranges to quantify local discriminability between the two groups (Fig. 2A). Although the presented stimuli are finite and discrete, the question here is how local discriminability scales with subpopulation size. The dataset includes 17,631 to 21,469 neurons recorded from five mice with ∼1000 repeated trials per degree while animals passively viewed stimuli.

These neurons exhibited linearly scaling ${|f_{N}^{\;\prime }\left(s\right)|}^{2}$ (Fig. 2, B and C), as expected. Figure 2D shows that the eigenspectrum of the noise covariance matrix scales systematically with subpopulation size. When the eigenspectrum is represented using the normalized index $x_{i}=i/N$ (Fig. 2E), the eigenspectrum is in agreement with the following scale invariance

$$\lambda_{i,N}=N^{-\alpha }g\left(x_{i}\right)$$ (5)

where α>0. Accordingly, plotting $N^{\alpha }\lambda_{i,N}$ against $x_{i}$ produces a collapse of data onto a scale-invariant function $g\left(x_{i}\right)$ that is independent of subpopulation size (Fig. 2F). For mouse TX42, the exponent obtained by finite-size scaling collapse of the data (see Materials and Methods) was α=0.164. This exponent appears quasi-universal among all the mice in the experiment (α=0.18±0.01, mean ± SD across five mice; figs. S1 to S5).

By carefully constructing an ansatz consistent with the subsampling constraints and the observed scaling collapse, we found the following form for the scale-invariant function

$$g\left(x_{i}\right)=x_{i}^{x_{i}-1-\alpha }\text{exp}\left[b\left(1-x_{i}\right)+a{\left(-x_{i}\text{log}x_{i}\right)}^{\nu }+c\right]$$ (6)

The exponential function accounts for the cutoff that occurs at $x_{i}=1$ by definition, whereas the power-law part explains the behavior of larger eigenvalues in the $x_{i}\to 0$ limit. In particular, considering Eq. 5, the power law is defined such that linear eigenvalues are permitted as $x_{i}\to 0$ when α>0; and the total variance, i.e., the sum of all eigenvalues, scales linearly with *N*. While the functional form of $g\left(x_{i}\right)$ is independent of *N*, its domain $x_{i}\in \left[1/N,2/N,\ldots ,1\right]$ depends on *N*. Unlike α, the remaining fitted parameters of $g\left(x_{i}\right)$ varied across mice.

The equations above specify the composition of the eigenspectrum in the large-*N* limit. At small normalized rank ($x_{i}\ll 1$), the scaling function follows a power law, $g\left(x_{i}\right)∼x_{i}^{-\left(1+\alpha \right)}$. This power law describes scaling with eigenvalue rank, whereas Eq. 5 describes scaling with subpopulation size *N*. When α>0, the eigenvalue at any fixed rank *i* grows linearly with *N* because $\lambda_{i,N}∼i^{-\left(1+\alpha \right)}N$. Namely, for every fixed positive integer $N_{c}$, the first $N_{c}$ eigenvalue sequences increase linearly with *N*. Hence, in the large-*N* limit, the spectrum contains a countably infinite family of fixed-rank linear eigenvalue sequences. The contribution of this fixed-rank linear family is compatible with the linear total variance constraint (Eq. 4) when the linear coefficients $i^{-\left(1+\alpha \right)}$ are summable, i.e., α>0. Although this family is countably infinite, eigenvalues at ranks proportional to *N* scale as $N^{-\alpha }$ and are therefore sublinear. Thus, sublinear eigenvalues dominate the spectrum by number in the large-*N* limit.

The alignment of the noise eigenvectors linked to linear or sublinear eigenvalues with $f_{N}^{\;\prime }\left(s\right)$ determines whether the LFI is bounded. The angular occupations ${\text{cos}}^{2}\theta_{i,N}$ and eigenvalues $\lambda_{i,N}$ show linear dependency on a logarithmic scale (Fig. 2G), indicating another scale-invariant relation

$${\text{cos}}^{2}\theta_{i,N}=\frac{\lambda_{i,N}^{\gamma }}{\Sigma_{i^{\prime }=1}^{N}\lambda_{i^{\prime },N}^{\gamma }}$$ (7)

The eigenvectors tied to the larger eigenvalues occupy a large portion of the angular occupations, indicating that the linear eigenvalues align with $f_{N}^{\;\prime }\left(s\right)$ and potentially limit the information. By contrast, the eigenvectors associated with the smaller eigenvalues exhibit smaller angular occupations [nearly orthogonal to $f_{N}^{\;\prime }\left(s\right)$]. Yet, because of the sublinearity, their contributions to the coded information can be substantial. From Eqs. 1, 4, and 7, one can see $I_{N}∼N$ for γ=1 and $I_{N}\to I_{\infty }$ for γ=2, where $I_{\infty }={\text{lim}}_{N\to \infty }I_{N}<\infty$ denotes the limiting LFI (Materials and Methods and text S3). Therefore, a critical exponent $\gamma =\gamma_{c}$ exists between one and two, where the information changes behavior from unbounded to bounded. We find that this critical exponent is given by

$$\gamma_{c}=\frac{2+\alpha }{1+\alpha }$$ (8)

Specifically, in the large-*N* limit, the LFI scales as

$$I_{N}∼\left\{\begin{array}{ll} I_{\infty } & \gamma >\gamma_{c}\\ \text{log}N & \gamma =\gamma_{c}\\ N^{2-\alpha \left(\gamma -1\right)-\gamma } & 1\leq \gamma <\gamma_{c} \end{array}\right.$$ (9)

For α=0.164 in mouse TX42, the critical exponent is $\gamma_{c}=1.859$. The exponent γ estimated by extrapolating to the infinite-trial limit, denoted $\gamma_{\infty }$, is $\gamma_{\infty }=1.415\pm 0.006$ (Fig. 2, H and I). As $\gamma_{\infty }$ is smaller than $\gamma_{c}$, the result indicates that the LFI of the neurons in this dataset increases without bound. In this regime, the LFI asymptotically scales as a power law with the exponent 2−α(γ−1)−γ=0.517, which is a sublinear but unbounded function of *N*. Such sublinear, unbounded behavior was consistently observed across all five mice (Fig. 2, J and K; see figs. S1 to S5 for individual results). Accordingly, we conclude that the mouse V1 neurons’ correlated activity causes redundancy, yet it is insufficient to limit information in the large-*N* limit. Therefore, the only constraints on the information capacity are the limited stimulus information available in the retina (*24*) and the total number of neurons in V1 and related areas, which are not imposed in this theory.

We performed the analyses above using the raw sample eigenvalues and eigenvectors. However, because finite-sample estimation introduces biases into both the eigenspectrum and the angular occupations, we also developed a finite-sample bias correction method. The procedure consists of three steps: iterative shrinkage of the eigenspectrum, an iterative Wishart-surrogate correction for angular occupations, and an analytical perturbative correction for residual bias in the estimated signal vector. Together, these steps yielded reliable bias-corrected estimates of both the eigenspectrum and the angular occupations (fig. S6). See Materials and Methods for details of the algorithms. We applied the method to empirical data (fig. S7) and verified that the main conclusions were robust to these corrections: After bias correction, the exponents were α=0.10±0.02, γ=1.26±0.20, and $\gamma_{c}=1.91\pm 0.01$ (mean ± SD across five mice), with $\gamma <\gamma_{c}$ in every animal.

### Linear total variance of subsampled populations constrains eigenvalue scaling, power-law exponent, and boundedness of the LFI

Here, we summarize the general consequences for scale-invariant eigenspectra (Eq. 5) imposed by the linear scaling of the total variance required for subsampled populations (Eq. 4). Since there are no supralinear eigenvalues for subsampled populations, the LFI (Eq. 1) can be decomposed into the contributions by components with linear and sublinear eigenvalues

$$I_{N}=\sum_{i\in L}\frac{{|f_{N}^{\;\prime }\left(s\right)|}^{2}{\text{cos}}^{2}\theta_{i,N}}{\lambda_{i,N}}+\sum_{i\in S_{b}}\frac{{|f_{N}^{\;\prime }\left(s\right)|}^{2}{\text{cos}}^{2}\theta_{i,N}}{\lambda_{i,N}}$$ (10)

where *L* and $S_{b}$ are sets of linear and sublinear eigenvalues, and ${|f_{N}^{\;\prime }\left(s\right)|}^{2}∼N$.

Because the two contributions are nonnegative, $I_{N}$ is bounded if and only if both are bounded. We first consider the case in which the fixed-rank linear family is finite. At large *N*, the number of elements in set *L* approaches a constant integer c≥0; consequently, the number of elements in set $S_{b}$ linearly increases with *N*: $\left|L\right|\to c$ and $\left|S_{b}\right|\to \infty$ as N→∞. The first term remains bounded because L is finite and, for each i∈L, $N/\lambda_{i,N}\to 1/b_{i}<\infty$. Therefore, in this first scenario, the boundedness of the second summation is necessary and sufficient for bounded information: LFI boundedness is determined by the sublinear components, not by the linear eigenvalues. Defining the occupation of ${\text{cos}}^{2}\theta_{i,N}$ from sublinear eigenvectors by $W_{S_{b}}\left(N\right)=\sum_{j\in S_{b}}{\text{cos}}^{2}\theta_{j,N}$, the boundedness condition can be written as (text S4)

$$W_{S_{b}}\left(N\right)\langle \frac{N}{\lambda_{i,N}}\rangle \leq C$$ (11)

for all sufficiently large *N*, where C>0 is a finite constant independent of *N*. For $W_{S_{b}}\left(N\right)>0$, we define $\langle X\rangle =\sum_{i\in S_{b}}\pi_{i}X$, where $\pi_{i}={\text{cos}}^{2}\theta_{i,N}/W_{S_{b}}\left(N\right)$; if $W_{S_{b}}\left(N\right)=0$, the sublinear contribution vanishes. Equivalently, the necessary and sufficient condition for bounded LFI is that $W_{S_{b}}\left(N\right)$ decreases to zero at a rate equal to or faster than ${\langle N/\lambda_{i,N}\rangle }^{-1}$, which sets the critical rate of convergence. This critical rate is determined by the scaling of the sublinear eigenvalues together with their associated angular occupations. This condition can equivalently be expressed in terms of the angular occupation of the linear eigenvectors, $W_{L}\left(N\right)$, using $W_{L}\left(N\right)=1-W_{S_{b}}\left(N\right)$. Specifically, $1-W_{L}\left(N\right)$ must decrease to zero at least as fast as the critical rate set by the sublinear components. This shows that alignment of the signal with the linear-eigenvalue subspace ($W_{L}\left(N\right)\to 1$) is necessary but not sufficient for bounded LFI.

Next, we consider the case in which the family of fixed-rank linear eigenvalue sequences is countably infinite and their linear slopes ($b_{i}$), defined by $\lambda_{i,N}∼b_{i}N$, form a summable sequence, $\sum_{i=1}^{\infty }b_{i}<\infty$. In this case, in addition to the boundedness of the sublinear-eigenvalue contribution, the angular occupations of the fixed-rank linear eigenvalue sequences must decay sufficiently rapidly with rank for their cumulative contribution to remain bounded. This scenario is consistent with the experimental results presented above, where the eigenvalue slopes decay according to a power law, $b_{i}∼i^{-\left(1+\alpha \right)}$ with α>0. For ${\text{cos}}^{2}\theta_{i,N}\propto b_{i}^{\gamma }$ from Eq. 7, the linear-eigenvalue contribution reduces to $\sum_{i}b_{i}^{\gamma -1}$, and its summability, i.e., convergence of the summation $\sum_{i=1}^{N}i^{-\left(\gamma -1\right)\left(1+\alpha \right)}$, yields the critical exponent $\gamma_{c}$ given in Eq. 8. The full-spectrum calculation in text S3 further shows that the same condition ensures boundedness of the total LFI, including the cumulative contribution of the sublinear eigenvalues.

Furthermore, combining power-law eigenspectral scaling with the linear scaling of the total variance imposes important constraints on the power-law exponent and, consequently, on the boundedness of the LFI (Materials and Methods; full derivation in texts S5 and S6). Because the scale-invariant function is defined on $x_{i}=i/N\in \left[1/N,1\right]$, the *N* dependence enters through the left edge of the eigenspectrum, corresponding to the leading eigenvalues. Thus, the $x_{i}\to 0$ behavior determines the scaling of the total variance, although the variance is defined as the sum over the full eigenspectrum. The results are summarized in Table 1. Given the power-law form $g\left(x_{i}\right)∼x_{i}^{-\mu }$, we showed that the constraint of the linear total variance (Eq. 4) leads to μ=1+α if α is positive. In this regime, the leading eigenvalues scale linearly with population size, $\lambda_{i,N}∼N$ (for fixed *i*). The selected functional form for $g\left(x_{i}\right)$ in Eq. 6, specifically $g\left(x_{i}\right)∼x_{i}^{-\left(1+\alpha \right)}$ in the $x_{i}\to 0$ limit, is consistent with the linear scaling of the total variance (Eq. 4) and permits linear eigenvalues with α>0. Such linear scaling can potentially bound the LFI, provided that the corresponding eigenvectors align sufficiently with the signal direction.

**Table 1.** Scaling parameter, power-law exponent, and information boundedness under subsampling. For a scale-invariant power-law eigenspectrum, the rows summarize how the scaling parameter α constrains the power-law exponent μ, the scaling of the leading eigenvalues $\lambda_{i,N}$, and whether the LFI is bounded or unbounded under the linear scaling of the total variance.

Scaling parameter α, $\lambda_{i,N}=N^{-\alpha }g\left(x_{i}\right)\;\;\left(x_{i}=i/N\right)$	Power-law exponent μ, $g\left(x_{i}\right)∼x_{i}^{-\mu }\;\;\left(x_{i}\ll 1\right)$	Scaling of leading eigenvalues $\lambda_{i,N}∼N^{\mu -\alpha }\;\;\left(\text{fixed }i\right)$	LFI $I_{N}$
α>0	μ=1+α>1	$\lambda_{i,N}∼N$ (linear)	Bounded/unbounded
α=0	μ<1	$\lambda_{i,N}∼N^{\mu }$ (sublinear)	Unbounded

By contrast, when α=0, the same constraint imposes μ<1, indicating that the eigenspectrum must have a slope shallower than 1 if the eigenspectrum in the normalized index collapses in the limit of large *N*. In this case, the leading eigenvalues scale sublinearly, $\lambda_{i,N}∼N^{\mu }$ (for fixed *i*). This means that all eigenvalues scale sublinearly, making the information an unbounded function of *N*, regardless of the behavior of angular occupations (text S1). Notably, this yields a sufficient condition requiring only the noise eigenspectrum: given ${|f_{N}^{\;\prime }\left(s\right)|}^{2}∼N$, observing α=0 (equivalently μ<1) establishes unboundedness without estimating the signal-noise alignment. When neural activities examined in the previous section are individually normalized by their nonzero mean activities (*6*) or SDs (*4*, *5*), we observed that the slopes of leading eigenvalues satisfy μ<1 (fig. S8), further supporting that the LFI is not bounded in this dataset.

We note that, if a scale-invariant relationship between the eigenvalues and their angular occupations holds (Eq. 7), one obtains an analytical expression for the LFI (Eq. 9), from which its boundedness can be determined as discussed in the previous section.

**Fig. 3.**
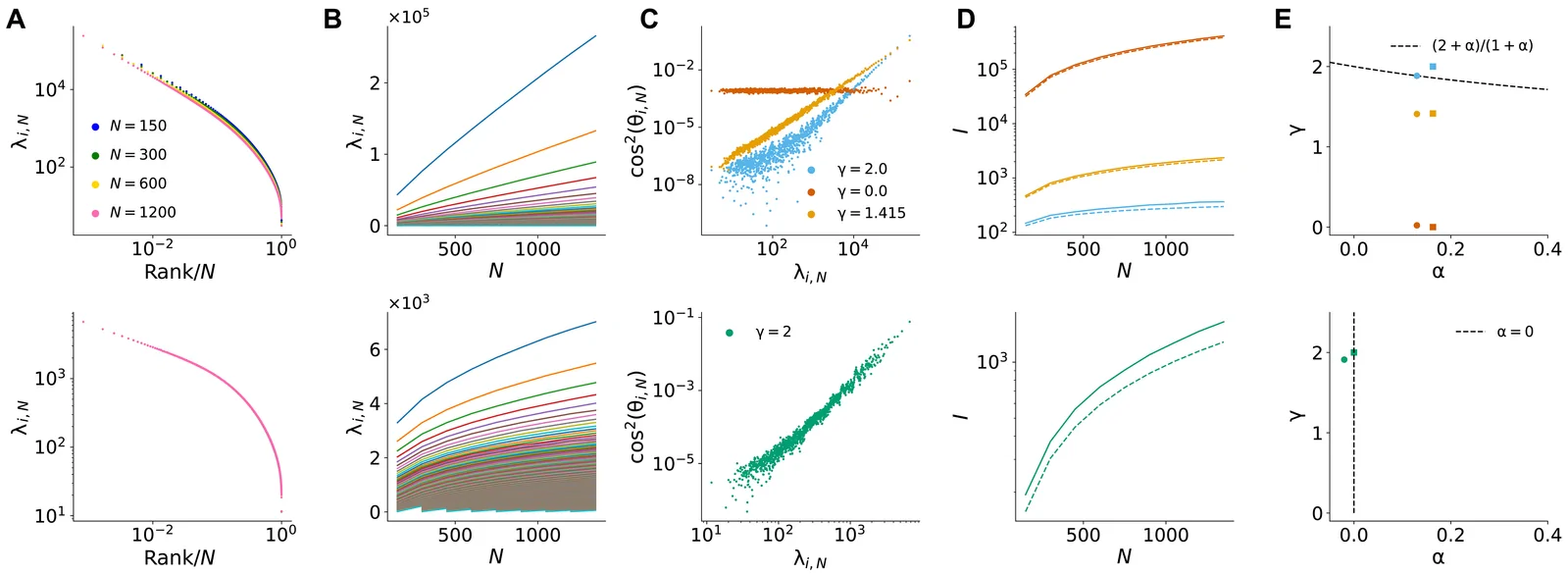
Verification of theory by simulated neural activity. (**A**) Eigenspectra (generated as described in the text for α=0.16 and α=0) as a function of normalized rank for simulated populations with the scaling parameter α=0.16 (observed value for mouse TX42, top) and α=0 (bottom) for *N* = 150, 300, 600, and 1200 neurons. (**B**) Eigenvalues as a function of the subpopulation size *N* with α=0.16 (top) and α=0 (bottom), showing the emergence of linear eigenvalues only when α>0. (**C**) Relation between the angular occupation ${\text{cos}}^{2}\theta_{i,N}$ and eigenvalue $\lambda_{i,N}$. Top: simulations with α=0.16 using Eq. 7 with γ=2 (a value resulting in bounded LFI for any α>0, blue), γ=0 (random signal, red), and γ=1.415 (observed value for mouse TX42, yellow). Bottom: simulation with α=0 and γ=2. Plots are shown for N=1200. (**D**) Theoretical (dashed) and bias-corrected (solid) estimates of the LFI (*17*, *25*) for the simulated populations. For α=0.16, the bias-corrected estimates show weaker growth for γ=2 than for γ=0 and γ=1.415, consistent with bounded and unbounded behaviors, respectively. For α=0, the bias-corrected estimate remains unbounded. (**E**) True (squares) and estimated (circles) values of γ as a function of α. The dashed lines indicate the critical boundaries. The bias correction methods were applied to this analysis.

We validated these predictions by simulating population activity of neurons using the scale-invariant eigenspectrum defined by Eqs. 5 and 6 for α>0 and a modified function for α=0 (Fig. 3A top and bottom, respectively). For the α=0 simulations, we replaced the leading-edge exponent 1+α in Eq. 6 with an independently chosen μ=0.5 while retaining the remaining parameters fitted to TX42. The simulation procedure is described in Materials and Methods. For the positive-α case, we used α=0.16, close to the estimate from the mouse data. We confirmed the emergence of linear eigenvalues with α>0 and their absence when α=0 (Fig. 3B). For α>0, we considered the signal and noise component directions given by Eq. 7 with γ=2 and γ=0 (Fig. 3C, top), which theoretically result in bounded and unbounded LFI, respectively. We also included an unbounded LFI example with γ=1.415, which is close to the estimate from the mouse data. For α=0, we expect unbounded LFI regardless of γ. Therefore, we consider an example with γ=2 (Fig. 3C, bottom), which would have resulted in the bounded LFI if α were positive.

We found that, as expected, the bias-corrected estimates of the LFI (*17*, *25*) show weaker growth for γ=2 than for γ=0 when α>0, consistent with bounded and unbounded behavior, respectively (Fig. 3D, top). For α=0, the bias-corrected estimate of the LFI is consistent with unbounded behavior (Fig. 3D, bottom). However, because the bias-corrected LFI is evaluated only over the observed range of population sizes, it cannot by itself reveal the asymptotic scaling behavior at larger *N*.

Alternatively, our approach uses the fitted scaling ansatz to predict the behavior of larger neuronal populations. We corroborated that the estimated scaling exponents α and γ are consistent with the theoretical criterion for LFI boundedness, with the inferred parameter pairs tracking the boundary between bounded and unbounded regimes (Fig. 3E). For α>0, we could correctly infer whether the LFI was bounded or unbounded from the estimated exponents, including for the experimentally observed value γ=1.415. When γ is large, however, it induces extremely small ${\text{cos}}^{2}\theta_{i,N}$ (Fig. 3C, top), making them difficult to estimate reliably from a limited number of samples. As a result, the inferred parameter pairs can be biased toward the theoretical boundary; therefore, the results in this regime should be interpreted with caution and may require more data. For α=0, by contrast, all eigenvalues remain sublinear under subsampling, so the LFI is theoretically unbounded regardless of γ (text S5). In this case, the estimated α was slightly negative (Fig. 3E, bottom), which can arise from finite-sample effects when the true α is zero and therefore indicates that the LFI is unbounded.

Last, when we applied the dimension reduction method to estimate the LFI (*16*) to simulated data whose LFI is theoretically unbounded, we observed saturating LFI (text S7 and figs. S9 and S10). These results demonstrate that apparent information saturation can arise as an artifact of the analysis method focusing on a restricted subspace, rather than as a property of the underlying population code, and highlight the importance of examining the full eigenvalue spectrum.

### Information-limiting correlations in subsampled populations are differential correlations

While we provided the scaling conditions for bounded and unbounded information under the linear scaling of the total variance, the connection between our theory and previously proposed information-limiting correlations needs clarification. Moreno-Bote *et al.* (*22*) introduced differential correlations as shared noise aligned with stimulus-dependent changes in the mean population response. Because this noise lies along the signal direction, it cannot be averaged away by adding neurons and can cause the LFI to saturate. More specifically, they showed that when the noise covariance has the following additive rank-one differential correlation structure, the LFI is bounded

$$\Sigma_{N}=\Sigma_{N}^{0}+\epsilon f_{N}^{\;\prime }{(s)f_{N}^{\;\prime }\left(s\right)}^{T}$$ (12)

where ϵ is a positive constant and $\Sigma_{N}^{0}$ is a covariance matrix whose corresponding LFI

$$I_{0}=f_{N}^{\;\prime }{\left(s\right)}^{T}{\left(\Sigma_{N}^{0}\right)}^{-1}f_{N}^{\;\prime }\left(s\right)$$ (13)

diverges with *N*. The resulting LFI is bounded above by $\epsilon^{-1}$ and is given by

$$I_{N}=\frac{I_{0}}{1+\epsilon I_{0}}$$ (14)

They further proposed that only differential correlations can limit the information. However, the original proof was restricted to specific noise covariances, leaving the claim unsubstantiated for general covariance matrices. Here, we prove that the information-limiting condition in our theory and the existence of differential correlations are not in general equivalent but become identical under the subsampling constraint, where we can observe subsamples of varying size *N* of a fixed population, i.e., the total population is fixed and does not grow. To this end, we first quantitatively explain how the addition of a subsampled neuron to a population changes or does not change the LFI.

Recursively adding a neuron to a population creates a sequence of subsampled populations whose covariance matrices form a nested structure, with each larger covariance matrix containing that of the smaller subsampled population. Given *N* neurons, consider adding a single neuron whose mean response is $f_{N+1}\left(s\right)$, variance $\Sigma_{N+1}$, and covariance $c_{N}$ with the existing *N* neurons. The covariance matrix of the resulting population of N+1 neurons is

$$\Sigma_{N+1}=\left[\begin{array}{cc} \Sigma_{N} & c_{N}\\ c_{N}^{T} & \Sigma_{N+1} \end{array}\right]$$ (15)

**Fig. 4.**
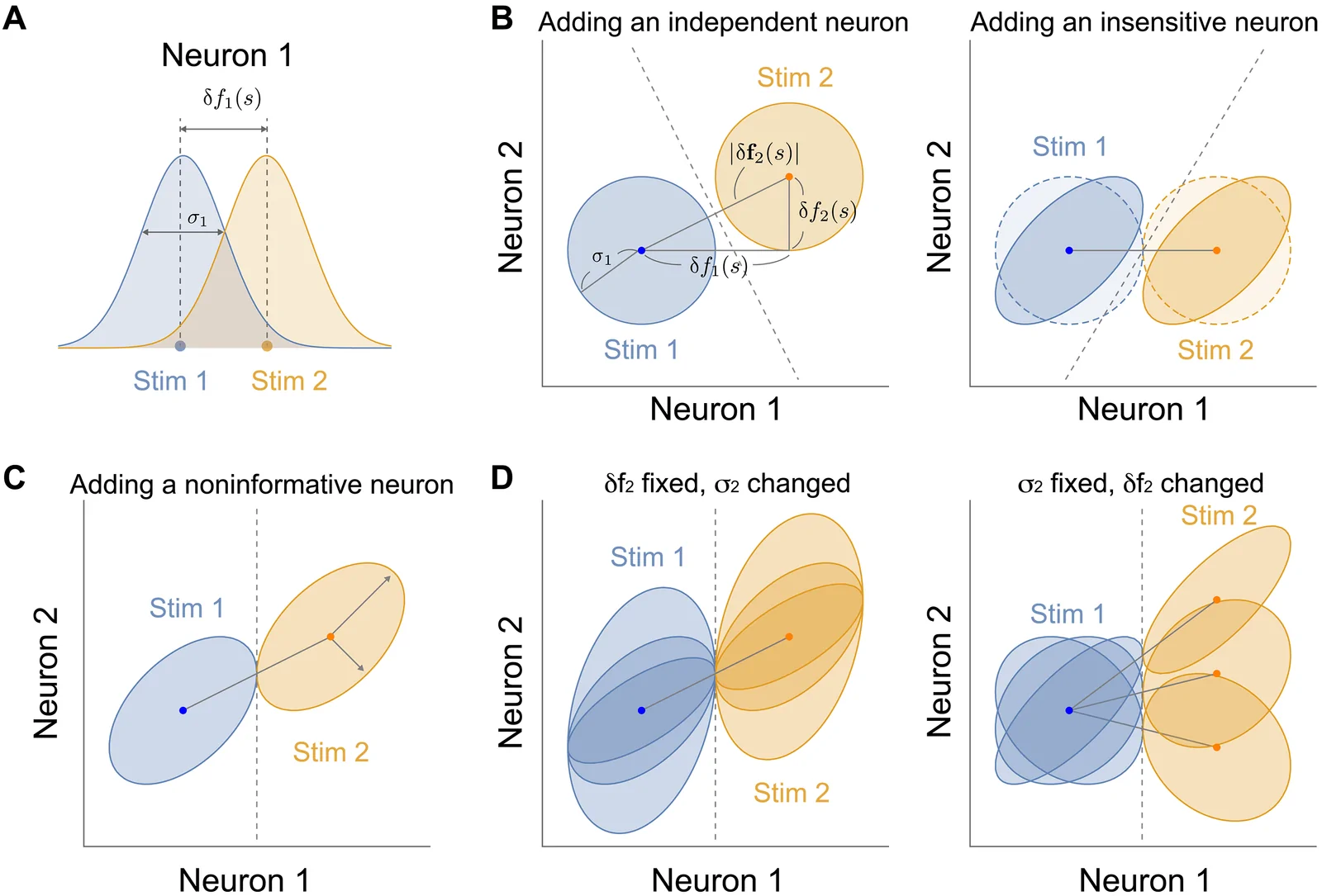
Incremental effect of adding a neuron on stimulus coding. (**A**) Response distributions of a single neuron (neuron 1) for two close stimuli (blue for stimulus 1 and orange for stimulus 2). The standardized mean difference $\delta f_{1}\left(s\right)/\sigma_{1}$ quantifies the ability to discriminate between the two stimuli. Here, $\delta f_{1}\left(s\right)=\sigma_{1}$. (**B**) Left: Joint distributions of two neurons after adding an independent, stimulus-sensitive neuron with the same variance ($\sigma_{2}=\sigma_{1}$). The dashed line indicates the optimal linear decision boundary. The circle radius indicates 1 SD. The distance between the mean responses for the two stimuli is $\left|\delta f_{2}\left(s\right)\right|=\sqrt{\delta f_{1}{\left(s\right)}^{2}+\delta f_{2}{\left(s\right)}^{2}}$. The horizontal separation between the two means remains $\delta f_{1}\left(s\right)=\sigma_{1}$, identical to (A). Right: joint distributions after adding a correlated, stimulus-insensitive neuron (neuron 2), having the same variance ($\sigma_{2}=\sigma_{1}$). Neuron 2 does not change the mean activity, but correlation makes the distributions ellipsoidal. The dashed line indicates the optimal decision boundary for the correlated neurons. Light-colored circles show a case with adding an independent, stimulus-insensitive neuron for comparison. (**C**) Joint distributions after adding a noninformative neuron (neuron 2). Adding a correlated, stimulus-sensitive neuron does not increase discriminability from the single neuron’s performance when $\left(\delta f_{1}\left(s\right)/\sigma_{1}^{2}\right)\left({\text{ρσ}}_{1}\sigma_{2}\right)=\delta f_{2}\left(s\right)$ with ρ being the correlation coefficient. We impose $\delta f_{1}^{2}/\sigma_{1}^{2}>\delta f_{2}^{2}/\sigma_{2}^{2}$ so that neuron 2 has smaller LFI than neuron 1. (**D**) Variations of the joint distributions that preserve discriminability. Left: $\delta f_{2}\left(s\right)$ is fixed, while $\sigma_{2}$ and correlation vary. Right: $\sigma_{2}$ is fixed, while $\delta f_{2}\left(s\right)$ and correlation vary.

Exploiting this nested structure, the LFI of N+1 neurons can be expressed recursively in terms of the LFI of *N* neurons as (text S8)

$$I_{N+1}=I_{N}+\omega_{N+1}^{-1}{\left[f_{N}^{\;\prime }{\left(s\right)}^{T}\Sigma_{N}^{-1}c_{N}-f_{N+1}^{\prime }\left(s\right)\right]}^{2}$$ (16)

where $\omega_{N+1}=\Sigma_{N+1}-c_{N}^{T}\Sigma_{N}^{-1}c_{N}$. For a single neuron whose response follows a normal distribution with a mean $f_{1}\left(s\right)$ and variance $\sigma_{1}^{2}\left(=\Sigma_{1}\right)$, the LFI is given by $I_{1}={\left(f_{1}^{\prime }\left(s\right)/\sigma_{1}\right)}^{2}$, which is the squared standardized mean difference of two normal distributions per unit squared stimulus difference in the finite difference case (Fig. 4A). Since $\omega_{N+1}>0$ is required for the covariance of N+1 neurons to be positive definite, Eq. 16 indicates that the LFI along this nested sequence of subpopulations is a nondecreasing function of *N*. Further, Eq. 16 reveals two distinct ways to increase the LFI (Fig. 4, A and B). First, when adding an independent neuron (i.e., $c_{N}=0$), the LFI increases by $\Sigma_{N+1}^{-1}f_{N+1}^{\prime }{\left(s\right)}^{2}$, which is the LFI of the added neuron (Fig. 4B, left). Second, the LFI increases even if $f_{N+1}^{\prime }\left(s\right)=0$; namely, even if we add a neuron whose activity is insensitive to the stimulus change, it will contribute to increasing information as long as $f_{N}^{\;\prime }{\left(s\right)}^{T}\Sigma_{N}^{-1}c_{N}\neq 0$. This is because adding a correlated neuron can remove the overlap of the noise distributions for the two close stimuli (Fig. 4B, right), explaining an effect found in the sensory and frontal areas of rodents and primates (*26*–*30*).

Moreover, the LFI does not increase if $f_{N}^{\;\prime }{\left(s\right)}^{T}\Sigma_{N}^{-1}c_{N}=f_{N+1}^{\prime }\left(s\right)$. Figure 4C presents an illustration of the joint responses of two neurons for a finite stimulus difference that satisfies this condition, with Fig. 4D showing additional variations. In all cases, when the LFI remains unchanged, the decision boundary (dashed line) is independent of the activity of the added neuron. Intuitively, to keep the information unchanged, the ratio between the noise magnitude along $f_{N}^{\;\prime }\left(s\right)$ and the distance between two stimuli, $|f_{N}^{\;\prime }\left(s\right)|$, must remain the same as in the single-neuron case shown in Fig. 4A. That is, the standardized mean difference along the $f_{N}^{\;\prime }\left(s\right)$ direction must be preserved. This intuition follows directly from the definition of LFI, which corresponds to the infinitesimal limit of the squared standardized mean difference along the direction that best discriminates the response distributions for the two stimuli (text S8).

On the basis of Eq. 16, we prove that, in general, the noise covariance of subsampled populations exhibiting increasing and saturating LFI with $I_{N}<I_{\infty }$ at every finite *N* can always be expressed in the form of Eq. 12. The proof follows by showing that $\Sigma_{N}^{0}=\Sigma_{N}-\epsilon f_{N}^{\;\prime }\left(s\right)f_{N}^{\;\prime }{\left(s\right)}^{T}$ is positive definite for $\epsilon =1/I_{\infty }$ (for more details, see text S9). In other words, such a noise covariance inherently contains differential correlations. This result justifies the widespread use of the concept of differential correlations in the analysis of subsampled populations (*24*, *31*, *32*).

Given the equivalence between differential correlation and information limiting under the subsampled conditions, we can reconfirm the necessity of the linear eigenvalues for the bounded LFI as a consequence of the differential correlations (*22*, *31*). Namely, the addition of differential correlations induces linear eigenvalues in the covariance matrix. The number of linear eigenvalues depends on whether the LFI associated with $\Sigma_{N}^{0}$ is unbounded for all signal directions or only for specific directions (text S10). However, to further achieve information boundedness, the alignment of the linear eigenvectors with the signal must occur faster than the rate set by the sublinear noise components (text S4) as well as the decay rate of the linear eigenvalue slopes with rank, i.e., boundedness requires each nonnegative LFI contribution to remain bounded. The above proof mitigates the need to directly search for differential correlations to confirm or refute the boundedness of information.

Last, this equivalence between information-limiting conditions and differential correlations is specific to subsampled populations. Differential correlations can be absent in more general noise covariances of nonsubsampled neuronal populations, even when the LFI approaches a finite limit (e.g., when it approaches that limit from above; text S11). Such a scenario might occur in growing neuronal populations, for example, through neurogenesis.

## DISCUSSION

We proposed a theory of population coding based on the scaling properties of neural systems, which allowed us to predict the boundedness of information. This theory critically considers the constraints introduced by subsampling, leading to the following key findings: (i) The squared distance between mean responses of randomly subsampled *N* neurons to two close stimuli increases proportionally to *N* (*16*, *18*). (ii) Random subsampling makes the total variance increase linearly with *N*, which allows a finite or countably infinite family of fixed-rank eigenvalue sequences to scale linearly, while eigenvalues whose ranks grow with *N* may scale sublinearly. (iii) For a finite linear family, the LFI can be limited if the sublinear eigenvalues’ angular occupation drops sufficiently rapidly. For an infinite linear family, the linear-eigenvalue contribution must additionally remain bounded. For a scale-invariant power-law eigenspectrum, these constraints further imply that (iv) an eigenspectrum with α>0 or α=0 necessitates that the spectrum decays with an exponent μ greater or less than one, respectively. (v) Only the scale invariance with α>0 allows for linear eigenvalues, which can potentially limit the information. (vi) By contrast, α=0 only allows for sublinear eigenvalues, resulting in unbounded LFI.

Through examination of the mouse data, we found the scale-invariant noise eigenspectra with α>0 (Fig. 2, D to F) and another power-law scaling relation between the eigenvalues and angular occupations with an exponent γ (Fig. 2G). Our analysis identified the critical exponent above and below which the LFI becomes bounded or remains unbounded. We found that the directions of the small noise components in the mouse data overlap with the signal direction at a sufficient rate, leading to a sublinear yet unbounded increase in LFI. Consequently, we conclude that the information encoded in the mouse V1 neurons can continue to increase without bound, going beyond the previous study on this dataset that reported no apparent signs of saturation in the LFI lower bound up to the recorded number of neurons (*21*).

Previous studies (*15*, *16*, *18*, *21*) reporting bounded information in the limit of large networks may be based on inadequate assumptions about covariance scaling. First, the study that collected the data analyzed here and reported high-precision coding (*21*) relied on extrapolation through a simple parametric scaling with $c_{1}+c_{2}/\sqrt{N}$ fitted to the decoding performance. Our results suggest that the reported bounded precisions in the limit of large *N* by a finite positive $c_{1}$ were conservative estimates, as predicted by the authors. Second, ignoring or regularizing the activities related to smaller variances can lead to different and potentially inaccurate conclusions. Rumyantsev *et al.* (*16*) reported saturating information using a dimension reduction method. However, applying the same method to simulated data whose LFI is theoretically unbounded, we observed saturating LFI (text S7 and figs. S9 and S10). Third, one cannot use a finite-size fit of Eq. 14 alone to infer bounded LFI. It is possible to find a saturating function that fits finite-size data well because $I_{0}$ may be any increasing unbounded function and ϵ is unknown (text S9). Therefore, excellent fits of Eq. 14 to empirical LFI (*15*, *16*, *18*) cannot, by themselves, establish asymptotic boundedness.

We found scale invariance with a positive α and a fast-decaying power-law exponent μ(=1+α)>1 in the recorded neural activities (Fig. 2, D to F, and figs. S1 to S5, D to F). In addition, we observed a scaling exponent μ<1 from the covariances of normalized neural activities (fig. S8), providing further evidence for unbounded information coding. This latter result aligns with other studies that have reported scale-invariant eigenspectra with scaling exponents less than 1 (*4*–*6*, *33*). While these results might indicate that noise in neural activities does not generally confound information coding, further rigorous assessment of the scaling parameters α and μ across brain regions and species is necessary to reveal the foundational coding capacities of neurons.

The ongoing activity in the mouse forebrain, including the visual cortex, is high dimensional (*1*). Furthermore, its reliable dimension increases without apparent saturation up to about a million neurons (*3*). These findings are consistent with the high-dimensional noise covariances because the ongoing activities driven by the animal’s behavior and internal dynamics are likely to cause variability in repeated stimulus-evoked activities (*34*–*36*). The above large-scale analyses showed that the ongoing activities overlap with signal correlations through one- or few-dimensional subspaces, suggesting multiplexed coding by the nearly orthogonal stimulus and spontaneous activities. The present study also supports that the noise correlations, including those arising from measurement noise, do not limit local signal representation. However, in agreement with positive correlations between the signal and noise in early sensory areas reported in other studies (*35*–*39*), we found broader directional overlaps between the leading noise components and signal (Fig. 2G), which is nevertheless insufficient to limit the information.

More generally, it remains unclear how the noise under brain states and behavioral conditions other than passive visual stimulation affects large-scale population coding. In smaller populations, it is known that cortical states (*40*), locomotion (*17*, *41*), and attention (*42*) modulate noise correlations in a way that improves coding capacity. We showed that population coding is affected by the scaling of smaller noise variances in the population activity and how their directions align with the signal direction. Thus, we cannot rule out the idea that even small changes caused by the behavior and internal dynamics (*5*) in small noise components of the high-dimensional noise have a substantial impact on the stimulus coding fidelity in large-scale neuronal populations.

## MATERIALS AND METHODS

### Analysis of mouse V1 data

We analyzed two-photon calcium imaging data of mouse V1 neurons from the “full-field” dataset by Stringer *et al.* (*21*) (see Data, code, and materials availability). The dataset includes recordings from five awake head-fixed mice, passively viewing static grating stimuli with up to ∼1000 trials per degree. Each trial presented a static grating stimulus for 750 ms, followed by a gray screen for ∼650 ms. The static grating stimuli shared the same spatial frequency (0.05 cycles per degree) with orientations ranging from 43° to 47°. The individual neural activities extracted by the Suite2p toolbox consisted of deconvolved fluorescence traces responding to the 750-ms stimulus presentation.

We divided the trials into two groups based on the orientation of the grating stimuli. Trials with orientations from 43° to 45°, including 45° ($s_{1}$), comprise the first group, while trials with orientations above 45° and up to 47° ($s_{2}$) comprise the second. The numbers of trials for stimuli 1 and 2, $L_{1}$ and $L_{2}$, differed by a small margin in all datasets (<50 trials in each dataset). We balanced the number of trials for both stimuli by randomly choosing $R=\text{min}\left\{L_{1},L_{2}\right\}$ trials (without replacement) from the stimulus with the larger number of trials. Following this procedure, the number of trials was 1855 to 2654. Each dataset had $N_{t}$ = 17,631 to 21,469 neurons.

We computed the activation means and covariance as follows. Let $x_{i}^{\left(r,j\right)}$ denote the activity of neuron *i* on trial r (=1,…,R) for stimulus group j∈{1,2}. For each group, we estimated the trial-averaged population activity as

$$\mu_{j}=\frac{1}{R}\sum_{r}x^{\left(r,j\right)}$$ (17)

and the covariance matrix as

$$S_{j}=\frac{1}{R-1}\sum_{r}\left(x^{\left(r,j\right)}-\mu_{j}\right){\left(x^{\left(r,j\right)}-\mu_{j}\right)}^{T}$$ (18)

We defined the unscaled mean-response difference as $\delta \mu =\mu_{2}-\mu_{1}$ and estimated ${\hat{f}}_{N}^{\;\prime }\left(s\right)$ as

$${\hat{f}}_{N}^{\;\prime }\left(s\right)=\frac{\delta \mu }{{\bar{s}}_{2}-{\bar{s}}_{1}}$$ (19)

where ${\bar{s}}_{j}$ denotes the trial-averaged stimulus orientation within group *j*. The population covariance was estimated as

$$S=\frac{S_{1}+S_{2}}{2}$$ (20)

To characterize the eigenvalues and angular occupations for a subsample of size *N*, we randomly selected 200 subpopulations of size *N* and averaged the resulting eigenvalues and angular occupations.

Since the raw activity traces of neurons might include confounding factors such as different expression levels of fluorescence proteins or positions of neurons in the scanning system, we also constructed the activation means and covariance using the standardized data, by normalizing each neuron’s activity across all trials by its SD (*4*, *5*) or by the mean of its nonzero activities (*6*).

### Fitting scaling parameters and a scale-invariant function to an eigenspectrum

The scaling parameter of the scale-invariant eigenvalues (Eq. 5) was fitted as follows. The eigenvalues were evaluated at population sizes spaced by 50 neurons. Given the scale invariance, we must have

$$\lambda_{i,N_{0}}=k^{\alpha }\lambda_{\mathrm{ki},{\mathrm{kN}}_{0}}\Rightarrow \text{log}\lambda_{i,N_{0}}=\alpha \text{log}k+\text{log}\lambda_{\mathrm{ki},{\mathrm{kN}}_{0}}$$ (21)

For each reference size $N_{0}$, we used $k=2,\ldots ,K\left(N_{0}\right)$, where $K\left(N_{0}\right)=$ $\left\lfloor N_{\text{avail}}/N_{0}\right\rfloor$ and $N_{\text{avail}}$ is the largest available population size. Therefore, we define the following cost function for α as

$$C=\sum_{N_{0}\in G}\sum_{k=2}^{K\left(N_{0}\right)}\sum_{i=1}^{N_{0}}{\left(\alpha \text{log}k+\text{log}\lambda_{\mathrm{ki},{\mathrm{kN}}_{0}}-\text{log}\lambda_{i,N_{0}}\right)}^{2}$$ (22)

The minimum of the cost function is obtained as a solution of $\frac{dC}{d\alpha }=0$ from which we obtain an estimated $\hat{\alpha }$

$$\hat{\alpha }=\frac{\sum_{N_{0}\in G}\sum_{k=2}^{K\left(N_{0}\right)}\sum_{i=1}^{N_{0}}\text{log}k\left(\text{log}\lambda_{i,N_{0}}-\text{log}\lambda_{\mathrm{ki},{\mathrm{kN}}_{0}}\right)}{\sum_{N_{0}\in G}\sum_{k=2}^{K\left(N_{0}\right)}N_{0}{\text{log}}^{2}k}$$ (23)

We computed $\hat{\alpha }$ for each recording using Eq. 23. The reference-size grid G began at 50 and extended up to one quarter of the maximum trial number.

We used the jackknife resampling method to obtain the estimation error of α. For each r=1,2,…,R, jackknife replicates ${\hat{\alpha }}_{r}$ were generated by computing the mean eigenvalues $\left\{\lambda_{i,N}^{r}\right\}$ over all but the *r*th repetition and using them in Eq. 23. Then, the jackknife estimate of the SD of $\hat{\alpha }$ was obtained as $\sqrt{\frac{R-1}{R}\sum_{r=1}^{R}{\left({\hat{\alpha }}_{r}-{\hat{\alpha }}_{\text{jack}}\right)}^{2}}$, where ${\hat{\alpha }}_{\text{jack}}=\frac{1}{R}\sum_{r=1}^{R}{\hat{\alpha }}_{r}$.

Using the estimated α, we obtained a collapsed curve by plotting $N^{\alpha }\lambda_{i,N}$ against $x_{i}=i/N$, using the mean eigenvalues over *R* repetitions. We then fitted the parametric scale-invariant function $g\left(x_{i}\right)$ (Eq. 6) to the collapsed eigenspectra in logarithmic coordinates, using all ranks and all evaluated population sizes.

The scaling parameter γ (Eq. 7) was estimated as follows. Using *t* randomly chosen trials for both stimuli (where *t* was varied from 500 up to the largest number of trials in the recording), we obtained γ(t,N) as the slope of a straight line fit to ${\text{cos}}^{2}\left(\theta_{i,N}\right)$ versus $\lambda_{i,N}$ in the log-log plot. For each *N*, we estimated $\gamma_{\infty }\left(N\right)$ as the *y* intercept of a straight line fit to the plot of γ(t,N) versus 1/t. The final estimate $\hat{\gamma }$ and its SD were obtained as the average and SD of $\gamma_{\infty }\left(N\right)$ for N>1000.

### LFI scaling under a power-law relation between angular occupation and eigenvalues

Here, we outline the derivation of the critical exponent $\gamma_{c}$ (Eq. 8) and the LFI scaling (Eq. 9); the full proof is given in text S3. Combining the LFI (Eq. 1) with the scale-invariant eigenspectrum $\lambda_{i,N}=N^{-\alpha }g\left(x_{i}\right)$ (α>0; Eq. 5) and the power-law relation between angular occupation and eigenvalues (Eq. 7) gives

$$\begin{array}{cc} I_{N}={|f_{N}^{\prime }\left(s\right)|}^{2}\;\frac{\Lambda_{N}\left(\gamma -1\right)}{\Lambda_{N}\left(\gamma \right)}, & \Lambda_{N}\left(\phi \right)\equiv \sum_{i}\lambda_{i,N}^{\phi } \end{array}$$ (24)

Using the leading-edge power law $g\left(x\right)∼x^{-\left(1+\alpha \right)}$, the integral approximation yields

$$\Lambda_{N}\left(\phi \right)\approx N^{1-\text{αϕ}}\int_{1/N}^{1}g^{\phi }\left(x\right)\;dx∼\{\begin{array}{ll} N^{\phi }, & \phi >1/\left(1+\alpha \right),\\ N^{\phi }\;\text{log }N, & \phi =1/\left(1+\alpha \right),\\ N^{1-\text{αϕ}}, & \phi <1/\left(1+\alpha \right) \end{array}$$ (25)

where the case split reflects whether the lower integration limit makes the integral diverge with *N*. Since $\Lambda_{N}\left(\gamma \right)∼N^{\gamma }$ and ${|f_{N}^{\;\prime }\left(s\right)|}^{2}∼N$ for γ>1, we have $I_{N}∼N^{1-\gamma }\Lambda_{N}\left(\gamma -1\right)$; substituting ϕ=γ−1 then reproduces Eq. 9, with the transition at γ−1=1/(1+α), i.e., $\gamma_{c}=\left(2+\alpha \right)/\left(1+\alpha \right)$ (Eq. 8).

### Bias correcting the eigenvalues and angular occupations

#### Eigenvalue bias correction

Finite-sample estimates of covariance matrices are known to produce biased eigenvalues: Small eigenvalues are typically underestimated, while large ones are overestimated (*43*). To address this, we developed an iterative shrinkage method. We began by computing a naive estimate of the covariance matrix, $\hat{\Sigma }$. This estimate was treated as a sample from a Wishart distribution

$$\hat{\Sigma }∼W\left(\frac{\Sigma }{2T-2},2T-2\right)$$ (26)

where Σ is the true covariance and *T* is the number of trials. Our goal was to infer the set of true eigenvalues, $\lambda_{i,N}^{\text{bc}}$, that could produce the observed biased sample eigenvalues.

Bias-corrected eigenvalues were obtained iteratively in the principal component basis, in which the true covariance is diagonal. Starting with the naive eigenvalues, ${\hat{\lambda }}_{i,N}$, as the initial guess, we repeatedly sampled synthetic covariance matrices from the Wishart distribution with the current covariance iterate as its mean

$$S_{j}^{\prime }∼W\left(\frac{\Lambda_{\text{bc}}}{2T-2},2T-2\right)$$ (27)

where $\Lambda_{\text{bc}}$ is the diagonal matrix of current eigenvalue estimates. The ranked eigenvalues of these synthetic matrices were averaged across samples to obtain ${\hat{\lambda }}_{i,N}^{\prime }$. These averages were then compared with the naive estimates, and the bias-corrected eigenvalues were updated according to

$$\lambda_{i,N}^{\text{bc}}\leftarrow \lambda_{i,N}^{\text{bc}}{\left(\frac{{\hat{\lambda }}_{i,N}}{{\hat{\lambda }}_{i,N}^{\;\prime }}\right)}^{\nu }$$ (28)

where ν is a small exponent controlling the update step.

This process was repeated until convergence, at which point the averaged synthetic eigenvalues matched the naive estimates. The resulting $\lambda_{i,N}^{\text{bc}}$ provide a bias-corrected spectrum that removes finite-sample bias from the covariance matrix. A detailed description of the method is presented in text S12.

#### Angular occupation bias correction

The error in estimating the angles $\theta_{i,N}$ between the vector δf and the eigenvectors of the covariance matrix $u_{i,N}$ leads to biased estimates of the angular occupations ${\text{cos}}^{2}\theta_{i,N}$. This bias arises from two sources. First, the estimated eigenvector ${\hat{u}}_{i,N}$ is oriented differently from the true eigenvector $u_{i,N}$. Second, the estimated vector δμ, used as an approximation of $f_{N}^{\;\prime }\left(s\right)$, has a different orientation from the true vector. We denote $\theta_{i,N}^{\mathrm{nv}}$, the naive estimate of $\theta_{i,N}$, as the angle between δμ and ${\hat{u}}_{i,N}$. Similarly, we denote the angle between δμ and the actual eigenvector $u_{i,N}$ as ${\tilde{\theta }}_{i,N}$. To correct the bias in angular occupations, we use a two-step method. First, we make the correction resulting from the error in the estimate of $u_{i,N}$; the goal of this step is to find ${\text{cos}}^{2}{\tilde{\theta }}_{i,N}$. In the second step, we correct ${\text{cos}}^{2}{\tilde{\theta }}_{i,N}$ for the bias caused by the error in the estimation of the signal. A detailed description of this step is presented in text S13.

##### Eigenvector bias correction.

 In the first step of angular occupation bias correction, we only have access to the naive estimates of the squared cosines ${\text{cos}}^{2}\theta_{i,N}^{\mathrm{nv}}$. To find ${\text{cos}}^{2}{\tilde{\theta }}_{i,N}$, we use an iterative method similar to the eigenvalue bias correction. Let ${\text{cos}}^{2}\theta_{i,N}^{\mathrm{nv}}$ denote the naive angular occupation estimates obtained from the estimated eigenvectors over *m* different subpopulations of the same size *N*. The goal of the algorithm is to use these naive estimates to recover bias-corrected values ${\text{cos}}^{2}{\tilde{\theta }}_{i,N}$ that more accurately reflect the alignment with the true covariance eigenvectors. We work in the same framework as in eigenvalue bias correction.

The algorithm proceeds as follows. First, the naive estimates are used as an initial guess for the bias-corrected squared cosines. We then generate *m* covariance matrices ${\hat{\Sigma }}_{j}$ from a Wishart distribution

$${\hat{\Sigma }}_{j}∼W\left(\frac{\Lambda_{\text{bc}}}{2T-2},2T-2\right)$$ (29)

where $\Lambda_{\text{bc}}$ is the diagonal matrix of bias-corrected eigenvalues obtained from the eigenvalue correction procedure. For each simulated covariance matrix, we compute the eigenvectors and their squared cosines with the candidate signal direction defined by the current angular-occupation estimates. These values are averaged across all *m* samples to produce predicted squared cosines ${\text{cos}}^{2}\theta_{i,N}^{\prime }$ that represent the expected naive estimates under the current guess of the true cosines.

The bias-corrected squared cosines are then updated according to

$${\text{cos}}^{2}{\tilde{\theta }}_{i,N}\leftarrow {\text{cos}}^{2}{\tilde{\theta }}_{i,N}{\left(\frac{{\text{cos}}^{2}\theta_{i,N}^{\mathrm{nv}}}{{\text{cos}}^{2}\theta_{i,N}^{\prime }}\right)}^{\xi }$$ (30)

where ξ serves as a learning rate controlling the update magnitude. The squared cosines are subsequently normalized so that

$$\sum_{i=1}^{N}{\text{cos}}^{2}{\tilde{\theta }}_{i,N}=1$$ (31)

Steps of simulation, averaging, updating, and normalization are repeated until convergence, defined as the condition ${\text{cos}}^{2}\theta_{i,N}^{\prime }\approx {\text{cos}}^{2}\theta_{i,N}^{\mathrm{nv}}$ is satisfied for all *i*.

This procedure yields bias-corrected squared cosines that account for sampling variability in the estimated eigenvectors and provide a more accurate representation of the alignment between δμ and the population eigenvectors. The resulting estimates can then be used in subsequent analyses requiring precise directional information, such as perturbative bias corrections or projections along principal components.

##### Signal bias correction.

 We modeled the observed signal difference δμ as the sum of the true underlying difference δf and additive Gaussian noise

η∼N(0,2Σ/T) (32)

where Σ is the known noise covariance and *T* is the number of trials. The eigenvectors $u_{i,N}$ and eigenvalues $\lambda_{i,N}$ of Σ were used to define principal directions of variability, and the alignment of the signal difference with each eigenvector was quantified by the squared cosine ${\text{cos}}^{2}\theta_{i,N}$. We define

$${\text{cos}}^{2}{\tilde{\theta }}_{i,N}=\frac{{\left(u_{i,N}^{T}\delta \mu \right)}^{2}}{{|\delta \mu |}^{2}}$$ (33)

which captures the fraction of the signal along each direction but is biased due to measurement noise.

To correct for this bias, we performed a perturbative expansion in 1/T, which shows that the expected value of the naive squared cosine is shifted by terms proportional to $\lambda_{i,N}$. Neglecting higher-order terms, the bias-corrected estimate is

$$\begin{array}{c} {\text{cos}}^{2}\theta_{i,N}^{\text{bc}}=\frac{{\text{cos}}^{2}{\tilde{\theta }}_{i,N}-q\lambda_{i,N}}{1-q\;[\mathrm{Tr}\Sigma -4\left({\bar{\lambda }}_{N}-\lambda_{i,N}\right)]},\\ q=\frac{2}{T\left({|\delta \mu |}^{2}-\frac{2}{T}\mathrm{Tr}\Sigma \right)} \end{array}$$ (34)

where ${\bar{\lambda }}_{N}=\sum_{j}\lambda_{j,N}{\text{cos}}^{2}\theta_{j,N}$. Because ${\bar{\lambda }}_{N}$ depends on the ${\text{cos}}^{2}\theta_{i,N}$ values themselves, we implemented a few iterations in which ${\bar{\lambda }}_{N}$ was updated using the current estimates of ${\text{cos}}^{2}\theta_{i,N}$. During iteration, any negative bias-corrected values were clipped to zero. This procedure yields nonnegative signal fractions along each principal direction that account for leading-order finite-sample noise.

### Scaling relations under linear scaling of the total variance

We sketch the derivation of the scaling relations summarized in Table 1; the full derivation is given in text S5. We write a scale-invariant eigenspectrum with a power-law leading edge as

$$\begin{array}{cc} \lambda_{i,N}=c\left(N\right)g\left(x_{i}\right), & x_{i}=\frac{i}{N}\in \left[1/N,1\right] \end{array}$$ (35)

where $x_{i}$ denotes the rank fraction. For small *x*, we assume a power-law leading edge

$$\begin{array}{cc} g\left(x\right)∼x^{-\mu } & \left(\mu >0,\;x\ll 1\right) \end{array}$$ (36)

The linearly scaling total variance then reads

$$V_{N}=\sum_{i=1}^{N}\lambda_{i,N}\approx N\;c(N)\int_{1/N}^{1}g\left(x\right)dx∼N$$ (37)

Thus, the *N* dependence of the integral, and hence the constraint on c(N), is determined by the small-*x* behavior of *g* through the lower integration limit

$$\int_{1/N}^{1}g\left(x\right)dx∼\{\begin{array}{ll} N^{\mu -1}, & \mu >1,\\ \text{log }N, & \mu =1,\\ \text{const}., & 0<\mu <1 \end{array}$$ (38)

For μ>1, this forces $c\left(N\right)=N^{-\alpha }$ with α=μ−1>0, giving linearly scaling leading eigenvalues $\lambda_{i,N}∼N^{\mu -\alpha }=N$. Such linear noise modes can bound the LFI only if the (also linearly growing) signal direction aligns with them; otherwise, the LFI is unbounded. For 0<μ<1, the integral converges, so c(N) is constant (α=0) and the leading eigenvalues scale sublinearly, $\lambda_{i,N}∼N^{\mu }$. In the absence of linearly scaling noise modes, the LFI is unbounded under the linear scaling of the signal ${|f_{N}^{\;\prime }|}^{2}∼N$. The two regimes correspond to the rows of Table 1 (α>0⇒μ=1+α, linear; α=0⇒μ<1, sublinear), with μ=1 a marginal case [$c\left(N\right)∼{\left(\text{log}N\right)}^{-1}$].

### Simulation of neural activity with bounded and unbounded LFI

We simulated neural responses to two stimuli by sampling from two multivariate Gaussian distributions, with means $f_{1}=\text{diag}\left(\Sigma \right)$ and $f_{2}=f_{1}+\Delta f$ and common covariance Σ

$$\begin{array}{cc} y^{\left(1\right)}∼N\left(f_{1},\Sigma \right), & y^{\left(2\right)}∼N\left(f_{2},\Sigma \right) \end{array}$$ (39)

We chose $f_{1}$ to approximate a Poissonian mean–variance relationship. We generated neural activity separately for each population size. The eigenvalues of Σ were obtained from Eqs. 5 and 6 for α>0 and from the modified α=0 eigenspectrum described in Results.

We constructed Σ using the following procedure: (i) sample reference firing rates $r_{i}$ from a lognormal distribution fitted to the mean responses in mouse TX42 data, (ii) construct a positive-definite candidate matrix M using the square root (elementwise) of the outer product of the sampled reference firing rates, with diagonal entries $r_{i}$ and off-diagonal entries $0.15\sqrt{r_{i}r_{j}}$; (iii) sample from the inverse-Wishart distribution with M as the mean; and (iv) obtain eigenvectors U of the resulting matrix and combine them with the diagonal matrix Λ of prescribed eigenvalues to construct $\Sigma =\mathrm{U\Lambda}U^{T}$. Using these eigenvectors, we generated the mean-response difference Δf using the positive square roots of the angular occupations given by Eq. 7. Specifically, we set $\Delta f=0.0025\left|f_{1}\right|\sum_{i=1}^{N}\text{cos}\theta_{i,N}\;u_{i,N}$.

### Ethics statement

This study used only previously published, publicly available data (*21*, *44*) and involved no new animal experiments. All original procedures were conducted under protocols reviewed by the IACUC, as reported in (*21*).
