# Supplementary material for: Population coding under the scale invariance of high-dimensional noise
Source: Sci Adv. 2026 Sep 25;12(39):eadz9632. doi: 10.1126/sciadv.adz9632 (PMC13614404; doi:10.1126/sciadv.adz9632)
Supplement: Supplementary file 1 [file sciadv.adz9632_sm.pdf]

Science Advances
AAAS

Supplementary Materials for

Population coding under the scale invariance of high-dimensional noise

S. Amin Moosavi *et al.*

Corresponding author: S. Amin Moosavi, moosavi.s.amin@gmail.com;
Hideaki Shimazaki, h.shimazaki@i.kyoto-u.ac.jp

Sci. Adv. **12**, eadz9632 (2026)
DOI: 10.1126/sciadv.adz9632

This PDF file includes:

Supplementary Text S1 to S13
Figs. S1 to S10

text S1. Linear Fisher information

In this note, we present the eigenvector representation of the linear Fisher information (LFI) for neuronal populations. This expression provides the basis for our later analysis of how covariance scaling and signal alignment determine whether information remains bounded or grows without bound.

Let $\mathbf{f}_N(s)$ be an N-dimensional vector whose elements are mean activity or tuning functions $f_i(s)$ ($i = 1, \ldots, N$) of individual neurons at a given stimulus s. Assuming that the covariance matrix of neuronal activity is invertible, the LFI about the stimulus s can be written as:

$$I_N = \mathbf{f}'_N(s)^{\mathrm{T}} \mathbf{\Sigma}_N^{-1} \mathbf{f}'_N(s) \tag{S1.1}$$

where $\mathbf{f}'_N(s)$ is the N-dimensional vector composed of the derivatives of tuning functions with respect to s, assuming that the tuning functions are differentiable, and $\mathbf{\Sigma}_N$ is the covariance matrix.

We denote the i-th eigenvalue of the covariance matrix $\mathbf{\Sigma}_N$ in the N-dimensional space as $\lambda_{i,N}$. Here we index the eigenvalues in a descending order (from large to small): $\lambda_{1,N} \geq \lambda_{2,N} \geq \cdots \geq \lambda_{N,N}$. We write the covariance matrix in terms of its eigenvalues $\lambda_{i,N}$ and eigenvectors $\mathbf{u}_{i,N}$ as

$$\mathbf{\Sigma}_N = \sum_{i=1}^{N} \lambda_{i,N} \mathbf{u}_{i,N} \mathbf{u}_{i,N}^{\mathrm{T}}, \tag{S1.2}$$

where T is the transposition operator. We can also write $\mathbf{f}'_N(s)$ as a linear combination of eigenvectors of the covariance matrix as

$$\mathbf{f}'_N(s) = |\mathbf{f}'_N(s)| \sum_{i=1}^{N} \cos\theta_{i,N} \mathbf{u}_{i,N}, \tag{S1.3}$$

where $\theta_{i,N}$ is the angle between the vector $\mathbf{f}'_N(s)$ and the i-th eigenvector of the covariance matrix $\mathbf{u}_{i,N}$. Using the above equations, we can rewrite the LFI as

$$I_N = \sum_{i=1}^{N} \frac{|\mathbf{f}'_N(s)|^2}{\lambda_{i,N}} \cos^2\theta_{i,N}. \tag{S1.4}$$

Eq. S1.4 indicates that whether the LFI is limited or not depends on the combination of the scaling of $|\mathbf{f}'_N(s)|^2$, the eigenvalue $\lambda_{i,N}$, and the angular occupation $\cos^2\theta_{i,N}$. Each obeys a certain constraint, resulting in a characteristic scaling structure. Most importantly, the signal magnitude $|\mathbf{f}'_N(s)|^2$ is expected to scale with N as long as each element $f'_i(s)^2$ is sampled from a common distribution having a finite nonzero mean η because $E|\mathbf{f}'_N(s)|^2 = E\sum_i f'_i(s)^2 = N\eta$.

Although the LFI behavior with respect to the population size N generally depends on how the eigenvalues and angular occupations scale relative to the linearly scaling signal magnitude, a simple conclusion can be drawn without knowing the angular occupations when all eigenvalues scale sublinearly. In this case, the largest eigenvalue satisfies $\lambda_{1,N}/N \to 0$ as $N \to \infty$. Since $\lambda_{i,N} \leq \lambda_{1,N}$ and $\sum_{i=1}^{N} \cos^2\theta_{i,N} = 1$, Eq. S1.4 implies

$$I_N \geq \frac{|\mathbf{f}'_N(s)|^2}{\lambda_{1,N}}. \tag{S1.5}$$

If $|\mathbf{f}'_N(s)|^2$ scales linearly with N, the right-hand side diverges. Thus, in this special case, the LFI is unbounded regardless of the signal direction.

text S2. Linear scaling of the total variance constrains the scaling of eigenvalues

In this note, we discuss the linear scaling of the total variance in subsampled neurons, categorize the scaling behavior of eigenvalues, and outline constraints on eigenvalue scaling when the total variance scales linearly.

For any number of neurons N, the total variance V_N is equal to the sum of variances of individual neurons, which is also equal to the sum of all eigenvalues of the covariance matrix, i.e.,

$$V_N = \sum_{i=1}^{N} \sigma_{i,N}^2 = \sum_{i=1}^{N} \lambda_{i,N}. \tag{S2.1}$$

Here $\sigma_{i,N}$ is the standard deviation in the activity of neuron i in a population of size N. In a subsampled population of neurons, the variance of an individual neuron's activity is independent of the subpopulation size N, i.e., $\sigma_{i,N}^2 = \sigma_i^2$. We can consider a distribution of σ_i^2 across neurons with a positive mean μ_{σ^2} and a bounded support, i.e., the variance of a given neuron is nonnegative by definition and cannot exceed an upper bound by physiological constraints. Looking at the variability in the activity of a randomly selected neuron in recordings of a neuronal population is equivalent to sampling σ_i^2 from its distribution. Under this random-sampling model, the law of large numbers gives $V_N/N = N^{-1}\sum_{i=1}^{N}\sigma_i^2 \to \mu_{\sigma^2}$ as $N \to \infty$. Because μ_{σ^2} is finite and nonzero, we have

$$V_N \sim N. \tag{S2.2}$$

This adds important constraints on the scaling of the eigenvalues with respect to N.

To see this, we categorize the eigenvalues into those that scale linearly, sublinearly, and supralinearly, defined as follows. For each fixed rank $j \in \mathbb{N}$, consider the eigenvalue sequence $\{\lambda_{j,N}\}_{j\le N}$, and assume that $\lim_{N\to\infty} \lambda_{j,N}/N$ exists as an extended nonnegative real number. The set of indices corresponding to linearly scaling eigenvalues is defined as

$$L = \{ j \in \{1,2,\dots,N\} \,|\, \lambda_{j,N} = F_j(N) + b_j N \}, \tag{S2.3}$$

where $b_j > 0$ is a constant positive number, and $F_j(N)$ is a function satisfying

$$\lim_{N\to\infty} \frac{F_j(N)}{N} = 0. \tag{S2.4}$$

Here, we refer to $\lambda_{j,N} = F_j(N) + b_j N$ as linear eigenvalues because the linear term will dominate the behavior at large N. Next, we define a set of sublinear eigenvalues that grow slower than the linear function in the limit as

$$S_b = \{ j \in \{1,2,\dots,N\} \,|\, \lambda_{j,N} = G_j(N) \}, \tag{S2.5}$$

where $G_j(N)$ is a function satisfying

$$\lim_{N\to\infty} \frac{G_j(N)}{N} = 0. \tag{S2.6}$$

The sublinear eigenvalues may increase sublinearly, remain constant, or decrease as a function of N. Similarly, we define a set of indices corresponding to supralinear eigenvalues as

$$S_p = \{ j \in \{1,2,\dots,N\} \,|\, \lambda_{j,N} = H_j(N) \}, \tag{S2.7}$$

in which $H_j(N)$ is a function of N with the following property

$$\lim_{N\to\infty} \frac{H_j(N)}{N} = \infty. \tag{S2.8}$$

Given these definitions, an immediate consequence of the constraint given by Eq. S2.2 is that the supralinear fixed-rank eigenvalue sequences cannot exist under the linear scaling of the total variance. For linear eigenvalues, if b_j values exhibit a constant positive lower bound $b_j > b_0 > 0$, the number of linear eigenvalues must converge to a fixed finite number as N increases, i.e., the

set L is finite. Otherwise, if the number of linear eigenvalues increases with N, their summed contribution to the total variance grows faster than linearly. For example, if their number grows as N^{ϕ}, the lower bound $b_j > b_0$ implies that their summed contribution is at least $b_0 N^{\phi+1}$, which is supralinear for $\phi > 0$. Thus, the number of linear eigenvalues must remain finite. On the other hand, if the b_j values decrease with rank sufficiently rapidly to be summable, then the contribution of the linear eigenvalues is compatible with linear total-variance scaling. For example, $b_j \sim j^{-(1+\alpha)}$ is summable when $\alpha > 0$.

In sum, at large N, if $b_j > b_0 > 0$, the number of elements in set L approaches a constant integer $c \geq 0$, and consequently the number of elements in set S_b linearly increases with N, while the set S_p becomes empty, i.e.,

$$\lim_{N\to\infty} |L| = c, \quad \lim_{N\to\infty} |S_b| = \infty \ , \ \lim_{N\to\infty} |S_p| = 0. \tag{S2.9}$$

On the other hand, if L is countably infinite, linear total-variance scaling requires its positive slopes to be summable:

$$\sum_{j\in L} b_j < \infty. \tag{S2.10}$$

text S3. Scaling of LFI under a scale-invariant eigenspectrum with a power-law relation to the angular occupation

Considering the scaling ansatz in Eqs. 5 and 6 with $\alpha > 0$ together with the following power-law relation (Eq. 7):

$$\cos^2\theta_i = \frac{\lambda_i^\gamma}{\sum_{i'=1}^N \lambda_{i'}^\gamma}, \tag{S3.1}$$

we can write the LFI as

$$I = |\mathbf{f}_N'(s)|^2 \frac{\sum \lambda_i^{\gamma-1}}{\sum \lambda_i^\gamma}. \tag{S3.2}$$

In this note, we establish an analytical expression of the above LFI and prove that the information is a bounded function of N if

$$\gamma > \frac{2+\alpha}{1+\alpha}. \tag{S3.3}$$

Before proving the above statement, we argue, more generally, that regardless of the scaling of the eigenvalues, if $\gamma = 1$, we have

$$I = |\mathbf{f}_N'(s)|^2 \sum_{i=1}^N \frac{\cos^2\theta_i}{\lambda_i} = |\mathbf{f}_N'(s)|^2 \sum_{i=1}^N \frac{1}{\sum_{j=1}^N \lambda_j} = \frac{|\mathbf{f}_N'(s)|^2}{\sum_{j=1}^N \lambda_j} \sum_{i=1}^N 1 \sim N \tag{S3.4}$$

Therefore, for $\gamma = 1$, the information is a linearly increasing function of N. The last step is obtained by considering the fact that

$$|\mathbf{f}_N'(s)|^2 \sim N \ \text{ and } \ \sum_{j=1}^N \lambda_j \sim N. \tag{S3.5}$$

See text S2 for more details. On the other hand, if $\gamma = 2$,

$$I = |\mathbf{f}_N'(s)|^2 \sum_{i=1}^N \frac{\cos^2\theta_i}{\lambda_i} = |\mathbf{f}_N'(s)|^2 \sum_{i=1}^N \frac{\lambda_i}{\sum_{j=1}^N \lambda_j^2} = \frac{|\mathbf{f}_N'(s)|^2 \sum_{i=1}^N \lambda_i}{\sum_{j=1}^N \lambda_j^2} \sim \frac{N^2}{N^2} \tag{S3.6}$$

that is a bounded function of N. It is clear that the numerator, which is the product of the total variance and the squared norm of $\mathbf{f}_N'(s)$, scales as N^2. The denominator scaling, however, needs more attention. Since

$$\sum_{i=1}^N \lambda_i^2 < \left(\sum_{i=1}^N \lambda_i\right)^2 \sim N^2, \tag{S3.7}$$

we have $\left(\sum_{i=1}^N \lambda_i\right)^2$ as an upper bound of $\sum_{i=1}^N \lambda_i^2$. In addition, if there exists at least one eigenvalue that is a linearly increasing function of N, say $\lambda_1 \sim N$, then we have

$$\sum_{i=1}^N \lambda_i^2 > \lambda_1^2 \sim N^2 \tag{S3.8}$$

that specifies a lower bound for $\sum_{i=1}^N \lambda_i^2$; as a result, because both the upper and lower bounds scale with N^2, we can conclude that

$$\sum_{i=1}^N \lambda_i^2 \sim N^2. \tag{S3.9}$$

Up to this point, we showed that for $\gamma = 1$ the information is unbounded and for $\gamma = 2$ it is bounded. An immediate conclusion is that there must exist a $1 < \gamma < 2$ at which we have a transition from unbounded to bounded information. In the rest of this note, we prove that the critical value of $\gamma = \gamma_c$ at which the transition happens is identified by the following exponent relation:

$$\gamma_c = \frac{2+\alpha}{1+\alpha}. \tag{S3.10}$$

To prove that, we first focus on the scaling of

$$\Lambda(\phi) = \sum_{i=1}^N \lambda_i^\phi \tag{S3.11}$$

in which ϕ is a positive exponent. Considering the scaling ansatz (Eq. 5) with the scale-invariant function $g(x)$ defined in Eq. 6, we can write

$$\Lambda(\phi) = N^{-\alpha\phi} \sum_{i=1}^{N} g^{\phi}(x_i). \tag{S3.12}$$

Using the integral approximation of the summation, we have

$$\Lambda(\phi) \approx N^{1-\alpha\phi} \int_{1/N}^{1} g^{\phi}(x)dx = N^{1-\alpha\phi} \left[\int_{1/N}^{x_0} g^{\phi}(x)dx + \int_{x_0}^{1} g^{\phi}(x)dx \right], \tag{S3.13}$$

in which x_0 is a fixed small number ($1/N < x_0 \ll 1$) for which we can approximate

$$g(x) \approx \mathrm{e}^{b+c} x^{-1-\alpha}. \tag{S3.14}$$

In Eq. S3.13, the second integral in the brackets is independent of N, and the first integral can be approximated as

$$\int_{1/N}^{x_0} g^{\phi}(x)\mathrm{d}x \approx \int_{1/N}^{x_0} \mathrm{e}^{(c+b)\phi} x^{-\phi(1+\alpha)} \mathrm{d}x. \tag{S3.15}$$

By integrating directly and substituting the result into Eq. S3.13, we get

$$\Lambda(\phi) \sim \begin{cases} N^{\phi} & \phi > 1/(1+\alpha), \\ N^{\phi} \log N & \phi = 1/(1+\alpha), \\ N^{1-\alpha\phi} & \phi < 1/(1+\alpha). \end{cases} \tag{S3.16}$$

See below for the derivations.

Using Eqs. S3.2 and S3.16, we can rewrite the LFI in terms of the function $\Lambda(\gamma)$ and $\Lambda(\gamma-1)$ as

$$I = |\mathbf{f}'_N(s)|^2 \frac{\Lambda(\gamma-1)}{\Lambda(\gamma)} \sim N^{1-\gamma} \Lambda(\gamma-1) \tag{S3.17}$$

because $\Lambda(\gamma) \sim N^{\gamma}$ for $\gamma > 1$. Using Eq. S3.16, we can write

$$I \sim \begin{cases} N^{0} & \gamma > (2+\alpha)/(1+\alpha), \\ \log N & \gamma = (2+\alpha)/(1+\alpha), \\ N^{2-\alpha(\gamma-1)-\gamma} & 1 < \gamma < (2+\alpha)/(1+\alpha). \end{cases} \tag{S3.18}$$

That is an unbounded function of N for $1 < \gamma \leq (2+\alpha)/(1+\alpha)$ and bounded for $\gamma > (2+\alpha)/(1+\alpha)$.

Finally, we note that $\cos^2\theta_{i,N}$ is computed as

$$\cos^2\theta_{i,N} \sim \begin{cases} \left(\lambda_{i,N}/N\right)^{\gamma} & \gamma > 1/(1+\alpha), \\ (\log N)^{-1} \left(\lambda_{i,N}/N\right)^{\gamma} & \gamma = 1/(1+\alpha), \\ N^{-1} \left(N^{\alpha}\lambda_{i,N}\right)^{\gamma} & \gamma < 1/(1+\alpha), \end{cases} \tag{S3.19}$$

which exhibits the scale invariance except for $\gamma = 1/(1+\alpha)$:

$$\cos^2\theta_{\delta i,\delta N} \sim \begin{cases} \delta^{-\gamma(1+\alpha)} \cos^2\theta_{i,N} & \gamma > 1/(1+\alpha), \\ \delta^{-1} \cos^2\theta_{i,N} & \gamma < 1/(1+\alpha). \end{cases} \tag{S3.20}$$

Derivation of $\Lambda(\phi)$ is as follows:

Case of $\phi > 1/(1+\alpha)$:

$$\Lambda(\phi) \approx N^{1-\alpha\phi} \int_{1/N}^{1} g^{\phi}(x)dx = N^{1-\alpha\phi} \left[\int_{1/N}^{x_0} g^{\phi}(x)dx + \underbrace{\int_{x_0}^{1} g^{\phi}(x)dx}_{Q(x_0,\phi)} \right]. \tag{S3.21}$$

Using the approximation of $g(x)$ for small x, we have

$$\int_{1/N}^{x_0} g^{\phi}(x)\mathrm{d}x \approx \mathrm{e}^{(c+b)\phi} \int_{1/N}^{x_0} x^{-\phi(1+\alpha)}\mathrm{d}x = \frac{\mathrm{e}^{(c+b)\phi}}{\phi(1+\alpha)-1} \left[N^{\phi(1+\alpha)-1} - x_0^{1-\phi(1+\alpha)} \right]. \tag{S3.22}$$

Replacing in Eq. S3.21, we get

$$\Lambda(\phi) \approx N^{1-\alpha\phi} \left[\frac{\mathrm{e}^{(c+b)\phi}}{\phi(1+\alpha)-1} \left(N^{\phi(1+\alpha)-1} - x_0^{1-\phi(1+\alpha)} \right) + Q(x_0,\phi) \right] \tag{S3.23}$$

$$= \frac{\mathrm{e}^{(c+b)\phi}}{\phi(1+\alpha)-1} N^{\phi} + N^{1-\alpha\phi} \left[Q(x_0,\phi) - \frac{\mathrm{e}^{(c+b)\phi}}{\phi(1+\alpha)-1} x_0^{1-\phi(1+\alpha)} \right]. \tag{S3.24}$$

Because $\phi > 1 - \alpha\phi$, in the large-N limit

$$\Lambda(\phi) \sim N^{\phi}. \tag{S3.25}$$

Case of $\phi = 1/(1+\alpha)$:

$$\Lambda(\phi) \approx N^{1-\alpha\phi} \int_{1/N}^{1} g^{\phi}(x)dx = N^{1-\alpha\phi} \left[\int_{1/N}^{x_0} g^{\phi}(x)dx + \underbrace{\int_{x_0}^{1} g^{\phi}(x)dx}_{Q(x_0,\phi)} \right]. \tag{S3.26}$$

Using the approximation of $g(x)$ for small x, we have

$$\int_{1/N}^{x_0} g^{\phi}(x)\mathrm{d}x \approx \mathrm{e}^{(c+b)\phi} \int_{1/N}^{x_0} x^{-\phi(1+\alpha)}\mathrm{d}x = \mathrm{e}^{(c+b)\phi} \int_{1/N}^{x_0} x^{-1}\mathrm{d}x = \mathrm{e}^{(c+b)\phi} \left[\log x_0 + \log N \right]. \tag{S3.27}$$

Replacing in Eq. S3.26, we get

$$\Lambda(\phi) \approx N^{1-\alpha\phi} \left[\mathrm{e}^{(c+b)\phi} \left(\log x_0 + \log N \right) + Q(x_0,\phi) \right] \tag{S3.28}$$

$$= N^{\phi} \left[\mathrm{e}^{(c+b)\phi} \left(\log x_0 + \log N \right) + Q(x_0,\phi) \right]. \tag{S3.29}$$

In the last step we used $1 - \alpha\phi = \phi$. Therefore, in the large-N limit

$$\Lambda(\phi) \sim N^{\phi} \log N. \tag{S3.30}$$

Case of $\phi < 1/(1+\alpha)$:

$$\Lambda(\phi) \approx N^{1-\alpha\phi} \int_{1/N}^{1} g^{\phi}(x)dx = N^{1-\alpha\phi} \left[\int_{1/N}^{x_0} g^{\phi}(x)dx + \underbrace{\int_{x_0}^{1} g^{\phi}(x)dx}_{Q(x_0,\phi)} \right]. \tag{S3.31}$$

Using the approximation of $g(x)$ for small x, we have

$$\int_{1/N}^{x_0} g^{\phi}(x)\mathrm{d}x \approx \mathrm{e}^{(c+b)\phi} \int_{1/N}^{x_0} x^{-\phi(1+\alpha)}\mathrm{d}x = \frac{\mathrm{e}^{(c+b)\phi}}{1-\phi(1+\alpha)} \left[x_0^{1-\phi(1+\alpha)} - N^{\phi(1+\alpha)-1} \right]. \tag{S3.32}$$

Replacing in Eq. S3.31, we get

$$\Lambda(\phi) \approx N^{1-\alpha\phi} \left[\frac{\mathrm{e}^{(c+b)\phi}}{1-\phi(1+\alpha)} \left(x_0^{1-\phi(1+\alpha)} - N^{\phi(1+\alpha)-1} \right) + Q(x_0,\phi) \right] \tag{S3.33}$$

$$= \frac{-\mathrm{e}^{(c+b)\phi}}{1-\phi(1+\alpha)} N^{\phi} + N^{1-\alpha\phi} \left[Q(x_0,\phi) + \frac{\mathrm{e}^{(c+b)\phi}}{1-\phi(1+\alpha)} x_0^{1-\phi(1+\alpha)} \right]. \tag{S3.34}$$

Because $\phi < 1 - \alpha\phi$, in the large-N limit

$$\Lambda(\phi) \sim N^{1-\alpha\phi}. \tag{S3.35}$$

text S4. Scaling conditions for bounded and unbounded LFI

In this note, we derive the necessary and sufficient conditions on the covariance matrix that limit information about a stimulus when the size of a neuronal population N increases, given that the covariance matrices exhibit the linear scaling of the total variance with respect to N. Here, we assume the slopes of linear eigenvalues exhibit a positive lower bound that leads to a finite number of linear eigenvalues under the linear scaling of the total variance.

First, as explained in text S1, $|\mathbf{f}'_N(s)|^2$ is expected to scale with N

$$|\mathbf{f}'_N(s)|^2 \sim N. \tag{S4.1}$$

Second, the angular occupation is constrained by

$$\sum_{i=1}^{N} \cos^2\theta_{i,N} = 1. \tag{S4.2}$$

We will explain the consequences of this constraint on angular occupation at the end of this note.

Third, the total sum of eigenvalues for the subsampled population scales linearly:

$$\sum_{i=1}^{N} \lambda_{i,N} \sim N \tag{S4.3}$$

As discussed in text S2, under the assumed positive lower bound on the linear-eigenvalue slopes, this constraint requires $|L|$ to remain finite and $|S_b|$ to increase linearly with N, while prohibiting supralinear eigenvalues. Throughout this note, L denotes the set of linearly scaling eigenvalues, and we assume $|L| < \infty$.

The LFI based on the above definitions can be written in terms of the contributions of the two defined sets as

$$\begin{aligned} I_N &= |\mathbf{f}'_N(s)|^2 \sum_{i=1}^{N} \frac{\cos^2\theta_{i,N}}{\lambda_{i,N}} \\ &= \sum_{i\in L} \frac{AN\cos^2\theta_{i,N}}{\lambda_{i,N}} + \sum_{i\in S_b} \frac{AN\cos^2\theta_{i,N}}{\lambda_{i,N}}. \end{aligned} \tag{S4.4}$$

where we used $|\mathbf{f}'_N(s)|^2 = AN$. For future reference, we define

$$W_L(N) = \sum_{j\in L} \cos^2\theta_{j,N}, \quad W_{S_b}(N) = \sum_{j\in S_b} \cos^2\theta_{j,N}, \tag{S4.5}$$

as the total angular occupation by these two sets.

Because the two summation terms on the right-hand side of Eq. S4.4 are nonnegative, the LFI is bounded if and only if both terms remain bounded. Here, we study the two terms. Using the linear-eigenvalue definition $\lambda_{i,N} = F_i(N) + b_i N$ for $i \in L$ from text S2 (Eq. S2.3), together with $\cos^2\theta_{i,N} \leq 1$, the first term is bounded as

$$I_L(N) = \sum_{i\in L} \frac{A\cos^2\theta_{i,N}}{\frac{F_i(N)}{N} + b_i} \leq \sum_{i\in L} \frac{A}{\frac{F_i(N)}{N} + b_i}. \tag{S4.6}$$

Hence, applying the definition of the sublinear function (Eq. S2.4), individual terms in the summation on the right-hand side of the equation above approach a finite value in the large-N limit. Since the number of linear eigenvalues $|L|$ is finite by the finite-L assumption of this note, their sum is finite. Therefore, $I_L(N)$ is bounded, regardless of the behavior of $\cos^2\theta_{i,N}$ as a function of N.

The second term on the right-hand side of Eq. S4.4 is

$$I_{S_b}(N) = A \sum_{i\in S_b} \cos^2\theta_{i,N} \frac{N}{\lambda_{i,N}}, \tag{S4.7}$$

where $\lambda_{i,N} = G_i(N)$ for $i \in S_b$ (Eq. S2.6). Based on the definition of S_b, $N/\lambda_{i,N}$ in the above summation are unbounded functions of N. Therefore, to have an upper bound for $I_{S_b}(N)$, it is necessary

that for all terms in this summation, $\cos^2\theta_{i,N}$ must decay asymptotically at least as fast as $\lambda_{i,N}/N$. Not only that, the summation itself must remain bounded. In other words, to have an upper bound on $I_{S_b}(N)$, the required asymptotic decay of $\cos^2\theta_{i,N}$ ($\forall i \in S_b$) as a function of N depends not only on the behavior of $\lambda_{i,N}/N$ but also on the number of terms in the summation, i.e., the number of elements in S_b. Note that, under the assumptions of this note, the number of sublinear noise components $|S_b|$ increases linearly with N.

Accordingly, the following two conditions comprise the necessary and sufficient conditions for bounded information. First, $W_{S_b}(N) = 0$ is sufficient for information to be bounded. Second, if $W_{S_b}(N) > 0$, the second term can be written as

$$I_{S_b}(N) = W_{S_b}(N) \sum_{i\in S_b} \pi_i \frac{AN}{\lambda_{i,N}}, \tag{S4.8}$$

where

$$\pi_i = \frac{\cos^2\theta_{i,N}}{W_{S_b}(N)}\,, \quad \sum_{i\in S_b} \pi_i = 1 \tag{S4.9}$$

forms the probability mass function. Therefore, the summation term in Eq. S4.8 is a weighted average whose smallest term is $AN/\lambda_{|L|+1,N}$ because the eigenvalues are ordered. Since $\lambda_{|L|+1,N}$, the largest sublinear eigenvalue, grows more slowly than N, this smallest term grows without bound, and therefore so does the weighted average. Below, we denote this expectation by $\langle\cdot\rangle$. Thus the necessary and sufficient condition that $I_{S_b}(N)$ (as well as I_N) is bounded is

$$W_{S_b}(N) \left\langle \frac{N}{\lambda_{i,N}} \right\rangle = O(1)\,. \tag{S4.10}$$

Here and below, $O(N^p)$ denotes a term whose magnitude divided by N^p remains bounded as $N \to \infty$; $O(1)$ corresponds to $p = 0$. Note that due to the definition of the sublinear eigenvalues and finiteness of L, $\langle N/\lambda_{i,N}\rangle^{-1}$ tends to zero in the limit. Thus, $W_{S_b}(N)$ must converge to zero at a rate equal to or faster than $\langle N/\lambda_{i,N}\rangle^{-1}$.

In summary, the necessary and sufficient condition that the information is bounded is that either $W_{S_b}(N) = 0$ or $W_{S_b}(N)$ converges to zero at a rate equal to or faster than $\langle N/\lambda_{i,N}\rangle^{-1}$. The LFI is unbounded if and only if $W_{S_b}(N)$ fails to approach zero rapidly enough to compensate for the growth of $\langle N/\lambda_{i,N}\rangle$, so that their product does not remain bounded as N increases.

Necessary condition Consequently, the necessary condition for the bounded information is that $W_{S_b}(N)$ converges to zero, equivalently $W_L(N)$ converges to one:

$$\lim_{N\to\infty} W_{S_b}(N) = 0 \Longleftrightarrow \lim_{N\to\infty} W_L(N) = 1. \tag{S4.11}$$

Further, one can consider a set that covers the linear eigenvalues and let the angular occupation by this set approach one to satisfy a necessary condition. We consider a positive integer $\kappa(N) \le N$ such that a set of $i = 1, \dots, \kappa(N)$ includes all linear eigenvalues. We define their angular occupation as $W_\kappa(N) = \sum_{i=1}^{\kappa(N)} \cos^2\theta_{i,N}$. For the information to be bounded, it is necessary that $W_\kappa(N)$ converge to one:

$$\lim_{N\to\infty} W_\kappa(N) = 1 \tag{S4.12}$$

The correlations that violate this necessary condition result in unbounded information.

Sufficient conditions An exemplary sufficient condition for the bounded information is obtained as follows. Since $\lambda_{i,N} \ge \lambda_{N,N}$, we obtain the upper bound of $\langle N/\lambda_{i,N}\rangle$ by replacing $\lambda_{i,N}$ ($i = 1, \dots, N-1$) with $\lambda_{N,N}$: $\langle N/\lambda_{i,N}\rangle \le N/\lambda_{N,N}$. By multiplying $W_{S_b}(N) > 0$, we have the upper bound of $I_{S_b}(N)$

$$I_{S_b}(N) = W_{S_b}(N) \cdot \langle AN/\lambda_{i,N}\rangle \le W_{S_b}(N) \cdot (AN/\lambda_{N,N}). \tag{S4.13}$$

Thus, it is sufficient that $W_{S_b}(N)$ approach zero at least as fast as $\lambda_{N,N}/N$. Further, it is also sufficient to find a set containing all sublinear eigenvalues whose total angular occupation approaches zero at least as fast as $\lambda_{N,N}/N$.

Below, we present exemplary covariance matrices that yield bounded and unbounded LFI, assuming specific scaling conditions for the eigenvalues and angular occupation (except for Example 1).

Example 1: Unbounded LFI If the eigenvalues of the covariance matrix are all sublinear functions, we have $W_{S_b} = 1$, which clearly violates the necessary condition. Thus, the LFI for such populations is unbounded. Trial-shuffled covariance matrices provide a simple example. In the infinite-trial limit, shuffling makes the covariance matrix diagonal, so its eigenvalues are the individual-neuron noise variances. As assumed for subsampled populations in text S2, these variances are bounded above by a fixed constant independent of N. Hence, $\lambda_{1,N} = O(1)$, so $\lambda_{1,N}/N \to 0$ and all eigenvalues scale sublinearly.

Example 2: Bounded/unbounded LFI We consider only one sublinear ($i = N$) eigenvalue that has a finite cosine direction ($\cos^2\theta_{N,N} > 0$), while the rest of the sublinear eigenvalues have no angular occupation ($\cos^2\theta_{i,N} = 0$ for $i \neq N$ and $i \in S_b$). The linear eigenvalues occupy the rest of the angular occupation: $W_L = 1 - \cos^2\theta_{N,N}$. Then, for the LFI to be bounded, $W_{S_b} = \cos^2\theta_{N,N}$ must decrease at a rate equal to or faster than $\lambda_{N,N}/N$. If $\cos^2\theta_{N,N} = N^{-\delta}$ and $\lambda_{N,N} \sim N^{\zeta}$ ($\zeta < 1$), the exponent must satisfy $-\delta \leq \zeta - 1$, namely $\delta \geq 1 - \zeta$. Otherwise, if $\delta < 1 - \zeta$, the LFI is unbounded.

Example 3: Bounded/unbounded LFI We consider the first N_c eigenvalues to be linear, and the rest are sublinear eigenvalues given by $\lambda_{i,N} \sim N^{\zeta}$ with $\zeta \leq 0$ ($i = N_c + 1, \dots, N$). Note that $\zeta \leq 0$ is necessary for linearly scaling total variance. We then assign $\cos^2\theta_{i,N} = N^{-\delta}$ for $i = N_c + 1, \dots, N$. The angular occupation by sublinear eigenvalues is

$$W_{S_b} = \sum_{i=N_c+1}^{N} N^{-\delta} = (N - N_c)N^{-\delta} = (1 - N_c/N)N^{1-\delta} \sim N^{1-\delta}, \tag{S4.14}$$

from which we find δ must satisfy $\delta \geq 1$ so that their total angular occupation does not exceed one as N grows. For the LFI to be bounded, W_{S_b} must decay at least as fast as $\langle N/\lambda_{i,N}\rangle^{-1} \sim N^{\zeta-1}$. Thus if $1 - \delta \leq \zeta - 1$, namely $\delta \geq 2 - \zeta$, the information is bounded. Otherwise, if $\delta < 2 - \zeta$, the LFI is unbounded.

As seen in these examples, one can construct the populations with bounded and unbounded LFI by appropriately assigning the angular occupations $\cos^2\theta_{i,N}$ to eigenvectors associated with linear or sublinear eigenvalues. However, the assignment must be done by keeping the sum of the angular occupations one (Eq. S4.2). Here, we summarize its consequences, including the cases when the angular occupations scale with N.

1. Assuming monotonicity, the angular occupation must be either (i) a function of N approaching a nonzero constant equal to or less than one in the large-N limit, or (ii) a decreasing function of N that vanishes to zero in the large-N limit or a constant zero. For the following classification, assume that, for every fixed j, the angular occupation has a limit $c_j \equiv \lim_{N\to\infty} \cos^2\theta_{j,N}$.
2. Let C be the set with the nonzero angular occupations in the large-N limit:

   $$C = \{ j \geq 1 \mid c_j > 0 \}. \tag{S4.15}$$

   The set C may be finite or countably infinite, with $\sum_{i\in C} c_i \leq 1$. For bounded LFI under the finite-L assumption, the necessary condition $W_L(N) \to 1$ implies $C \subseteq L$. Consequently, C is finite and $\sum_{i\in C} c_i = 1$. Some linear eigenvectors may have limiting angular occupation zero, so C may contain fewer eigenvectors than L. The angular occupations of the complement of C ($i \notin C$) are zeros or functions vanishing to zero in the large-N limit.
3. At population size N, only the indices $1, \dots, N$ are present. Let $C_N = C \cap \{1, \dots, N\}$ denote those belonging to C. We define the total angular occupation by the set C as

   $$W_C(N) = \sum_{i\in C_N} \cos^2\theta_{i,N}. \tag{S4.16}$$

Suppose that, in the large-N limit, the components in C_N do not carry all the angular occupation. The remaining components must then carry a nonzero total occupation. More precisely, if $W_C(N) \to w < 1$, then their total occupation, $1 - W_C(N)$, tends to $1 - w > 0$. To illustrate how many small occupations can produce such a nonzero total, consider a group of $m_\delta(N)$ components, each with angular occupation of order $N^{-\delta}$, with the same bounds applying to every component in the group. The group's total occupation is of order $m_\delta(N)N^{-\delta}$. For this group to retain a positive total occupation, $m_\delta(N)$ must grow in proportion to N^δ.

4. In contrast, suppose $W_C(N) \to 1$, so the total angular occupation of the remaining components, $1 - W_C(N)$, tends to zero. For a group with the same scaling as above, the total occupation is of order $m_\delta(N)N^{-\delta}$ and must now vanish. Therefore, unlike in the preceding case, $m_\delta(N)$ must grow more slowly than N^δ. In particular, if the group grows linearly, $m_\delta(N) \propto N$, its total occupation vanishes when $\delta > 1$.

These consequences can be utilized to set a range of scaling parameters for the angular occupations when constructing populations with the bounded and unbounded LFI. For example, for the LFI to be bounded, the nonzero limiting angular occupations must be assigned to linear eigenvalues, and their sum, $W_C(N)$, must converge to one to satisfy the necessary condition (Eq. S4.11). Example 3 applies item #4 by assigning the decreasing angular occupations ($\cos^2\theta_{i,N} = N^{-\delta}$) to the set of sublinear eigenvalues $i = N_c + 1, \ldots, N$, whose number increases linearly. In this case, the decreasing angular occupations must scale with $\delta > 1$, allowing us to set the range of the parameter δ. Note that this is a consequence of the necessary condition for bounded LFI under Eq. S4.2. The LFI is bounded only if $\delta \geq 2 - \zeta$, as derived in Example 3.

Interestingly, under the finite-L assumption of this note, if C is an empty set, namely if all $\cos^2\theta_{i,N}$ are decreasing functions of N that vanish to zero or constant zeros, the LFI is unbounded regardless of the composition of the eigenspectrum. Since the set L is assumed to be finite in this note, the sum of their angular occupations diminishes to zero, which violates the necessary condition (Eq. S4.11) for the bounded LFI. The trial-shuffled covariance matrices in Example 1, for which $L = \varnothing$, also illustrate the angular-occupation behavior described above. In the infinite-trial limit, their eigenvectors can be chosen as the single-neuron axes. For the axis associated with neuron j, the angular occupation is $\cos^2\theta_{j,N} = f_j'(s)^2/|\mathbf{f}_N'(s)|^2$. Since the individual squared signal components remain bounded while the total squared signal magnitude grows proportionally to N, every angular occupation is $O(N^{-1})$ and vanishes. Thus, $C = \varnothing$, consistent with the unbounded LFI established in Example 1.

Finally, the necessary and sufficient condition for bounded LFI derived here assumes linearly growing total noise variance and a finite set L of linearly scaling eigenvalues. These assumptions constrain the covariance matrix at each N, but not how the covariance matrices at different population sizes are related. If the populations form a nested sequence created by successively adding neurons, each covariance matrix is contained in the next, and the LFI cannot decrease with N (text S8). Because the boundedness condition derived here does not require this nested structure, it also applies to more general covariance sequences, including those for which the LFI decreases with N (text S11).

text S5. Scale-invariant eigenspectrum under the linear scaling of the total variance

In this note, we clarify the relationship between the scale invariance of the eigenspectrum, the exponent of the power-law spectrum, and the eigenvalue scaling under the linear scaling of the total variance.

We start by assuming that the eigenspectrum is written in the form:

$$\lambda_{i,N} = c(N) g(x_i), \tag{S5.1}$$

where $c(N)$ is the scaling factor depending on the population size N and $g(x_i)$ is the scale-free structure of the eigenspectrum on the normalized index, $x_i = i/N$.

As discussed in text S2, subsampled populations satisfy linear total-variance scaling:

$$V_N = \sum_{i=1}^{N} \lambda_{i,N} = c(N) \sum_{i=1}^{N} g(x_i) \sim N. \tag{S5.2}$$

Using the integral approximation, the above summation is approximated as

$$c(N) \sum_{i=1}^{N} g(x_i) = N c(N) \sum_{i=1}^{N} g(x_i) \frac{1}{N} \approx N c(N) \int_{1/N}^{1} g(x) \mathrm{d}x. \tag{S5.3}$$

Note that the bounds of the integral are chosen over the domain of the function $g(x)$, which is $[1/N, 1]$. We break the above integral into two parts:

$$\int_{1/N}^{1} g(x) \mathrm{d}x = \int_{1/N}^{x_0} g(x) \mathrm{d}x + \int_{x_0}^{1} g(x) \mathrm{d}x. \tag{S5.4}$$

While the second integral on the right-hand side of the above equation is independent of N, the first integral depends on N.

Suppose that we have the power-law spectrum $g(x) \sim x^{-\mu}$ ($\mu > 0$) at the limit of early indices of the eigenspectrum ($x \ll 1$). Because the eigenspectrum decreases with rank, its slope is negative, implying $\mu > 0$. If $\mu \neq 1$, the above integral is approximated as

$$\begin{aligned} \int_{1/N}^{x_0} g(x) \mathrm{d}x &\sim \int_{1/N}^{x_0} x^{-\mu} \mathrm{d}x \\ &= \left[\frac{1}{1-\mu} x^{1-\mu} \right]_{1/N}^{x_0} = \frac{1}{\mu - 1} \left(N^{\mu-1} - x_0^{1-\mu} \right). \end{aligned} \tag{S5.5}$$

Further, if $\mu > 1$, the above integral, Eq. S5.5, scales as $N^{\mu-1}$, resulting in

$$\int_{1/N}^{1} g(x) \mathrm{d}x \sim N^{\mu-1} \tag{S5.6}$$

From Eqs. S5.3 and S5.6, the total variance scales as $V_N \propto c(N) N^{\mu}$. The constraint of the linearly scaling total variance imposes $c(N) = N^{-\alpha}$ with $\alpha = \mu - 1 > 0$, resulting in the following scale invariance:

$$\lambda_{i,N} = N^{-\alpha} g(x_i). \tag{S5.7}$$

In this case, the eigenvalues at every fixed rank i scale linearly, $\lambda_{i,N} \sim N^{-\alpha} (i/N)^{-\mu} = N^{\mu-\alpha} \sim N$ with $\mu = 1 + \alpha$. These linearly scaling noise components can potentially limit the LFI.

By contrast, if $0 < \mu < 1$, the above integral, Eq. S5.5, converges to a positive constant. Then, the total variance scales as $V_N \sim N c(N)$, making $c(N)$ constant under the linear-scaling total variance constraint. In this case, we have the following scale-invariant eigenspectrum:

$$\lambda_{i,N} \sim g(x_i), \tag{S5.8}$$

which can also be written in the form of Eq. S5.7 using $\alpha = 0$. The eigenvalues at the limit of early indices scale sublinearly, $\lambda_{i,N} \sim N^{\mu}$ for a fixed $i (\ll N)$. Since the eigenspectrum is constructed

in descending order, the later eigenvalues cannot exceed the earlier eigenvalues, from which we conclude that all of the eigenvalues are sublinear functions of N if $\alpha = 0$. Without the presence of the linear scaling eigenvalues, the LFI cannot be limited (text S1). This result implies that when neural populations show scale-invariant eigenspectra with $\alpha = 0$ in their noise covariance, their LFI is unbounded as long as the signal magnitude ($|\mathbf{f}'_N|^2$) scales linearly with N.

Finally, the critical exponent $\mu = 1$ results in $V_N \sim N c(N) \log N$, resulting in $c(N) \sim (\log N)^{-1}$. It is a decreasing function of N for $N > 1$. The eigenvalues at early indices scale as $\lambda_{i,N} \sim N / \log N$.

In summary, if we observe a scale-invariant eigenspectrum with an exponent $\alpha > 0$ for large N, the exponent μ of the power-law scaling must be $1 + \alpha$ in the limit of early indices, and the leading eigenvalues scale linearly. By contrast, if we observe a scale-invariant eigenspectrum with an exponent $\alpha = 0$, the exponent of the power-law scaling μ must be less than one, and all eigenvalues scale sublinearly. We note that the same constraints are obtained by requiring the finite-N empirical eigenvalue mean to approach a finite, nonzero limit (text S6).

text S6. Scale invariance and LFI in terms of eigenvalue distributions

In this note, we show that the relation between the scaling parameter α and the power-law exponent μ of the eigenvalue spectrum under the linear total variance is obtained by requiring the mean eigenvalue to have a finite, positive limit as N increases.

Let $\hat{F}_N(\lambda) = \frac{1}{N}\sum_{i=1}^{N} \mathbf{1}_{\lambda_i \le \lambda}$ be an empirical cumulative distribution function (CDF) of the eigenvalues. Here, $\mathbf{1}_{\lambda_i \le \lambda}$ is an indicator function that takes one if the condition of the subscript is satisfied. Let $x_i = i/N$ for $i = 1, \ldots, N$ be the normalized index of the eigenvalue λ_i. Since we ordered the eigenvalues in descending order, it is related to the empirical CDF as

$$x_i = 1 - \hat{F}_N(\lambda_i) + \frac{1}{N}. \tag{S6.1}$$

For simplicity, we assume that no two eigenvalues are exactly equal. We approximate $\hat{F}_N(\lambda_i)$ by a smooth CDF $F_N(\lambda)$ for each finite N, where λ is a nonnegative real value. Then, the eigenvalue spectrum and the CDF $F_N(\lambda)$ of the eigenvalues are related as follows.

$$1 - x \simeq F_N(\lambda), \tag{S6.2}$$

where we introduced $x \in (0, 1]$. Similarly, we approximate the empirical density of the eigenvalues $\hat{\rho}_N(\lambda) = \frac{1}{N}\sum_{i=1}^{N} \delta(\lambda - \lambda_i)$ by a continuous density function $\rho_N(\lambda) = \frac{dF_N(\lambda)}{d\lambda}$.

Thus, under the scaling-invariant eigenspectrum satisfying

$$\lambda(x) = N^{-\alpha} g(x), \tag{S6.3}$$

the eigenvalue density is related to $g(x)$ via

$$\rho_N(\lambda) = \frac{dF_N(\lambda)}{d\lambda} \simeq -\frac{dx}{d\lambda} = -\left(\frac{d\lambda}{dx}\right)^{-1} = -N^{\alpha}\left(\frac{dg(x)}{dx}\right)^{-1}. \tag{S6.4}$$

In the following, we assume the power-law for the eigenvalue spectrum at low indices: $g(x) = bx^{-\mu}$ for $x \ll 1$. Then, if $\mu \neq 1$, the eigenvalue density follows the power-law with exponent $1 + \frac{1}{\mu}$ at $x \ll 1$:

$$\rho_N(\lambda) \propto \lambda^{-\left(1+\frac{1}{\mu}\right)} \tag{S6.5}$$

This is because, from $\lambda = N^{-\alpha} b x^{-\mu}$, we have

$$x = g^{-1}(N^{\alpha}\lambda) = b^{\frac{1}{\mu}}(N^{\alpha}\lambda)^{-\frac{1}{\mu}}, \tag{S6.6}$$

hence

$$\frac{dg(x)}{dx} = -b\mu x^{-\mu-1} = -b^{-\frac{1}{\mu}}\mu(N^{\alpha}\lambda)^{1+\frac{1}{\mu}}. \tag{S6.7}$$

The constraint of the linear scaling of the total variance or the sum of eigenvalues translates into the condition that the mean eigenvalue approaches a finite positive value. More specifically, assuming that the smoothing error in the mean vanishes as N increases, we note that the mean of the sampled eigenvalues is related to the eigenvalue spectrum as follows:

$$\lim_{N\to\infty}\int \hat{\rho}_N(\lambda)\lambda\, d\lambda = \lim_{N\to\infty}\int_{\lambda_N}^{\lambda_1} \rho_N(\lambda)\lambda\, d\lambda = \lim_{N\to\infty}\int_{\lambda_N}^{\lambda_1}\left(-\lambda\frac{dx}{d\lambda}\right)d\lambda = \lim_{N\to\infty}\int_{1/N}^{1}\lambda(x)\,dx. \tag{S6.8}$$

For $\mu \neq 1$ and $\alpha > 0$, the right-hand side of the above equation is given as

$$\begin{aligned}
\int_{1/N}^{1}\lambda(x)\,dx &= \int_{1/N}^{x_0} N^{-\alpha} g(x) dx + \int_{x_0}^{1} N^{-\alpha} g(x)\,dx \\
&= \int_{1/N}^{x_0} N^{-\alpha} b x^{-\mu}\,dx + CN^{-\alpha} \\
&= \frac{b}{1-\mu}[x_0^{1-\mu}N^{-\alpha} - N^{\mu-1-\alpha}] + CN^{-\alpha},
\end{aligned} \tag{S6.9}$$

where C is a constant value. Note that $N^{-\alpha} \to 0$ in the large-N limit for $\alpha > 0$. Thus, for the above equation to approach a finite, nonzero value, we need to impose $\mu - 1 - \alpha = 0$, or $\mu = 1 + \alpha$. In this case, $\mu > 1$, which makes the integral positive and finite.

For $\mu \neq 1$ and $\alpha = 0$, the right-hand side of the above equation is given as

$$\begin{aligned}\int_{1/N}^{1} \lambda(x)\,dx &= \int_{1/N}^{x_0} g(x)\,dx + \int_{x_0}^{1} g(x)dx \\ &= \int_{1/N}^{x_0} bx^{-\mu}\,dx + C \\ &= \frac{b}{1-\mu}[x_0^{1-\mu} - N^{\mu-1}] + C.\end{aligned} \tag{S6.10}$$

For the above equation to have a finite limit, μ must satisfy $\mu < 1$ so that $N^{\mu-1} \to 0$ in the large-N limit. These results are consistent with the scaling relations obtained under the linear scaling of the total variance in text S5 (The case with $\mu = 1$ is treated separately there).

Finally, we also note that the LFI can be computed using the finite-N distributions of $\lambda_{i,N}$ and $\cos^2\theta_{i,N}$ as follows, where $c = \cos^2\theta$ and $p_N(\lambda, c)$ is their joint distribution:

$$\begin{aligned}I_N &= |\mathbf{f}'_N(s)|^2 \sum_{i=1}^{N} \frac{\cos^2\theta_{i,N}}{\lambda_{i,N}} = AN^2 \frac{1}{N}\sum_{i=1}^{N} \frac{\cos^2\theta_{i,N}}{\lambda_{i,N}} \\ &\approx AN^2 \int p_N(\lambda, c)\frac{c}{\lambda}\,d\lambda\,dc \\ &= AN^2 \int \rho_N(\lambda)p_N(c|\lambda)\frac{c}{\lambda}\,d\lambda\,dc \\ &= AN^2 \int d\lambda\rho_N(\lambda)\frac{1}{\lambda}\int p_N(c|\lambda)c\,dc\end{aligned} \tag{S6.11}$$

Here we note that the power-law relation between the eigenvalue and angular occupation (Eq. 7 or Eq. S3.1) specifies the conditional expectation of the angular occupation. Then, following the definition of $\Lambda(\gamma)$ in text S3, we obtain

$$\begin{aligned}I_N &\approx AN^2 \int d\lambda\rho_N(\lambda)\frac{1}{\lambda}\frac{\lambda^\gamma}{N\int\rho_N(\lambda)\lambda^\gamma d\lambda} \\ &= AN\frac{N\int\rho_N(\lambda)\lambda^{\gamma-1}d\lambda}{N\int\rho_N(\lambda)\lambda^\gamma d\lambda} \\ &\approx AN\frac{\Lambda(\gamma-1)}{\Lambda(\gamma)},\end{aligned} \tag{S6.12}$$

which recovers, within the continuum approximation, Eq. S3.17.

text S7. Subspace analysis by partial least squares may incorrectly report saturated LFI

In this note, we show that analyzing LFI using a dimension-reduction method, known as the Partial Least Squares (PLS) discriminant analysis, can result in an incorrect conclusion about its boundedness, while our method correctly predicts its behavior in the large-N limit.

The PLS discriminant analysis identifies directions within a subspace of measurements that most effectively discriminate between two stimuli. The study by Rumyantsev et al. (*16*) applied the PLS analysis to distill the high-dimensional neural space into a latent space of five dimensions obtained from stimulus-informative PLS directions. They discovered that the noise eigenvector within this reduced space most aligned with the signal direction had an eigenvalue that scaled linearly with the population size across five examined mice, and concluded that these and other eigenvalues that increase with population size were responsible for the saturated behavior of the LFI, denoted as d' in their analysis. However, the PLS directions are not generally principal eigenvectors of the full covariance, and the covariance eigenvalues within the PLS subspace need not be the leading eigenvalues of the full covariance. Thus, restricting the LFI to the PLS subspace may miss collective contributions from signal components distributed across many full-space noise eigenvectors, including those with sublinearly scaling eigenvalues. Consequently, the full-space LFI can be unbounded even when the reduced-space LFI appears bounded.

To test this hypothesis, we simulated neural populations exhibiting unbounded LFI theoretically. For this goal, for each population of size N, we constructed N eigenvalues from the eigenspectrum given by Eqs. 5 and 6 with the parameters derived from mouse TX42 whose estimated scaling parameter was $\alpha = 0.164 \pm 0.002$. To define angular occupations, we employed the relation between the angular occupation and eigenvalues given by Eq. 7 with an exponent $\gamma = 1$, which results in unbounded LFI for any $\alpha > 0$ (text S3).

More specifically, we constructed the covariance matrix $\mathbf{\Sigma}_N$ by associating random orthogonal vectors with the eigenvalues obtained from Eqs. 5 and 6. To construct $\mathbf{f}_N(s)$ and $\mathbf{f}_N(s + \delta s)$, we assumed that the activity means are equal to the variance (i.e., the diagonal of $\mathbf{\Sigma}_N$). We then constructed $\mathbf{f}_N(s + \delta s) = \mathbf{f}_N(s) + \mathbf{f}'_N(s)\delta s$, where $\mathbf{f}'_N(s) = |\mathbf{f}'_N(s)| \sum_{i=1}^{N} \cos\theta_{i,N} \mathbf{u}_{i,N}$ is constructed using the $\cos^2\theta_{i,N}$ that comes from the power-law relation given by Eq. 7 using $\gamma = 1$. We then sampled neural activities from the Gaussian distribution $\mathcal{N}(\mathbf{f}_N(s), \mathbf{\Sigma}_N)$ and $\mathcal{N}(\mathbf{f}_N(s+\delta s), \mathbf{\Sigma}_N)$. The sample covariance matrix and signal for a subpopulation of size N were constructed from 2600 samples (trials). The eigenspectra and angular occupations for subpopulation size N were obtained by averaging the results from 20 independently generated distributions. To compare the PLS and scaling-based analyses under the same finite-sample conditions, we used raw sample eigenvalues and angular occupations without bias correction.

The leading eigenvalues of the simulated dataset scale linearly as expected for $\alpha > 0$ (fig. S9A). As expected, the data show both the collapse of the eigenvalue spectrum by the scaling and the power-law relation between eigenvalues and angular occupations (fig. S9B,C). The scaling parameter and the slope were estimated as $\alpha = 0.203 \pm 0.0001$ and $\gamma = 1.009 \pm 0.011$. By applying the PLS method to the unbounded data, we find that the 10 eigenvalues extracted by the PLS method all scale linearly with population size (fig. S9D), and only partially recover the scaling behavior of the full system (fig. S9E,F).

Figure S9G shows the estimated LFI based on the PLS and our scaling-based method. The estimate of the LFI based on the PLS analysis (red) shows the saturated behavior, which follows the curve predicted by the differential correlations (dotted line). To the contrary, our scaling-based method (green) with estimated α and γ correctly predicts that the LFI of the population is unbounded (fig. S9H). We note that the large eigenvalues from finite samples are biased upwards, compared to the original values (*43*). While this results in the overestimation of α, using the estimated α is sufficient to conclude the unboundedness of the LFI because the critical γ_c is a decreasing function of α. For comparison, we provided the bias-corrected LFI estimate (*17*, *25*), which

estimates the LFI precisely. However, we note that the method cannot predict the behavior of LFI beyond the number set by the number of trials and neurons.

To further investigate the behavior of the PLS analysis, we simulated neural populations exhibiting unbounded LFI, assuming that the eigenvalues follow the scale invariance given by Eq. 5 with $\alpha = 0$. We proved that the LFI of subsampled populations is unbounded under this condition (text S5). All the eigenvalues, including the leading eigenvalues, scale sublinearly. We then sampled eigenvalues from the same modified $\alpha = 0$ eigenspectrum with the remaining parameters derived from mouse TX42. We set the leading-edge exponent to $\mu = 0.5$ and the angular-occupation exponent to $\gamma = 1$. Thus, μ was chosen independently rather than constrained to equal $1 + \alpha$ in this simulation.

As expected, the leading eigenvalues of the sampled data scaled sublinearly (fig. S10A). The eigenvalue spectrum in the normalized indices was consistent with $\alpha = 0$ under the scaling-collapse fit (fig. S10B). The fitted exponent γ was $\gamma = 0.968 \pm 0.016$ (fig. S10C), which is close to 1, but the error was larger than the previous example, possibly due to the lack of a large magnitude of $\lambda_{i,N}$ because all eigenvalues scale sublinearly.

When we apply the PLS method to these data, we find that the largest eigenvalue scales closer to a linear line than the original eigenvalues (fig. S10D), despite the fact that there are no linearly scaling eigenvalues in the original dataset. Similarly to the previous example, the method did not fully recover the scaling behavior present in the full system. Accordingly, the estimate of the LFI based on the PLS analysis for these unbounded data shows again the saturated behavior (fig. S10G). Although our scaling-based analysis overestimated the magnitude of the LFI for $\alpha = 0$, it correctly predicted its unbounded behavior (fig. S10H).

text S8. LFI of the subsampled populations is a non-decreasing function of population size

In this note, we derive a recurrence relation for the LFI of subsampled populations. This relation clarifies how the LFI changes when a neuron is added to a subpopulation, demonstrating that the LFI of subsampled populations is a non-decreasing function of population size.

When we increase the number of neurons by adding a neuron to an existing subpopulation of size N, the covariance of the larger population of size $N+1$, $\mathbf{\Sigma}_{N+1}$, contains the covariance of the population of size N, $\mathbf{\Sigma}_N$. Let Σ_{N+1} be the variance of the $(N+1)$-th neuron and $\mathbf{c}_N$ be the covariance between the existing N neurons and the $(N+1)$th neuron. The covariance matrix of the $N+1$ neurons is written as

$$\mathbf{\Sigma}_{N+1} = \begin{bmatrix} \mathbf{\Sigma}_N & \mathbf{c}_N \\ \mathbf{c}_N^{\mathrm{T}} & \Sigma_{N+1} \end{bmatrix}. \tag{S8.1}$$

The determinant of the sub-matrix $\mathbf{\Sigma}_N$ is the leading principal minor of order N of the matrix $\mathbf{\Sigma}_{N+1}$. Further, the determinants of $\Sigma_1, \mathbf{\Sigma}_2, \ldots, \mathbf{\Sigma}_N, \mathbf{\Sigma}_{N+1}$ constitute the leading principal minors of $\mathbf{\Sigma}_{N+1}$. Sylvester's criterion states that $\mathbf{\Sigma}_{N+1}$ is positive definite if and only if all of the leading principal minors are positive.

From the block determinant formula, we have

$$\det \mathbf{\Sigma}_{N+1} = \det \mathbf{\Sigma}_N \left(\Sigma_{N+1} - \mathbf{c}_N^{\mathrm{T}} \mathbf{\Sigma}_N^{-1} \mathbf{c}_N \right) \tag{S8.2}$$

Given that $\mathbf{\Sigma}_N$ is positive definite, we have $\det \mathbf{\Sigma}_N > 0$. Then, $\mathbf{\Sigma}_{N+1}$ is positive definite if and only if

$$\omega_{N+1} \equiv \Sigma_{N+1} - \mathbf{c}_N^{\mathrm{T}} \mathbf{\Sigma}_N^{-1} \mathbf{c}_N > 0 \tag{S8.3}$$

Starting from a single neuron with a positive variance Σ_1, Sylvester's criterion is satisfied if we construct the covariance matrix recursively while satisfying $\omega_{N+1} > 0$. Indeed, this equation must be satisfied for a subsampled population of any size because the covariance of the subsampled population is positive definite.

We now consider the relation between the LFI of the population with size N and $N+1$. First, we calculate the inverse of the covariance matrix. Using the following formula for the inversion of a block matrix

$$\begin{bmatrix} \mathbf{A} & \mathbf{B} \\ \mathbf{C} & \mathbf{D} \end{bmatrix}^{-1} = \begin{bmatrix} \mathbf{A}^{-1} + \mathbf{A}^{-1}\mathbf{B}\left(\mathbf{D} - \mathbf{C}\mathbf{A}^{-1}\mathbf{B}\right)^{-1}\mathbf{C}\mathbf{A}^{-1} & -\mathbf{A}^{-1}\mathbf{B}\left(\mathbf{D} - \mathbf{C}\mathbf{A}^{-1}\mathbf{B}\right)^{-1} \\ -\left(\mathbf{D} - \mathbf{C}\mathbf{A}^{-1}\mathbf{B}\right)^{-1}\mathbf{C}\mathbf{A}^{-1} & \left(\mathbf{D} - \mathbf{C}\mathbf{A}^{-1}\mathbf{B}\right)^{-1} \end{bmatrix}, \tag{S8.4}$$

we obtain

$$\begin{aligned} \mathbf{\Sigma}_{N+1}^{-1} &= \begin{bmatrix} \mathbf{\Sigma}_N & \mathbf{c}_N \\ \mathbf{c}_N^{\mathrm{T}} & \Sigma_{N+1} \end{bmatrix}^{-1} \\ &= \begin{bmatrix} \mathbf{\Sigma}_N^{-1} + \mathbf{\Sigma}_N^{-1}\mathbf{c}_N \left(\Sigma_{N+1} - \mathbf{c}_N^{\mathrm{T}}\mathbf{\Sigma}_N^{-1}\mathbf{c}_N\right)^{-1} \mathbf{c}_N^{\mathrm{T}}\mathbf{\Sigma}_N^{-1} & -\mathbf{\Sigma}_N^{-1}\mathbf{c}_N \left(\Sigma_{N+1} - \mathbf{c}_N^{\mathrm{T}}\mathbf{\Sigma}_N^{-1}\mathbf{c}_N\right)^{-1} \\ -\left(\Sigma_{N+1} - \mathbf{c}_N^{\mathrm{T}}\mathbf{\Sigma}_N^{-1}\mathbf{c}_N\right)^{-1} \mathbf{c}_N^{\mathrm{T}}\mathbf{\Sigma}_N^{-1} & \left(\Sigma_{N+1} - \mathbf{c}_N^{\mathrm{T}}\mathbf{\Sigma}_N^{-1}\mathbf{c}_N\right)^{-1} \end{bmatrix}. \end{aligned} \tag{S8.5}$$

Using ω_{N+1} and

$$\mathbf{b}_N \equiv \mathbf{\Sigma}_N^{-1}\mathbf{c}_N, \tag{S8.6}$$

the inverse of the covariance matrix is written as

$$\mathbf{\Sigma}_{N+1}^{-1} = \begin{bmatrix} \mathbf{\Sigma}_N^{-1} + \omega_{N+1}^{-1}\mathbf{b}_N\mathbf{b}_N^{\mathrm{T}} & -\omega_{N+1}^{-1}\mathbf{b}_N \\ -\omega_{N+1}^{-1}\mathbf{b}_N^{\mathrm{T}} & \omega_{N+1}^{-1} \end{bmatrix}. \tag{S8.7}$$

Similarly to the covariance matrix, we separate $\mathbf{f}'_{N+1}(s)$ as

$$\mathbf{f}'_{N+1}(s) = \begin{bmatrix} \mathbf{f}'_N(s) \\ f'_{N+1}(s) \end{bmatrix}. \tag{S8.8}$$

With this, the LFI is obtained as

$$
\begin{aligned}
I_{N+1} &= \mathbf{f}'_{N+1}(s)^{\mathrm{T}}\boldsymbol{\Sigma}_{N+1}^{-1}\mathbf{f}'_{N+1}(s) \\
&= \mathbf{f}'_N(s)^{\mathrm{T}}\boldsymbol{\Sigma}_N^{-1}\mathbf{f}'_N(s) + \omega_{N+1}^{-1}\mathbf{f}'_N(s)^{\mathrm{T}}\mathbf{b}_N\mathbf{b}_N^{\mathrm{T}}\mathbf{f}'_N(s) - \omega_{N+1}^{-1}\mathbf{f}'_N(s)^{\mathrm{T}}\mathbf{b}_N f'_{N+1}(s) - \omega_{N+1}^{-1} f'_{N+1}(s)\mathbf{b}_N^{\mathrm{T}}\mathbf{f}'_N(s) + \omega_{N+1}^{-1} f'_{N+1}(s)^2 \\
&= \mathbf{f}'_N(s)^{\mathrm{T}}\boldsymbol{\Sigma}_N^{-1}\mathbf{f}'_N(s) + \omega_{N+1}^{-1}\left(\mathbf{f}'_N(s)^{\mathrm{T}}\mathbf{b}_N - f'_{N+1}(s)\right)^2
\end{aligned}
\tag{S8.9}
$$

from which we have

$$
I_{N+1} = I_N + \omega_{N+1}^{-1}\left(\mathbf{f}'_N(s)^{\mathrm{T}}\boldsymbol{\Sigma}_N^{-1}\mathbf{c}_N - f'_{N+1}(s)\right)^2. \tag{S8.10}
$$

Since $\omega_{N+1} > 0$, the second term of the above equation is nonnegative. Hence, we obtain

$$
I_{N+1} \geq I_N, \tag{S8.11}
$$

where the equality holds for $\mathbf{f}'_N(s)^{\mathrm{T}}\boldsymbol{\Sigma}_N^{-1}\mathbf{c}_N = f'_{N+1}(s)$. This proves that the LFI of the subsampled population is a non-decreasing function of N.

Eq. S8.10 provides insights into how the information increases or does not increase by adding a neuron. First, if $\mathbf{c}_N = \mathbf{0}$, namely, if an added neuron is independent of the other N neurons, the LFI increases by $\Sigma_{N+1}^{-1} f'^2_{N+1}$. Further, if $f'_{N+1} = 0$, namely, even if we add a neuron that is not sensitive to a stimulus, the LFI increases by $\omega_{N+1}^{-1}\left(\mathbf{f}'_N(s)^{\mathrm{T}}\boldsymbol{\Sigma}_N^{-1}\mathbf{c}_N\right)^2$. Finally, the LFI does not increase if $\mathbf{f}'^{\mathrm{T}}_N\boldsymbol{\Sigma}_N^{-1}\mathbf{c}_N = f'_{N+1}$.

Note that the LFI can be interpreted as an infinitesimal value of the squared standardized mean difference along the direction that discriminates the distributions of the neural activity responding to two close stimulus conditions s and $s + ds$. If we have two multivariate Gaussian distributions with means $\mathbf{f}_N(s)$ and $\mathbf{f}_N(s + ds)$ and the same covariance matrix $\boldsymbol{\Sigma}_N$, linear discriminant analysis shows that the direction $\mathbf{w}_N = \boldsymbol{\Sigma}_N^{-1}\delta\mathbf{f}_N(s)$, where $\delta\mathbf{f}_N(s) = \mathbf{f}_N(s + ds) - \mathbf{f}_N(s)$, best discriminates the stimuli. One can project the data on this line by $\mathbf{w}_N^{\mathrm{T}}\mathbf{x}$, resulting in univariate distributions with $\mathcal{N}(\mathbf{w}_N^{\mathrm{T}}\mathbf{f}_N(s), \mathbf{w}_N^{\mathrm{T}}\boldsymbol{\Sigma}_N\mathbf{w}_N)$ or $\mathcal{N}(\mathbf{w}_N^{\mathrm{T}}\mathbf{f}_N(s + ds), \mathbf{w}_N^{\mathrm{T}}\boldsymbol{\Sigma}_N\mathbf{w}_N)$, depending on the stimuli. The separability of these two distributions is quantified by the standardized mean difference,

$$
\frac{\mathbf{w}_N^{\mathrm{T}}\mathbf{f}_N(s + ds) - \mathbf{w}_N^{\mathrm{T}}\mathbf{f}_N(s)}{\sqrt{\mathbf{w}_N^{\mathrm{T}}\boldsymbol{\Sigma}_N\mathbf{w}_N}}, \tag{S8.12}
$$

and its square is given as

$$
\frac{\left(\mathbf{w}_N^{\mathrm{T}}\delta\mathbf{f}_N(s)\right)^2}{\mathbf{w}_N^{\mathrm{T}}\boldsymbol{\Sigma}_N\mathbf{w}_N} = \frac{\left(\delta\mathbf{f}_N(s)^{\mathrm{T}}\boldsymbol{\Sigma}_N^{-1}\delta\mathbf{f}_N(s)\right)^2}{\delta\mathbf{f}_N(s)^{\mathrm{T}}\boldsymbol{\Sigma}_N^{-1}\boldsymbol{\Sigma}_N\boldsymbol{\Sigma}_N^{-1}\delta\mathbf{f}_N(s)} = \delta\mathbf{f}_N(s)^{\mathrm{T}}\boldsymbol{\Sigma}_N^{-1}\delta\mathbf{f}_N(s) \simeq I_N(ds)^2. \tag{S8.13}
$$

Thus, $I_N = I_{N+1}$, namely $\mathbf{f}'_N(s)^{\mathrm{T}}\boldsymbol{\Sigma}_N^{-1}\mathbf{c}_N = f'_{N+1}$, is the condition under which adding the neuron does not improve the optimal linear discriminability between the two stimuli.

text S9. Noise of bounded subsampled populations contains differential correlations

In this note, we prove that the noise covariance of subsampled populations inducing saturating LFI and satisfying $I_N < I_\infty$ at every finite N can be decomposed into a covariance that induces unbounded LFI and differential correlations. This demonstrates that the boundedness of LFI under subsampling conditions is directly linked to the presence of differential correlations.

Given an (invertible) covariance matrix $\mathbf{\Sigma}_N^0$ whose LFI $I_0 = \mathbf{f}_N'(s)^{\mathrm{T}}(\mathbf{\Sigma}_N^0)^{-1}\mathbf{f}_N'(s)$ diverges as N goes to infinity, Moreno-Bote et al. (*22*) proved that the following specific form of correlations always limits the information:

$$\mathbf{\Sigma}_N = \mathbf{\Sigma}_N^0 + \epsilon \mathbf{f}_N'(s)\mathbf{f}_N'(s)^{\mathrm{T}}, \tag{S9.1}$$

where $\epsilon \mathbf{f}_N'(s)\mathbf{f}_N'(s)^{\mathrm{T}}$ represents differential correlations, with ϵ being a (positive) constant. It can be confirmed as follows. Applying the Sherman–Morrison formula, the inverse of $\mathbf{\Sigma}_N$ is given as

$$\mathbf{\Sigma}_N^{-1} = (\mathbf{\Sigma}_N^0)^{-1} - \frac{\epsilon}{1+\epsilon I_0}(\mathbf{\Sigma}_N^0)^{-1}\mathbf{f}_N'(s)\mathbf{f}_N'(s)^{\mathrm{T}}(\mathbf{\Sigma}_N^0)^{-1}. \tag{S9.2}$$

Consequently, the LFI $I_N = \mathbf{f}_N'(s)^{\mathrm{T}}\mathbf{\Sigma}_N^{-1}\mathbf{f}_N'(s)$ is given by

$$I_N = \frac{I_0}{1+\epsilon I_0}. \tag{S9.3}$$

Given that $I_0(N) \to \infty$, the above equation is a saturating function that converges to $1/\epsilon$ as N goes to infinity. Thus, the differential correlations limit the information.

It was further demonstrated that the differential correlations are special because adding an outer product of any other direction to $\mathbf{\Sigma}_N^0$ cannot limit the information. Therefore, they proposed that differential correlations are the only information-limiting correlations. The intuition behind this is that the noise structure $\epsilon \mathbf{f}_N'(s)\mathbf{f}_N'(s)^{\mathrm{T}}$ aligns with the direction of a signal change. No linear decoder can use such a structure to improve estimation accuracy. However, because the proof considers only rank-one additions to $\mathbf{\Sigma}_N^0$, it does not exclude information limitation caused by higher-rank additions or by other covariance transformations, such as rotations of $\mathbf{\Sigma}_N^0$.

Here, we remark that it is impossible to directly test for the presence of differential correlations by fitting Eq. S9.3 to empirical LFI from finite-size populations because I_0 is an increasing, unbounded function whose form is unspecified a priori, and ϵ is unknown. Namely, because I_0 is determined by the eigenvalues of $\mathbf{\Sigma}_N^0$ and the corresponding angular occupations (text S4), its scaling depends on the scaling of these quantities. Consequently, Eq. S9.3 can describe any increasing and saturating function of N that remains below the unknown limiting value $1/\epsilon$. Thus, fitting this equation to the empirical LFI cannot, by itself, prove or disprove the presence of differential correlations.

We now examine whether the covariance matrix of subsampled populations resulting in saturation of the LFI contains differential correlations. We start with the assumptions that the covariance matrix $\mathbf{\Sigma}_N$ is positive definite for any finite N and that $I_N = \mathbf{f}_N'(s)^{\mathrm{T}}\mathbf{\Sigma}_N^{-1}\mathbf{f}_N'(s)$ saturates as $N \to \infty$: $I_N \to I_\infty$, where I_∞ is a finite value. To test for the presence of differential correlations, we ask whether

$$\mathbf{\Sigma}_N^0 = \mathbf{\Sigma}_N - \epsilon \mathbf{f}_N'(s)\mathbf{f}_N'(s)^{\mathrm{T}} \tag{S9.4}$$

is a positive definite (and therefore invertible) covariance matrix. If $\mathbf{\Sigma}_N^0$ is positive definite, the Sherman–Morrison formula gives

$$I_0 = \mathbf{f}_N'(s)^{\mathrm{T}}(\mathbf{\Sigma}_N^0)^{-1}\mathbf{f}_N'(s) = \frac{I_N}{1-\epsilon I_N}, \tag{S9.5}$$

or equivalently,

$$I_N = \frac{I_0}{1+\epsilon I_0}. \tag{S9.6}$$

This is precisely the form derived for differential correlations (Eq. S9.3). Choosing $\epsilon = 1/I_\infty$ yields the limiting value $I_\infty = 1/\epsilon$. Moreover, I_0 diverges as $I_N \to I_\infty = 1/\epsilon$, showing that the first term in

the covariance decomposition (Eq. S9.1) indeed has unbounded LFI. Thus, if $\boldsymbol{\Sigma}_N^0 = \boldsymbol{\Sigma}_N - \epsilon \mathbf{f}_N'(s)\mathbf{f}_N'(s)^{\mathrm{T}}$ is positive definite for $\epsilon = 1/I_\infty$, then $\boldsymbol{\Sigma}_N$ admits a decomposition into an unbounded covariance matrix and a differential-correlation term.

We first note the necessity of the strict inequality $I_N < I_\infty$ for the positive definiteness of $\boldsymbol{\Sigma}_N^0$. If $\boldsymbol{\Sigma}_N^0$ is positive definite, then I_0 given by Eq. S9.5 must be finite and nonnegative, which requires $1 - \epsilon I_N > 0$. With $\epsilon = 1/I_\infty$, this condition is equivalent to $I_N < I_\infty$. The monotonicity result for subsampled populations gives $I_{N+1} \geq I_N$ (text S8); hence a saturating subsampled LFI satisfies $I_N \leq I_\infty$ for every finite N. The equality case is degenerate: if $I_N = I_\infty$ for some finite N, monotonicity implies $I_k = I_\infty$ for all $k \geq N$, and $\boldsymbol{\Sigma}_N^0$ is singular because $1 - \epsilon I_N = 0$. We therefore restrict our analysis to the nondegenerate saturating case in which $I_N < I_\infty$ for every finite N.

The remaining task is to prove the converse direction, namely that this strict inequality is sufficient for positive definiteness of $\boldsymbol{\Sigma}_N^0$. We prove this below by exploiting the nested structure of subsampled covariance matrices.

Given a positive definite covariance matrix $\boldsymbol{\Sigma}_{N+1}$, we ask if

$$\boldsymbol{\Sigma}_{N+1}^0 = \boldsymbol{\Sigma}_{N+1} - \epsilon \mathbf{f}_{N+1}'(s)\mathbf{f}_{N+1}'(s)^{\mathrm{T}} \tag{S9.7}$$

is positive definite or not. If so, $\boldsymbol{\Sigma}_{N+1}$ can be decomposed into a covariance matrix and differential correlations. Since we consider an incremental increase in the population size from N to $N+1$, $\boldsymbol{\Sigma}_{N+1}^0$ can be written using Eq. S8.1 as

$$\boldsymbol{\Sigma}_{N+1}^0 = \begin{bmatrix} \boldsymbol{\Sigma}_N - \epsilon \mathbf{f}_N'(s)\mathbf{f}_N'(s)^{\mathrm{T}} & \mathbf{c}_N - \epsilon \mathbf{f}_N'(s) f_{N+1}'(s) \\ \mathbf{c}_N^{\mathrm{T}} - \epsilon f_{N+1}'(s)\mathbf{f}_N'(s)^{\mathrm{T}} & \Sigma_{N+1} - \epsilon f_{N+1}'(s)^2 \end{bmatrix}. \tag{S9.8}$$

Note that the upper left block matrix is $\boldsymbol{\Sigma}_N^0$ (Eq. S9.4). Hence, by applying the block determinant formula, the determinant of $\boldsymbol{\Sigma}_{N+1}^0$ is given by

$$\det \boldsymbol{\Sigma}_{N+1}^0 = \det \boldsymbol{\Sigma}_N^0 \left\{ \Sigma_{N+1} - \epsilon f_{N+1}'(s)^2 - \left(\mathbf{c}_N - \epsilon f_{N+1}'(s)\mathbf{f}_N'(s)\right)^{\mathrm{T}} \left(\boldsymbol{\Sigma}_N^0\right)^{-1} \left(\mathbf{c}_N - \epsilon f_{N+1}'(s)\mathbf{f}_N'(s)\right) \right\}. \tag{S9.9}$$

From this equation, the ratio between the determinant of $\boldsymbol{\Sigma}_{N+1}^0$ and $\boldsymbol{\Sigma}_N^0$ is given as

$$\begin{aligned} \frac{\det \boldsymbol{\Sigma}_{N+1}^0}{\det \boldsymbol{\Sigma}_N^0} &= \Sigma_{N+1} - \epsilon f_{N+1}'(s)^2 - \left(\mathbf{c}_N - \epsilon f_{N+1}'(s)\mathbf{f}_N'(s)\right)^{\mathrm{T}} \left(\boldsymbol{\Sigma}_N^0\right)^{-1} \left(\mathbf{c}_N - \epsilon f_{N+1}'(s)\mathbf{f}_N'(s)\right) \\ &= \Sigma_{N+1} - \epsilon f_{N+1}'(s)^2 \\ &\quad - \mathbf{c}_N^{\mathrm{T}} \left(\boldsymbol{\Sigma}_N^0\right)^{-1} \mathbf{c}_N + 2\mathbf{c}_N^{\mathrm{T}} \left(\boldsymbol{\Sigma}_N^0\right)^{-1} \epsilon f_{N+1}'(s)\mathbf{f}_N'(s) - \epsilon f_{N+1}'(s)\mathbf{f}_N'(s)^{\mathrm{T}} \left(\boldsymbol{\Sigma}_N^0\right)^{-1} \epsilon f_{N+1}'(s)\mathbf{f}_N'(s). \end{aligned} \tag{S9.10}$$

Using $I_c \equiv \mathbf{f}_N'(s)^{\mathrm{T}}\boldsymbol{\Sigma}_N^{-1}\mathbf{c}_N$, $I_N = \mathbf{f}_N'(s)^{\mathrm{T}}\boldsymbol{\Sigma}_N^{-1}\mathbf{f}_N'(s)$, and the Sherman–Morrison formula for $\boldsymbol{\Sigma}_N^0$,

$$\left(\boldsymbol{\Sigma}_N^0\right)^{-1} = \left(\boldsymbol{\Sigma}_N - \epsilon \mathbf{f}_N'(s)\mathbf{f}_N'(s)^{\mathrm{T}}\right)^{-1} = \boldsymbol{\Sigma}_N^{-1} + \frac{\epsilon \boldsymbol{\Sigma}_N^{-1}\mathbf{f}_N'(s)\mathbf{f}_N'(s)^{\mathrm{T}}\boldsymbol{\Sigma}_N^{-1}}{1 - \epsilon \mathbf{f}_N'(s)^{\mathrm{T}}\boldsymbol{\Sigma}_N^{-1}\mathbf{f}_N'(s)}, \tag{S9.11}$$

the above equation becomes

$$\begin{aligned} &\frac{\det \boldsymbol{\Sigma}_{N+1}^0}{\det \boldsymbol{\Sigma}_N^0} \\ &= \Sigma_{N+1} - \epsilon f_{N+1}'(s)^2 - \left(\mathbf{c}_N^{\mathrm{T}}\boldsymbol{\Sigma}_N^{-1}\mathbf{c}_N + \frac{\epsilon I_c^2}{1 - \epsilon I_N}\right) + 2\epsilon f_{N+1}'(s)\left(I_c + \frac{\epsilon I_c I_N}{1 - \epsilon I_N}\right) - \left(\epsilon f_{N+1}'(s)\right)^2 \left(I_N + \frac{\epsilon I_N^2}{1 - \epsilon I_N}\right) \\ &= \left(\Sigma_{N+1} - \mathbf{c}_N^{\mathrm{T}}\boldsymbol{\Sigma}_N^{-1}\mathbf{c}_N\right) - \frac{\epsilon I_c^2}{1 - \epsilon I_N} + 2\epsilon f_{N+1}'(s) I_c \left(1 + \frac{\epsilon I_N}{1 - \epsilon I_N}\right) - \epsilon f_{N+1}'(s)^2 - \left(\epsilon f_{N+1}'(s)\right)^2 I_N \left(1 + \frac{\epsilon I_N}{1 - \epsilon I_N}\right) \\ &= \left(\Sigma_{N+1} - \mathbf{c}_N^{\mathrm{T}}\boldsymbol{\Sigma}_N^{-1}\mathbf{c}_N\right) - \frac{\epsilon I_c^2}{1 - \epsilon I_N} + 2\epsilon f_{N+1}'(s)\frac{I_c}{1 - \epsilon I_N} - \epsilon f_{N+1}'(s)^2 - \left(\epsilon f_{N+1}'(s)\right)^2 \frac{I_N}{1 - \epsilon I_N} \\ &= \left(\Sigma_{N+1} - \mathbf{c}_N^{\mathrm{T}}\boldsymbol{\Sigma}_N^{-1}\mathbf{c}_N\right) - \frac{\epsilon I_c^2}{1 - \epsilon I_N} + 2\epsilon f_{N+1}'(s)\frac{I_c}{1 - \epsilon I_N} - \epsilon f_{N+1}'(s)^2 \left(1 + \frac{\epsilon I_N}{1 - \epsilon I_N}\right) \\ &= \left(\Sigma_{N+1} - \mathbf{c}_N^{\mathrm{T}}\boldsymbol{\Sigma}_N^{-1}\mathbf{c}_N\right) - \frac{\epsilon}{1 - \epsilon I_N}\left(I_c^2 - 2f_{N+1}'(s)I_c + f_{N+1}'(s)^2\right) \\ &= \left(\Sigma_{N+1} - \mathbf{c}_N^{\mathrm{T}}\boldsymbol{\Sigma}_N^{-1}\mathbf{c}_N\right) - \frac{\epsilon}{1 - \epsilon I_N}\left(I_c - f_{N+1}'(s)\right)^2. \end{aligned} \tag{S9.12}$$

Using Eq. S8.10, the equation above becomes

$$\begin{aligned}\frac{\det \boldsymbol{\Sigma}^0_{N+1}}{\det \boldsymbol{\Sigma}^0_N} &= \omega_{N+1} - \frac{\epsilon}{1-\epsilon I_N}\left(\mathbf{f}'_N(s)^{\mathrm{T}}\boldsymbol{\Sigma}_N^{-1}\mathbf{c}_N - f'_{N+1}(s)\right)^2 \\ &= \omega_{N+1}\frac{\epsilon}{1-\epsilon I_N}\left(\frac{1}{\epsilon} - I_N - \omega_{N+1}^{-1}\left(\mathbf{f}'_N(s)^{\mathrm{T}}\boldsymbol{\Sigma}_N^{-1}\mathbf{c}_N - f'_{N+1}(s)\right)^2\right) \\ &= \omega_{N+1}\frac{\epsilon}{1-\epsilon I_N}\left(\frac{1}{\epsilon} - I_{N+1}\right) \\ &= \omega_{N+1}\frac{1-\epsilon I_{N+1}}{1-\epsilon I_N}\end{aligned} \tag{S9.13}$$

This equation can also be obtained by directly applying the matrix determinant lemma, $\det \boldsymbol{\Sigma}^0_N = \det \boldsymbol{\Sigma}_N \cdot (1-\epsilon \mathbf{f}'_N(s)^{\mathrm{T}}\boldsymbol{\Sigma}_N^{-1}\mathbf{f}'_N(s)) = \det \boldsymbol{\Sigma}_N \cdot (1-\epsilon I_N)$, to both $\det \boldsymbol{\Sigma}^0_{N+1}$ and $\det \boldsymbol{\Sigma}^0_N$, together with $\det \boldsymbol{\Sigma}_{N+1}/\det \boldsymbol{\Sigma}_N = \omega_{N+1}$ (Eq. S8.3).

Note that $\omega_{N+1} > 0$ from Eq. S8.3. Furthermore, with $\epsilon = 1/I_\infty$, the strict bounds $I_N < I_\infty$ and $I_{N+1} < I_\infty$ imply that both the numerator and denominator below are positive:

$$\frac{\det \boldsymbol{\Sigma}^0_{N+1}}{\det \boldsymbol{\Sigma}^0_N} = \omega_{N+1}\frac{I_\infty - I_{N+1}}{I_\infty - I_N} > 0. \tag{S9.14}$$

Therefore, given $\det \boldsymbol{\Sigma}^0_N > 0$, we obtain $\det \boldsymbol{\Sigma}^0_{N+1} > 0$. A single neuron ($N = 1$) with a positive variance Σ_1 satisfies $\det \Sigma^0_1 = \Sigma_1 - \epsilon f'_1(s)^2 > 0$ since $1/\epsilon > f'_1(s)^2\Sigma_1^{-1} = I_1$. Thus, $\det \boldsymbol{\Sigma}^0_2 > 0$ for the population of two neurons. Then, according to Sylvester's criterion, $\boldsymbol{\Sigma}^0_2$ is positive definite. Recursively applying this relation to increasing the number of neurons up to $N+1$ guarantees that all of the leading principal minors of $\boldsymbol{\Sigma}^0_{N+1}$ are positive, proving that $\boldsymbol{\Sigma}^0_{N+1}$ is positive definite for any N.

text S10. Differential correlations induce linearly scaling eigenvalues

In this note, we show that if $\mathbf{\Sigma}_N^0$ leads to the unbounded LFI for all directions of $\mathbf{f}'_N(s)$, by adding differential correlations, only one of the eigenvectors of $\mathbf{\Sigma}_N$ will be substantially aligned with $\mathbf{f}'_N(s)$ and only the eigenvalue associated with this eigenvector will linearly increase with N. Otherwise, if I_0 is unbounded only for specific signal directions and $\mathbf{f}'_N(s)$ retains a nonvanishing component outside the linear-eigenvalue subspace of $\mathbf{\Sigma}_N^0$, there are multiple linear eigenvalues in $\mathbf{\Sigma}_N$ whose eigenvectors asymptotically span $\mathbf{f}'_N(s)$. These results show that alignment of the signal with the linear-eigenvalue subspace is necessary to limit the information.

If differential correlations exist, for any subsample of neurons, $\mathbf{\Sigma}_N^0$ must exist and because it is a covariance matrix, it can be written in terms of its eigenvalues $\gamma_{i,N}$ and eigenvectors $\mathbf{v}_{i,N}$ as

$$\mathbf{\Sigma}_N^0 = \sum_{i=1}^{N} \gamma_{i,N} \mathbf{v}_{i,N} \mathbf{v}_{i,N}^{\mathrm{T}}. \tag{S10.1}$$

Since the set of orthonormal eigenvectors is complete, we can write $\mathbf{f}'_N(s)$ as a linear combination of the eigenvectors of $\mathbf{\Sigma}_N^0$:

$$\mathbf{f}'_N(s) = |\mathbf{f}'_N(s)| \sum_{i=1}^{N} \cos\phi_{i,N} \mathbf{v}_{i,N} \tag{S10.2}$$

with $\sum_i \cos^2\phi_{i,N} = 1$, in which $\phi_{i,N}$ is the angle between the i-th eigenvector of $\mathbf{\Sigma}_N^0$ and $\mathbf{f}'_N(s)$. We can also write

$$\begin{aligned}
\mathbf{\Sigma}_N =& \mathbf{\Sigma}_N^0 + \epsilon \mathbf{f}'_N(s)\mathbf{f}'_N(s)^{\mathrm{T}} \\
=& \sum_{i=1}^{N} \gamma_{i,N} \mathbf{v}_{i,N}\mathbf{v}_{i,N}^{\mathrm{T}} + \epsilon|\mathbf{f}'_N(s)|^2 \sum_{i=1}^{N}\sum_{j=1}^{N} \cos\phi_{i,N}\cos\phi_{j,N}\mathbf{v}_{i,N}\mathbf{v}_{j,N}^{\mathrm{T}} \\
=& \sum_{i=1}^{N}\sum_{j=1}^{N} (\delta_{ij}\gamma_{i,N} + \epsilon|\mathbf{f}'_N(s)|^2 \cos\phi_{i,N}\cos\phi_{j,N})\mathbf{v}_{i,N}\mathbf{v}_{j,N}^{\mathrm{T}}.
\end{aligned} \tag{S10.3}$$

We multiply the above equation from the right side by $\mathbf{f}'_N(s)$

$$\begin{aligned}
\mathbf{\Sigma}_N \mathbf{f}'_N(s) =& |\mathbf{f}'_N(s)| \sum_{i=1}^{N}\sum_{j=1}^{N}\sum_{k=1}^{N} \cos\phi_{k,N}(\delta_{ij}\gamma_{i,N} + \epsilon|\mathbf{f}'_N(s)|^2\cos\phi_{i,N}\cos\phi_{j,N})\mathbf{v}_{i,N}\mathbf{v}_{j,N}^{\mathrm{T}}\mathbf{v}_{k,N} \\
=& |\mathbf{f}'_N(s)| \sum_{i=1}^{N}\sum_{j=1}^{N} \cos\phi_{j,N}(\delta_{ij}\gamma_{i,N} + \epsilon|\mathbf{f}'_N(s)|^2\cos\phi_{i,N}\cos\phi_{j,N})\mathbf{v}_{i,N} \\
=& |\mathbf{f}'_N(s)| \sum_{i=1}^{N} (\gamma_{i,N} + \epsilon|\mathbf{f}'_N(s)|^2)\cos\phi_{i,N}\mathbf{v}_{i,N}.
\end{aligned} \tag{S10.4}$$

Furthermore, if $\mathbf{\Sigma}_N^0$ is independent of $\mathbf{f}'_N(s)$ and gives unbounded LFI for every signal direction with $|\mathbf{f}'_N(s)|^2 = AN$, then all $\gamma_{i,N}$ must be sublinear functions of N. Indeed, choosing $\mathbf{f}'_N(s) = \sqrt{AN}\,\mathbf{v}_{1,N}$ gives $I_0(N) = AN/\gamma_{1,N} \to \infty$, so $\gamma_{1,N}/N \to 0$; by eigenvalue ordering, all $\gamma_{i,N}$ are sublinear.

Therefore,

$$\begin{aligned}
\mathbf{\Sigma}_N \mathbf{f}'_N(s) =& |\mathbf{f}'_N(s)| \sum_{i=1}^{N} \epsilon|\mathbf{f}'_N(s)|^2\cos\phi_{i,N}\mathbf{v}_{i,N} + o\big(N|\mathbf{f}'_N(s)|\big) \\
=& \epsilon|\mathbf{f}'_N(s)|^2\mathbf{f}'_N(s) + o\big(N|\mathbf{f}'_N(s)|\big).
\end{aligned} \tag{S10.5}$$

Here and below, $o(a_N)$ denotes a term whose magnitude divided by a_N tends to zero as $N \to \infty$. Because the largest eigenvalue of $\mathbf{\Sigma}_N^0$ is $o(N)$, adding $\mathbf{\Sigma}_N^0$ changes the eigenvalues of the rank-one differential-correlation term by at most $o(N)$. Therefore, $\lambda_{1,N} = \epsilon|\mathbf{f}'_N(s)|^2 + o(N)$ and $\lambda_{i,N} = o(N)$ for $i \geq 2$. This result states that, in this limit, $\mathbf{f}'_N(s)/|\mathbf{f}'_N(s)|$ asymptotically aligns with the leading eigenvector of $\mathbf{\Sigma}_N$. In other words, $\mathbf{f}'_N(s)$ is spanned by only this specific eigenvector of $\mathbf{\Sigma}_N$. Namely, if $\mathbf{\Sigma}_N^0$ is independent of $\mathbf{f}'_N(s)$ and gives unbounded LFI for every signal direction, we expect that only one of the eigenvectors becomes increasingly aligned with $\mathbf{f}'_N(s)$ as N increases. Specifically,

one of the direction cosines ($i = 1$) dominates the spectrum of cosines (i.e., $\lim_{N\to\infty} \cos^2\theta_{1,N} = 1$) and the rest of the cosines go to zero as N increases. In other words, in the large-N limit, we have

$$W_L(N) = \cos^2\theta_{1,N} = 1 \text{ and } |L| = 1, \tag{S10.6}$$

in which $|L|$ is the number of elements (cardinality) of the set L.

On the other hand, if $I_0(N) = \mathbf{f}'_N(s)^{\mathrm{T}}(\mathbf{\Sigma}^0_N)^{-1}\mathbf{f}'_N(s)$ is unbounded only for specific signal directions, not all $\gamma_{i,N}$ are sublinear functions. If $\mathbf{f}'_N(s)$ retains a nonvanishing component outside the linear-eigenvalue subspace of $\mathbf{\Sigma}^0_N$, the differential-correlation term produces an additional linearly scaling eigenvalue. Consequently, the linear-eigenvalue subspace of $\mathbf{\Sigma}_N$ is multidimensional and asymptotically contains $\mathbf{f}'_N(s)$. A particularly interesting case is $\mathbf{\Sigma}_N$ exhibiting infinitely many linear eigenvalues for which the LFI boundedness is equivalent to restricting the orientation of the signal vector to a particular subspace, e.g., $\gamma > \gamma_c$ if $\cos^2(\theta_{i,N}) \sim \lambda^{\gamma}_{i,N}$.

text S11. Noise of populations expressing finite limiting LFI may not contain differential correlations

In this note, we demonstrate that the differential correlations are not the unique form of information-limiting correlations for covariance matrices even if they are subject to the linearly increasing total variance. A specific decomposition of the covariance matrix $\mathbf{\Sigma}_N$ is shown below, which resembles the structure of differential correlations (having the sum of two matrices, one that limits information and the other that does not limit), but has a potentially higher-rank information-limiting matrix added to the non-information-limiting component. We then give a formal disproof.

Given that the linear and sublinear eigenvalues of $\mathbf{\Sigma}_N$ are respectively written as $\lambda_{i,N} = b_i N + F_i(N)$ ($i \in L$) and $\lambda_{i,N} = G_i(N)$ ($i \in S_b$) (See text S2), $\mathbf{\Sigma}_N$ can in general be decomposed as

$$\begin{aligned}\mathbf{\Sigma}_N &= \sum_{i\in L}[b_i N + F_i(N)]\mathbf{u}_{i,N}\mathbf{u}_{i,N}^T + \sum_{i\in S_b} G_i(N)\mathbf{u}_{i,N}\mathbf{u}_{i,N}^T \\ &= \left(\sum_{i\in L}\tilde{F}_i(N)\mathbf{u}_{i,N}\mathbf{u}_{i,N}^T + \sum_{i\in S_b} G_i(N)\mathbf{u}_{i,N}\mathbf{u}_{i,N}^T\right) + \sum_{i\in L}[b_i N + F_i(N) - \tilde{F}_i(N)]\mathbf{u}_{i,N}\mathbf{u}_{i,N}^T, \end{aligned} \tag{S11.1}$$

where $\tilde{F}_i(N)$ ($i \in L$) are sublinear functions satisfying the conditions below. Hence, we obtain the following expression for $\mathbf{\Sigma}_N$:

$$\mathbf{\Sigma}_N = \tilde{\mathbf{\Sigma}}_N^0 + \mathbf{\Sigma}_{\mathrm{IL}}, \tag{S11.2}$$

where the first term is

$$\begin{aligned}\tilde{\mathbf{\Sigma}}_N^0 &\equiv \sum_{i\in L}\tilde{F}_i(N)\mathbf{u}_{i,N}\mathbf{u}_{i,N}^T + \sum_{i\in S_b} G_i(N)\mathbf{u}_{i,N}\mathbf{u}_{i,N}^T \\ &= \sum_{i=1}^{N}\tilde{\lambda}_{i,N}\mathbf{u}_{i,N}\mathbf{u}_{i,N}^T. \end{aligned} \tag{S11.3}$$

Here we introduced $\tilde{\lambda}_{i,N}$ defined as $\tilde{\lambda}_{i,N} = \tilde{F}_i(N)$ if $i \in L$ and $\tilde{\lambda}_{i,N} = G_i(N)$ if $i \in S_b$. Note that we can choose $\tilde{F}_i(N)$ to be sublinear and satisfy $0 < \tilde{F}_i(N) < \lambda_{i,N}$ ($i \in L$). Then, $\tilde{\mathbf{\Sigma}}_N^0$ is a positive definite matrix. While $\tilde{\mathbf{\Sigma}}_N^0$ has the same set of eigenvectors as in $\mathbf{\Sigma}_N$, all of its eigenvalues are sublinear functions of N. Consequently, $\tilde{\mathbf{\Sigma}}_N^0$ results in unbounded LFI for any $\mathbf{f}'_N(s)$ (text S1). The second term is

$$\mathbf{\Sigma}_{\mathrm{IL}} \equiv \sum_{i\in L}(b_i N + F_i(N) - \tilde{F}_i(N))\mathbf{u}_{i,N}\mathbf{u}_{i,N}^T, \tag{S11.4}$$

which is a rank $|L|$ matrix and is positive semidefinite because $\tilde{F}_i(N) < \lambda_{i,N}$ for all $i \in L$. This decomposition is applicable to any covariance matrix, including the case that $\mathbf{f}'_N(s)$ and $\mathbf{\Sigma}_N$ result in the saturation of information. In this case, the covariance matrix $\mathbf{\Sigma}_N$ was expressed as the sum of two matrices, where $\tilde{\mathbf{\Sigma}}_N^0$ leads to unbounded LFI irrespective of the orientation of $\mathbf{f}'_N(s)$, and a rank $|L|$ matrix that is responsible for limiting the information. Note that this decomposition is not unique: We can construct various decompositions using sublinear functions $\tilde{F}_i(N)$ satisfying $0 < \tilde{F}_i(N) < \lambda_{i,N}$.

An example of decomposing a more specific covariance matrix disproves the presence of differential correlation in a covariance matrix subject to the linearly increasing total variance. Suppose that the kth eigenvector $\mathbf{u}_{k,N}$ ($k \in L$) of $\mathbf{\Sigma}_N$ perfectly aligns with the direction of $\mathbf{f}'_N(s)$. Such a covariance matrix results in the bounded information because $I_N = AN/[b_k N + F_k(N)] \to A/b_k$. Here, we note that, since $\mathbf{u}_{k,N} = \mathbf{f}'_N(s)/|\mathbf{f}'_N(s)|$, we have

$$\mathbf{u}_{k,N}\mathbf{u}_{k,N}^T = \frac{\mathbf{f}'_N(s)\mathbf{f}'_N(s)^T}{|\mathbf{f}'_N(s)|^2} = \frac{\mathbf{f}'_N(s)\mathbf{f}'_N(s)^T}{AN}. \tag{S11.5}$$

Using this relation, the covariance matrix is decomposed as

$$\begin{aligned}\mathbf{\Sigma}_N &= \sum_{i\in L}[b_i N + F_i(N)]\mathbf{u}_{i,N}\mathbf{u}_{i,N}^T + \sum_{i\in S_b} G_i(N)\mathbf{u}_{i,N}\mathbf{u}_{i,N}^T \\ &= \left(F_k(N)\mathbf{u}_{k,N}\mathbf{u}_{k,N}^T + \sum_{i\in L,\, i\neq k}[b_i N + F_i(N)]\mathbf{u}_{i,N}\mathbf{u}_{i,N}^T + \sum_{i\in S_b} G_i(N)\mathbf{u}_{i,N}\mathbf{u}_{i,N}^T \right) + b_k N \mathbf{u}_{k,N}\mathbf{u}_{k,N}^T \\ &= \tilde{\mathbf{\Sigma}}_N^0 + \epsilon \mathbf{f}_N'(s)\mathbf{f}_N'(s)^T, \end{aligned} \quad \text{(S11.6)}$$

where we defined $\epsilon \equiv b_k/A$ and

$$\tilde{\mathbf{\Sigma}}_N^0 \equiv F_k(N)\mathbf{u}_{k,N}\mathbf{u}_{k,N}^T + \sum_{i\in L,\, i\neq k}[b_i N + F_i(N)]\mathbf{u}_{i,N}\mathbf{u}_{i,N}^T + \sum_{i\in S_b} G_i(N)\mathbf{u}_{i,N}\mathbf{u}_{i,N}^T. \quad \text{(S11.7)}$$

If $F_k(N) > 0$, this matrix is positive definite. Unlike the previous decomposition, we did not exclude the linear eigenvalues except for k from $\tilde{\mathbf{\Sigma}}_N^0$. This is because $\mathbf{u}_{i,N}$ ($i \in L,\ i \neq k$) are orthogonal to $\mathbf{f}_N'(s)$; therefore, $\tilde{\mathbf{\Sigma}}_N^0$ still results in unbounded LFI because $I_0(N) = AN/F_k(N) \to \infty$ (See also text S10). Thus, such a covariance $\mathbf{\Sigma}_N$ is written as a sum of a non-saturating (invertible) covariance matrix and the differential correlations. In other words, $\mathbf{\Sigma}_N$ contains differential correlations.

However, $F_k(N)$ can be nonpositive. The eigenvalues of $\mathbf{\Sigma}_N$, including the linear eigenvalues $\lambda_{i,N} = b_i N + F_i(N)$ ($i \in L$), must be positive so that $\mathbf{\Sigma}_N$ is positive definite. Hence, the sublinear functions $F_i(N)$ in the linear eigenvalues must satisfy $F_i(N) > -b_i N$, which allows $F_i(N)$ to be either zero or negative. If $F_k(N) \le 0$, $\tilde{\mathbf{\Sigma}}_N^0$ is no longer a positive definite matrix. If $F_k(N) = 0$, i.e., $\lambda_{k,N} = b_k N$, the rank of $\tilde{\mathbf{\Sigma}}_N^0$ becomes $N-1$, and $\tilde{\mathbf{\Sigma}}_N^0$ is not invertible. With $\epsilon = 1/I_\infty = b_k/A$, the bounded covariance $\mathbf{\Sigma}_N$ having these properties cannot be decomposed into a sum of an (invertible) covariance matrix with unbounded LFI and the differential correlations. This example disproves the statement that differential correlations are the only information-limiting correlations.

In what follows, we give a formal disproof of the differential correlations using the example above, following an approach that proved the presence of differential correlations for the subsampled populations with saturating LFI (text S9). We assume that we have $\mathbf{\Sigma}_N$ that results in the bounded information with its limit being given by $\lim_{N\to\infty} I_N = 1/\epsilon$, namely $\epsilon = 1/\lim_{N\to\infty} I_N$. The presence of the differential correlations is guaranteed if we can decompose $\mathbf{\Sigma}_N$ in the form of Eq. S9.1, where $\mathbf{\Sigma}_N^0$ is a positive definite covariance matrix.

Using eigenvectors of $\mathbf{\Sigma}_N$ and $\mathbf{f}_N'(s) = |\mathbf{f}_N'(s)| \sum_{i=1}^N \cos\theta_{i,N}\mathbf{u}_{i,N}$, $\mathbf{\Sigma}_N^0$ is written as

$$\begin{aligned}\mathbf{\Sigma}_N^0 &= \mathbf{\Sigma}_N - \epsilon \mathbf{f}_N'(s)\mathbf{f}_N'(s)^{\mathrm{T}} \\ &= \sum_{i=1}^N \lambda_{i,N}\mathbf{u}_{i,N}\mathbf{u}_{i,N}^{\mathrm{T}} - \epsilon AN \sum_{i,j}\cos(\theta_{i,N})\cos(\theta_{j,N})\mathbf{u}_{i,N}\mathbf{u}_{j,N}^{\mathrm{T}}\end{aligned}$$

A positive definite matrix $\mathbf{\Sigma}_N$ can be diagonalized by an orthogonal matrix as $\mathbf{\Sigma}_N = \mathbf{U}_N\mathbf{W}_N\mathbf{U}_N^{\mathrm{T}}$, where $\mathbf{W}_N$ is a diagonal matrix with its entries being the eigenvalues of $\mathbf{\Sigma}_N$. The ith element of the jth column $\mathbf{w}_{j,N}$ of $\mathbf{W}_N$ is given by $w_{ij,N} = \lambda_{i,N}\delta_{ij}$. Then from

$$\mathbf{\Sigma}_N^0 = \mathbf{U}_N(\mathbf{U}_N^{\mathrm{T}}\mathbf{\Sigma}_N^0\mathbf{U}_N)\mathbf{U}_N^{\mathrm{T}}, \quad \text{(S11.8)}$$

we investigate $\mathbf{W}_N^0 \equiv \mathbf{U}_N^{\mathrm{T}}\mathbf{\Sigma}_N^0\mathbf{U}_N$ because if $\mathbf{W}_N^0$ is a positive definite matrix, so is $\mathbf{\Sigma}_N^0$. Noting $\mathbf{U}_N\mathbf{W}_N = \mathbf{\Sigma}_N\mathbf{U}_N$ or $\mathbf{U}_N[\mathbf{w}_{1,N},\ldots,\mathbf{w}_{N,N}] = \mathbf{\Sigma}_N[\mathbf{u}_{1,N},\ldots,\mathbf{u}_{N,N}]$, we have $\mathbf{U}_N\mathbf{w}_{i,N} = \mathbf{\Sigma}_N\mathbf{u}_{i,N}$, from which we obtain

$$\mathbf{U}_N^{\mathrm{T}}\mathbf{u}_{i,N} = \mathbf{U}_N^{\mathrm{T}}\mathbf{\Sigma}_N^{-1}\mathbf{U}_N\mathbf{w}_{i,N} = \mathbf{W}_N^{-1}\mathbf{w}_{i,N} = \lambda_{i,N}^{-1}\mathbf{w}_{i,N}. \quad \text{(S11.9)}$$

Hence,

$$\begin{aligned}\mathbf{W}_N^0 &\equiv \mathbf{U}_N^{\mathrm{T}}\mathbf{\Sigma}_N^0\mathbf{U}_N \\ &= \sum_{i=1}^N \lambda_{i,N}\lambda_{i,N}^{-2}\mathbf{w}_{i,N}\mathbf{w}_{i,N}^{\mathrm{T}} - \epsilon AN\sum_{i,j}\cos(\theta_{i,N})\cos(\theta_{j,N})\lambda_{i,N}^{-1}\lambda_{j,N}^{-1}\mathbf{w}_{i,N}\mathbf{w}_{j,N}^{\mathrm{T}}, \\ &= \sum_{i,j=1}^N \left(\delta_{ij}\lambda_{i,N}^{-1} - \epsilon AN\cos(\theta_{i,N})\cos(\theta_{j,N})\lambda_{i,N}^{-1}\lambda_{j,N}^{-1}\right)\mathbf{w}_{i,N}\mathbf{w}_{j,N}^{\mathrm{T}}\end{aligned} \quad \text{(S11.10)}$$

The (k,l) element of $\mathbf{W}_N^0$ is computed as

$$
\begin{aligned}
[\mathbf{W}_N^0]_{kl} &= \sum_{i,j} \left(\delta_{ij} \lambda_{i,N}^{-1} - \epsilon AN \cos(\theta_{i,N}) \cos(\theta_{j,N}) \lambda_{i,N}^{-1} \lambda_{j,N}^{-1} \right) w_{ik,N} w_{jl,N} \\
&= \left(\delta_{kl} \lambda_{k,N}^{-1} - \epsilon AN \cos(\theta_{k,N}) \cos(\theta_{l,N}) \lambda_{k,N}^{-1} \lambda_{l,N}^{-1} \right) \lambda_{k,N} \lambda_{l,N} \\
&= \delta_{kl} \lambda_{k,N} - \epsilon AN \cos(\theta_{k,N}) \cos(\theta_{l,N}) \\
&= \begin{cases} \lambda_{k,N} - \epsilon AN \cos^2(\theta_{k,N}) & k = l \\ -\epsilon AN \cos(\theta_{k,N}) \cos(\theta_{l,N}) & k \neq l \end{cases}
\end{aligned} \tag{S11.11}
$$

Hence, for $\mathbf{W}_N^0$ to be a positive definite matrix, it must satisfy

$$
\boldsymbol{v}_N^{\mathrm{T}} \mathbf{W}_N^0 \boldsymbol{v}_N = \sum_i \left(\lambda_{i,N} - \epsilon AN \cos^2(\theta_{i,N}) \right) v_{i,N}^2 - \epsilon AN \sum_{i \neq j} \cos(\theta_{i,N}) \cos(\theta_{j,N}) v_{i,N} v_{j,N} > 0 \tag{S11.12}
$$

for any nonzero real vector $\boldsymbol{v}_N$ ($\boldsymbol{v}_N \neq 0$) when $\epsilon = 1/I_\infty$.

We examine this condition with the simple case discussed above, in which one linear eigenvalue occupies a cosine direction to $\mathbf{f}'_N(s)$. For simplicity, we consider that the first linear eigenvalue occupies the cosine direction ($\cos^2 \theta_{1,N} = 1$). In this case,

$$
I_N = \sum_{i=1}^{N} \frac{|\mathbf{f}'_N(s)|^2 \cos^2 \theta_{i,N}}{\lambda_{i,N}} = \frac{AN}{b_1 N + F_1(N)} \tag{S11.13}
$$

from which we obtain $I_\infty = A/b_1$. Hence we use $\epsilon = b_1/A$.

The quadratic term then becomes

$$
\begin{aligned}
\boldsymbol{v}_N^{\mathrm{T}} \mathbf{W}_N^0 \boldsymbol{v}_N &= (\lambda_{1,N} - \epsilon AN) v_{1,N}^2 + \sum_{i=2}^{N} \lambda_{i,N} v_{i,N}^2 \\
&= (F_1(N) + N(b_1 - \epsilon A)) v_{1,N}^2 + \sum_{i=2}^{N} \lambda_{i,N} v_{i,N}^2 \\
&= F_1(N) v_{1,N}^2 + \sum_{i=2}^{N} \lambda_{i,N} v_{i,N}^2,
\end{aligned} \tag{S11.14}
$$

If $F_1(N) > 0$, $\boldsymbol{\Sigma}_N^0$ is positive definite. However, if $0 \geq F_1(N) > -b_1 N$, $\boldsymbol{\Sigma}_N^0$ is not positive definite. When $F_1(N) = 0$, $\boldsymbol{\Sigma}_N^0$ is not invertible, which also violates the condition for the differential correlations. We note that when $F_1(N) = \lambda_{1,N} - \epsilon AN < 0$, we have

$$
I_N = \frac{AN}{\lambda_{1,N}} > \frac{1}{\epsilon} = \frac{A}{b_1} = I_\infty \tag{S11.15}
$$

So the LFI approaches I_∞ from above.

With this example, we corroborated that if a linear eigenvalue of the covariance matrix occupies the angular occupation, and the sublinear component of the linear eigenvalue is not positive, the covariance matrix does not contain the differential correlations with a positive-definite residual at $\epsilon = 1/I_\infty$.

text S12. Bias correction for covariance eigenvalue estimation

It is well known from the random matrix theory that the estimated covariance eigenvalues with finite sample size tend to push each other away on the real positive axis, so that the domain of the estimated eigenvalues is larger than the domain of the actual eigenvalues, leading to bias in their estimates, i.e., underestimation of small eigenvalues, and overestimation of large ones. Here, we present a method for correcting this bias. Throughout this note, we consider a fixed population size N. For notational simplicity, we therefore suppress the explicit N-dependence in quantities such as $\lambda_{i,N}$ and write them as λ_i.

Let $r_{i,t}(s_j)$ denote the response of neuron i on trial t to stimulus s_j. The population response vector for a subpopulation of size N is $\mathbf{r}_t(s_j) = [r_{1,t}(s_j), r_{2,t}(s_j), \dots, r_{N,t}(s_j)]^{\mathrm{T}}$, its average across trials is $\boldsymbol{\mu}(s_j)$, and the direct estimate of the covariance matrix is

$$\hat{\boldsymbol{\Sigma}}(s_j) = \frac{1}{T-1}\sum_{t=1}^{T}(\mathbf{r}_t(s_j) - \boldsymbol{\mu}(s_j))(\mathbf{r}_t(s_j) - \boldsymbol{\mu}(s_j))^{\mathrm{T}}. \tag{S12.1}$$

Here, we assume that this estimate is a sample of a Wishart distribution (*17*),

$$\hat{\boldsymbol{\Sigma}}(s_j) \sim \mathcal{W}\left(\frac{\boldsymbol{\Sigma}(s_j)}{T-1}, T-1\right), \tag{S12.2}$$

where $\boldsymbol{\Sigma}(s_j)$ is the actual covariance matrix. In this paper, we have two sets of stimuli (s_1, s_2), and we use the estimate of the covariance as the average of the two covariance matrices, i.e., $\hat{\boldsymbol{\Sigma}} = (\,\hat{\boldsymbol{\Sigma}}(s_1) + \hat{\boldsymbol{\Sigma}}(s_2)\,)/2$. If the two stimulus groups have the same true covariance, $\boldsymbol{\Sigma}(s_1) = \boldsymbol{\Sigma}(s_2) = \boldsymbol{\Sigma}$, then

$$\hat{\boldsymbol{\Sigma}} \sim \mathcal{W}\left(\frac{\boldsymbol{\Sigma}}{2T-2}, 2T-2\right). \tag{S12.3}$$

Eigenvalues of $\hat{\boldsymbol{\Sigma}}$ are the naive and biased estimates of the eigenvalues of $\boldsymbol{\Sigma}$. Let $\{\hat{\lambda}_1, \hat{\lambda}_2, \dots, \hat{\lambda}_N\}$ be the ranked eigenvalues of $\hat{\boldsymbol{\Sigma}}$ and $\{\lambda_1, \lambda_2, \dots, \lambda_N\}$ be those of $\boldsymbol{\Sigma}$. Because the Wishart distribution is closed under orthogonal unitary transformations, we can consider the diagonalization of $\boldsymbol{\Sigma}$ as

$$\boldsymbol{\Sigma} = \mathbf{Q}^{\mathrm{T}}\boldsymbol{\Lambda}\mathbf{Q} \tag{S12.4}$$

in which $\boldsymbol{\Lambda}$ is a diagonal matrix with $\{\lambda_1, \lambda_2, \dots, \lambda_N\}$ as its diagonal elements. To do the eigenvalue bias corrections, we work in the coordinate system in which $\boldsymbol{\Sigma}$ is diagonalized (principal component basis). In this coordinate system, we have

$$\hat{\boldsymbol{\Sigma}}' \sim \mathcal{W}\left(\frac{\boldsymbol{\Lambda}}{2T-2}, 2T-2\right), \tag{S12.5}$$

where $\hat{\boldsymbol{\Sigma}}' = \mathbf{Q}\hat{\boldsymbol{\Sigma}}\mathbf{Q}^{\mathrm{T}}$. Note that the eigenvalues of $\hat{\boldsymbol{\Sigma}}$ and $\hat{\boldsymbol{\Sigma}}'$ are the same.

Our bias correction approach is based on Eq. S12.5. Our goal is to find the set of ranked eigenvalues $\{\lambda_1, \lambda_2, \dots, \lambda_N\}$ so that samples of the distribution exhibit the same eigenvalue spectrum as the naive estimates $\{\hat{\lambda}_1, \hat{\lambda}_2, \dots, \hat{\lambda}_N\}$. This goal was achieved by utilizing an iterative shrinkage algorithm. We start with an initial guess for $\boldsymbol{\Lambda}$. The algorithm takes the following steps

1. Initial guess for bias-corrected eigenvalues is the naive estimates $\boldsymbol{\Lambda}_{bc} = \hat{\boldsymbol{\Lambda}}$
2. Generate m samples $\mathbf{S}'_j \sim \mathcal{W}\left(\frac{\boldsymbol{\Lambda}_{bc}}{2T-2}, 2T-2\right)$, $j = 1, 2, \dots, m$, from the Wishart distribution in Eq. S12.5.
3. Evaluate the average of ranked eigenvalues of $\mathbf{S}'_j$ across all m samples as $\{\lambda'_1, \lambda'_2, \dots, \lambda'_N\}$.
4. Update the diagonal elements of $\boldsymbol{\Lambda}_{bc}$ by

$$\boldsymbol{\Lambda}_{bc,ii} \leftarrow \boldsymbol{\Lambda}_{bc,ii}\left(\frac{\hat{\lambda}_i}{\lambda'_i}\right)^{\nu} \tag{S12.6}$$

We repeat steps 2–4 until convergence. Here, m is equal to the number of population subsamples used in the naive estimation of the eigenvalues. This algorithm converges when the eigenvalues of $\mathbf{S}'$ become equal to those of $\hat{\boldsymbol{\Sigma}}$. Note that if $\hat{\lambda}_i > \lambda'_i$ the bias-corrected eigenvalues get updated

to larger numbers and vice versa if $\hat{\lambda}_i < \lambda'_i$. The exponent ν can be seen as a learning rate in the log scale, which is selected to be small enough to avoid large steps. In the end we find a set of bias-corrected eigenvalues ($\{\lambda_{bc,1}, \lambda_{bc,2}, \dots, \lambda_{bc,N}\}$) for which the sampled eigenvalues $\{\lambda'_1, \lambda'_2, \dots, \lambda'_N\}$ are equivalent to the naive estimates $\{\hat{\lambda}_1, \hat{\lambda}_2, \dots, \hat{\lambda}_N\}$ so that the ratio

$$\left(\frac{\hat{\lambda}_i}{\lambda'_i}\right) \tag{S12.7}$$

becomes equal to one for all components.

text S13. Bias correction for estimating angular occupations

In this note, we provide a bias-correction algorithm for estimating angular occupations. As in text S12, we consider a fixed population size N and therefore suppress the explicit N-dependence of quantities for notational simplicity.

The error in estimating the angles θ_i between the signal vector $\delta\mathbf{f}$ and the eigenvectors of the covariance matrix $\mathbf{v}_i$ leads to biased estimates of the angular occupations $\cos^2\theta_i$. This bias arises from two sources. First, as illustrated in the figure below, the estimated eigenvector $\hat{\mathbf{v}}_i$ is oriented differently from the true eigenvector $\mathbf{v}_i$. Second, the estimated vector $\delta\boldsymbol{\mu}$, used as an approximation of $\delta\mathbf{f}$, has a different orientation from the true vector.

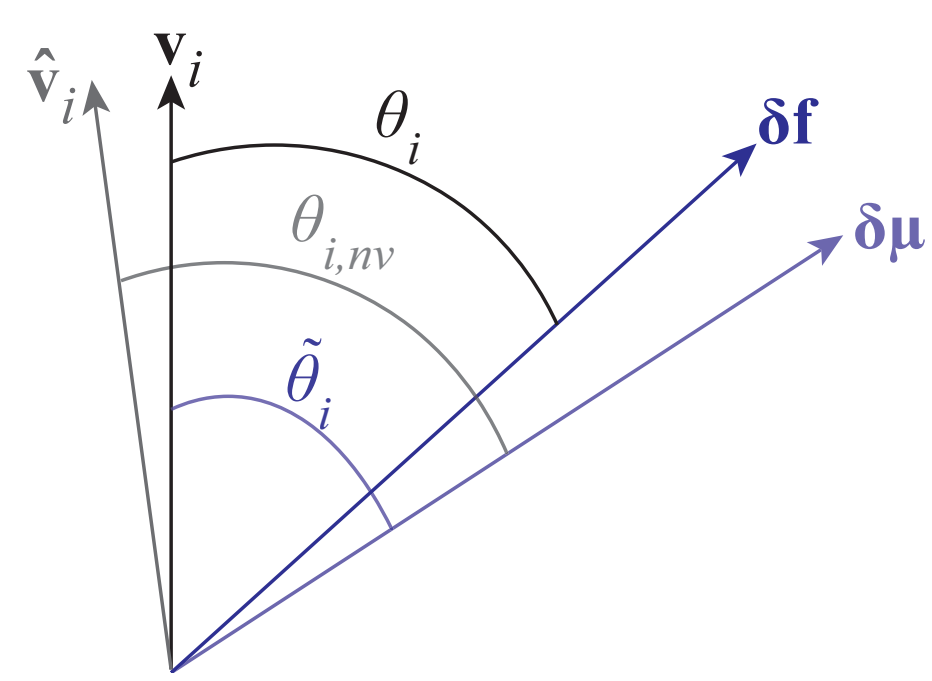

As shown in the above figure, we denote $\theta_{i,nv}$, the naive estimate of θ_i, as the angle between $\delta\boldsymbol{\mu}$ and $\hat{\mathbf{v}}_i$. Similarly, we denote the angle between $\delta\boldsymbol{\mu}$ and the actual eigenvector $\mathbf{v}_i$ as $\tilde{\theta}_i$. To correct the bias in angular occupations, we use a two-step method. First, we make the correction resulting from the error in the estimate of $\mathbf{v}_i$. In other words, the goal in the first step is to find $\cos^2\tilde{\theta}_i$. In the second step, we take $\cos^2\tilde{\theta}_i$ evaluated in the first step and correct for the bias caused by the error in estimation of $\delta\mathbf{f}$.

Angular occupation bias correction: To perform this correction, we adopt a recursive procedure analogous to the eigenvalue bias correction. Specifically, we seek the underlying true squared cosine values $\cos^2\tilde{\theta}_i$ that would produce the observed naive estimates $\cos^2\theta_{i\,\mathrm{nv}}$ under the alignment implied by the Wishart samples of covariance eigenvectors. To do this, we work in the principal basis coordinates that give a diagonal representation of the true covariance matrix $\boldsymbol{\Sigma}$. In this coordinate system, the normalized vector $\delta\bar{\boldsymbol{\mu}} = \delta\boldsymbol{\mu}/|\delta\boldsymbol{\mu}|$ is represented by the vector of directional cosines that can have either positive or negative elements. Because we are focusing on cosine squares, the signs are irrelevant here, and we assume all cosines are positive. The algorithm proceeds as follows:

1. Initial guess for bias-corrected angular occupations is the naive estimates $\cos^2\tilde{\theta}_i \leftarrow \cos^2\theta_{i\,\mathrm{nv}}$
2. Generate m samples from the Wishart $\hat{\boldsymbol{\Sigma}}_j \sim \mathcal{W}\left(\frac{\boldsymbol{\Lambda}_{bc}}{2T-2}, 2T-2\right)$ with $j = 1, 2, \ldots, m$, where $\boldsymbol{\Lambda}_{bc}$ is the diagonal matrix of bias-corrected eigenvalues obtained from the eigenvalue bias-correction algorithm.
3. Evaluate the eigenvectors of every sampled covariance matrix $\hat{\boldsymbol{\Sigma}}_j$ as $\{\hat{\mathbf{v}}_{j,1}, \hat{\mathbf{v}}_{j,2}, \ldots, \hat{\mathbf{v}}_{j,N}\}$ and find the sampled cosine squares as the squared dot product of these eigenvectors and $\delta\bar{\boldsymbol{\mu}}$ as $(\sqrt{\cos^2\theta'_{1,j}}, \sqrt{\cos^2\theta'_{2,j}}, \ldots, \sqrt{\cos^2\theta'_{N,j}})^{\mathrm{T}}$.
4. Evaluate the average of $\cos^2\theta'_{i,j}$ ranked according to eigenvalues of $\hat{\boldsymbol{\Sigma}}_j$ across all m samples as $\{\cos^2\theta'_1, \cos^2\theta'_2, \ldots, \cos^2\theta'_N\}$.
5. Update the angular occupations by

$$\cos^2\tilde{\theta}_i \leftarrow \cos^2\tilde{\theta}_i \left(\frac{\cos^2\theta_{i\,\mathrm{nv}}}{\cos^2\theta'_i}\right)^{\xi} \tag{S13.1}$$

6. Normalize $\cos^2\tilde{\theta}_i \leftarrow \cos^2\tilde{\theta}_i / \sum_{i=1}^{N}\cos^2\tilde{\theta}_i$.

We repeat steps 2–6 until convergence. Similar to the eigenvalue bias-correction algorithm, m is equal to the number of population subsamples used in the naive estimation of the cosine squares. This algorithm converges when $\cos^2\theta'_i$ becomes equal to the naive estimate $\cos^2\theta_{i_{\mathrm{nv}}}$ for all i. In the end we find a set of bias-corrected angular occupations ($\{\cos^2\tilde{\theta}_1, \cos^2\tilde{\theta}_2, \ldots, \cos^2\tilde{\theta}_N\}$) for which the sampled eigenvectors on average align with the naive estimates so that the ratio

$$\frac{\cos^2\theta_{i_{\mathrm{nv}}}}{\cos^2\theta'_i}$$

approaches one for all components. Similar to the eigenvalue bias-correction algorithm, the ξ exponent can be interpreted as a learning rate in log scale.

Bias correction of signal estimation: Following Kafashan et al. (17), the estimated signal difference follows

$$\delta\boldsymbol{\mu} = \delta\mathbf{f} + \boldsymbol{\eta}, \qquad \boldsymbol{\eta} \sim \mathcal{N}\left(0, \frac{2\boldsymbol{\Sigma}}{T}\right). \tag{S13.2}$$

We assume that the covariance matrix $\boldsymbol{\Sigma}$ is known, and let λ_i and $\mathbf{v}_i$ be the eigenvalues and eigenvectors of $\boldsymbol{\Sigma}$ (i.e., $\boldsymbol{\Sigma}\mathbf{v}_i = \lambda_i\mathbf{v}_i$). The naive estimate of the angular occupation is

$$\cos^2\tilde{\theta}_i = \frac{(\mathbf{v}_i^{\mathsf{T}}\delta\boldsymbol{\mu})^2}{|\delta\boldsymbol{\mu}|^2}. \tag{S13.3}$$

The noiseless cosine occupation is defined as

$$\cos^2\theta_i = \frac{(\mathbf{v}_i^{\mathsf{T}}\delta\mathbf{f})^2}{|\delta\mathbf{f}|^2}. \tag{S13.4}$$

A perturbative expansion in T^{-1} yields

$$E[\cos^2\tilde{\theta}_i] = \cos^2\theta_i\Big(1 - q\big(\operatorname{Tr}\boldsymbol{\Sigma} - 4(\bar{\lambda} - \lambda_i)\big)\Big) + q\lambda_i + O(T^{-2}), \tag{S13.5}$$

for $i = 1, \ldots, N$, with

$$q = \frac{2}{T|\delta\mathbf{f}|^2}. \tag{S13.6}$$

Throughout this note, big-O terms are bounded in magnitude by a constant multiple of the indicated order in the relevant limit. Here, $\bar{\lambda} = \sum_j \cos^2\theta_j \cdot \lambda_j$ is the weighted average of eigenvalues by the angular occupations. We provide the derivation below. From the equation above, neglecting terms of order $O(T^{-2})$ and replacing the expectation by the naive estimate, we obtain the bias-correction formula:

$$\cos^2\theta_i^{\mathrm{bc}} = \frac{\cos^2\tilde{\theta}_i - q\lambda_i}{1 - q\big(\operatorname{Tr}\boldsymbol{\Sigma} - 4(\bar{\lambda} - \lambda_i)\big)}. \tag{S13.7}$$

However, these are nonlinear equations with respect to $\cos^2\theta_i$ because $\bar{\lambda}$ is a function of a set of $\{\cos^2\theta_i\}$. Here, in practice, we obtain $\hat{\bar{\lambda}}$ by the plug-in estimate

$$\hat{\bar{\lambda}} = \sum_j \lambda_j \cos^2\tilde{\theta}_j. \tag{S13.8}$$

We apply a few iterations of the plug-in estimate by updating the angular occupation by the bias-corrected estimate. During this iteration, we clip the estimate at zero whenever the bias-correction returns a negative value. In the isotropic case, this bias-correction formula is equivalent, up to $O(T^{-1})$, to the one obtained under the factorization assumption described below (see the derivation at the end of this note). The bias correction with the term $\bar{\lambda} - \lambda_i$ explicitly captures the direction-dependent contribution of the noise variance: in the general anisotropic case it removes eigenmode-specific bias, while in the isotropic limit it reduces to a uniform rescaling determined by $\operatorname{Tr}\boldsymbol{\Sigma}$.

Focusing on the contribution of the term $+4q(\bar{\lambda} - \lambda_i)$ alone, which enters additively in the denominator, we note that it induces a differential rescaling of the estimated angular occupations.

In particular, eigenmodes with $\lambda_i < \bar{\lambda}$ increase the denominator and therefore suppress the bias-corrected angular occupation, whereas modes with $\lambda_i > \bar{\lambda}$ decrease the denominator and correspondingly increase the bias-corrected angular occupation.

Proof of the perturbative expansion

We assume that the noise $\boldsymbol{\eta} = \delta\boldsymbol{\mu} - \delta\mathbf{f}$ follows $\mathcal{N}(0, 2\boldsymbol{\Sigma}/T)$. Its projection onto $\mathbf{v}_i$ yields

$$\eta_i = \mathbf{v}_i^{\mathrm{T}}\boldsymbol{\eta} \sim \mathcal{N}(0, 2\mathbf{v}_i^{\mathrm{T}}\boldsymbol{\Sigma}\mathbf{v}_i/T) = \mathcal{N}(0, 2\lambda_i/T) \tag{S13.9}$$

From the definition of eigenvalues, the noise variance along $\mathbf{v}_i$ is proportional to λ_i. It also scales as T^{-1}. A perturbative expansion in T^{-1} follows from expanding the ratio in powers of the small noise $\boldsymbol{\eta}$, whose covariance scales as $\mathrm{Cov}(\boldsymbol{\eta}) = O(T^{-1})$.

Let $a_i = \mathbf{v}_i^{\mathrm{T}}\delta\mathbf{f}$ and note that $|\delta\mathbf{f}|^2 = \sum_j a_j^2$. Then $\mathbf{v}_i^{\mathrm{T}}\delta\boldsymbol{\mu} = a_i + \eta_i$, from which we obtain

$$\cos^2\tilde{\theta}_i = \frac{(a_i+\eta_i)^2}{\sum_j (a_j+\eta_j)^2} = \frac{a_i^2 + 2a_i\eta_i + \eta_i^2}{|\delta\mathbf{f}|^2 + 2\sum_j a_j\eta_j + \sum_j \eta_j^2}. \tag{S13.10}$$

We expand this ratio through $O(\|\boldsymbol{\eta}\|^2)$ as follows. Define

$$u = 2\sum_j a_j\eta_j, \qquad w = \sum_j \eta_j^2, \tag{S13.11}$$

so that the denominator is $|\delta\mathbf{f}|^2 + u + w$, where $u = O(\|\boldsymbol{\eta}\|)$ and $w = O(\|\boldsymbol{\eta}\|^2)$. Then

$$\frac{1}{|\delta\mathbf{f}|^2 + u + w} = \frac{1}{|\delta\mathbf{f}|^2}\,\frac{1}{1 + (u+w)/|\delta\mathbf{f}|^2}. \tag{S13.12}$$

Using $(1+z)^{-1} = 1 - z + z^2 + O(z^3)$ with $z = (u+w)/|\delta\mathbf{f}|^2$, we get

$$\frac{1}{|\delta\mathbf{f}|^2 + u + w} = \frac{1}{|\delta\mathbf{f}|^2}\left[1 - \frac{u+w}{|\delta\mathbf{f}|^2} + \frac{(u+w)^2}{|\delta\mathbf{f}|^4} + O(\|\boldsymbol{\eta}\|^3)\right]. \tag{S13.13}$$

Since $u = O(\|\boldsymbol{\eta}\|)$ and $w = O(\|\boldsymbol{\eta}\|^2)$, we have

$$(u+w)^2 = u^2 + 2uw + w^2 = u^2 + O(\|\boldsymbol{\eta}\|^3), \tag{S13.14}$$

hence, up to $O(\|\boldsymbol{\eta}\|^2)$,

$$\frac{1}{|\delta\mathbf{f}|^2 + u + w} = \frac{1}{|\delta\mathbf{f}|^2}\left[1 - \frac{u+w}{|\delta\mathbf{f}|^2} + \frac{u^2}{|\delta\mathbf{f}|^4}\right] + O(\|\boldsymbol{\eta}\|^3). \tag{S13.15}$$

Now multiply by the numerator $a_i^2 + 2a_i\eta_i + \eta_i^2$:

$$\begin{aligned}\cos^2\tilde{\theta}_i &= \left(a_i^2 + 2a_i\eta_i + \eta_i^2\right)\frac{1}{|\delta\mathbf{f}|^2}\left[1 - \frac{u+w}{|\delta\mathbf{f}|^2} + \frac{u^2}{|\delta\mathbf{f}|^4}\right] + O(\|\boldsymbol{\eta}\|^3)\\ &= \frac{a_i^2}{|\delta\mathbf{f}|^2} + \frac{2a_i\eta_i}{|\delta\mathbf{f}|^2} + \frac{\eta_i^2}{|\delta\mathbf{f}|^2} - \frac{a_i^2(u+w)}{|\delta\mathbf{f}|^4} - \frac{2a_i\eta_i\,u}{|\delta\mathbf{f}|^4} + \frac{a_i^2u^2}{|\delta\mathbf{f}|^6} + O(\|\boldsymbol{\eta}\|^3),\end{aligned} \tag{S13.16}$$

where we used that $(2a_i\eta_i)w = O(\|\boldsymbol{\eta}\|^3)$, $\eta_i^2(u+w) = O(\|\boldsymbol{\eta}\|^3)$, $2a_i\eta_i u^2 = O(\|\boldsymbol{\eta}\|^3)$, and $\eta_i^2u^2 = O(\|\boldsymbol{\eta}\|^4)$.

We now take expectations term by term. Since the noise vector $\boldsymbol{\eta}$ is mean zero, all terms linear in $\boldsymbol{\eta}$ vanish upon averaging. In particular,

$$E\left[\frac{2a_i\eta_i}{|\delta\mathbf{f}|^2}\right] = 0, \qquad E[u] = 0, \tag{S13.17}$$

where $u = 2\sum_j a_j\eta_j$.

The first nonvanishing contribution arises from the quadratic noise term in the numerator. Using $E[\eta_i^2] = 2\lambda_i/T$, we obtain

$$E\left[\frac{\eta_i^2}{|\delta\mathbf{f}|^2}\right] = \frac{2}{T|\delta\mathbf{f}|^2}\lambda_i. \tag{S13.18}$$

Next, consider the contribution from the expansion of the denominator proportional to $-a_i^2(u + w)/|\delta\mathbf{f}|^4$, where $w = \sum_j \eta_j^2$. Since $E[u] = 0$, only the w term survives, giving

$$E\left[-\frac{a_i^2}{|\delta\mathbf{f}|^4}(u+w)\right] = -\frac{a_i^2}{|\delta\mathbf{f}|^4}E[w] = -\frac{2}{T}\frac{a_i^2}{|\delta\mathbf{f}|^4}\operatorname{Tr}\boldsymbol{\Sigma}. \tag{S13.19}$$

Next, the mixed quadratic terms give nonzero contributions. Since

$$E[\eta_i u] = 2\sum_j a_j E[\eta_i\eta_j] = 2a_i E[\eta_i^2] = \frac{4a_i\lambda_i}{T}, \tag{S13.20}$$

we have

$$E\left[-\frac{2a_i\eta_i}{|\delta\mathbf{f}|^4}u\right] = -\frac{2a_i}{|\delta\mathbf{f}|^4}E[\eta_i u] = -\frac{8}{T}\frac{a_i^2}{|\delta\mathbf{f}|^4}\lambda_i. \tag{S13.21}$$

Moreover,

$$E[u^2] = 4E\left[\left(\sum_j a_j\eta_j\right)^2\right] = 4\sum_j a_j^2 E[\eta_j^2] = \frac{8}{T}\sum_j a_j^2\lambda_j, \tag{S13.22}$$

so

$$E\left[\frac{a_i^2}{|\delta\mathbf{f}|^6}u^2\right] = \frac{8}{T}\frac{a_i^2}{|\delta\mathbf{f}|^6}\,\delta\mathbf{f}^\top\boldsymbol{\Sigma}\,\delta\mathbf{f}. \tag{S13.23}$$

Collecting all terms, we obtain

$$E[\cos^2\tilde{\theta}_i] = \frac{a_i^2}{|\delta\mathbf{f}|^2} + \frac{2}{T|\delta\mathbf{f}|^2}\lambda_i - \frac{2}{T}\frac{a_i^2}{|\delta\mathbf{f}|^4}\operatorname{Tr}\boldsymbol{\Sigma} - \frac{8}{T}\frac{a_i^2}{|\delta\mathbf{f}|^4}\lambda_i + \frac{8}{T}\frac{a_i^2}{|\delta\mathbf{f}|^6}\,\delta\mathbf{f}^\top\boldsymbol{\Sigma}\,\delta\mathbf{f} + O(T^{-2}). \tag{S13.24}$$

We note that the noiseless angular occupation is

$$\cos^2\theta_i = \frac{a_i^2}{|\delta\mathbf{f}|^2}. \tag{S13.25}$$

Using

$$\bar{\lambda} = \frac{\delta\mathbf{f}^\top\boldsymbol{\Sigma}\,\delta\mathbf{f}}{|\delta\mathbf{f}|^2} = \frac{\sum_j a_j^2\lambda_j}{\sum_j a_j^2} = \sum_j \cos^2\theta_j\cdot\lambda_j, \tag{S13.26}$$

the expansion is written compactly as

$$E[\cos^2\tilde{\theta}_i] = \cos^2\theta_i + \frac{2}{T|\delta\mathbf{f}|^2}\Big(\lambda_i - \cos^2\theta_i\ \operatorname{Tr}\boldsymbol{\Sigma} + 4\cos^2\theta_i\cdot(\bar{\lambda}-\lambda_i)\Big) + O(T^{-2}). \tag{S13.27}$$

We define

$$q := \frac{2}{T|\delta\mathbf{f}|^2}. \tag{S13.28}$$

Neglecting terms of order $O(T^{-2})$, the expectation relation may be rewritten as

$$E[\cos^2\tilde{\theta}_i] = \cos^2\theta_i\Big(1 - q\big(\operatorname{Tr}\boldsymbol{\Sigma} - 4(\bar{\lambda}-\lambda_i)\big)\Big) + q\lambda_i. \tag{S13.29}$$

The equation above can be rewritten as

$$\cos^2\theta_i = \frac{E[\cos^2\tilde{\theta}_i] - q\lambda_i}{1 - q\big(\operatorname{Tr}\boldsymbol{\Sigma} - 4(\bar{\lambda}-\lambda_i)\big)}, \tag{S13.30}$$

for $i = 1,\ldots,N$. However, we note that these form nonlinear equations with respect to $\cos^2\theta_i$ because $\bar{\lambda}$ is a function of a set of $\{\cos^2\theta_i\}$.

Replacing the unknown expectation by the observed value $\cos^2\tilde{\theta}_i$ gives the bias-corrected plug-in estimator

$$\cos^2\theta_i^{\mathrm{bc}} = \frac{\cos^2\tilde{\theta}_i - q\lambda_i}{1 - q\big(\operatorname{Tr}\boldsymbol{\Sigma} - 4(\hat{\bar{\lambda}} - \lambda_i)\big)}, \tag{S13.31}$$

where $\hat{\bar{\lambda}}$ is obtained by the plug-in estimate

$$\hat{\bar{\lambda}} = \sum_j \lambda_j \cos^2\tilde{\theta}_j. \tag{S13.32}$$

In practice, we apply a few iterations of the plug-in estimate by updating the angular occupation by the bias-corrected estimate.

If $|\delta\mathbf{f}|^2$ is unknown, a consistent estimator at order T^{-1} is

$$|\delta\mathbf{f}|^2 \approx |\delta\boldsymbol{\mu}|^2 - \frac{2}{T}\operatorname{Tr}\boldsymbol{\Sigma}, \tag{S13.33}$$

which yields

$$q \approx \frac{2}{T\left(|\delta\boldsymbol{\mu}|^2 - \frac{2}{T}\operatorname{Tr}\boldsymbol{\Sigma}\right)}. \tag{S13.34}$$

Finally, if the noise is isotropic ($\lambda_i = \lambda$) and there is no preferred directionality in the covariance eigenbasis, then we expect $E[\cos^2\theta_i] = 1/N$, and we have $\bar{\lambda} = \lambda$. The bias correction equation is approximated by

$$\cos^2\theta_i^{\mathrm{bc}} \approx \frac{\cos^2\tilde{\theta}_i - q\lambda_i}{1 - q\operatorname{Tr}\boldsymbol{\Sigma}}. \tag{S13.35}$$

Since $q = O(T^{-1})$, we expand the denominator to first order:

$$\frac{1}{1 - q\operatorname{Tr}\boldsymbol{\Sigma}} = 1 + q\operatorname{Tr}\boldsymbol{\Sigma} + O(q^2). \tag{S13.36}$$

Substituting this expansion yields

$$\begin{aligned}\cos^2\theta_i^{\mathrm{bc}} &= \left(\cos^2\tilde{\theta}_i - q\,\lambda_i\right)\left(1 + q\operatorname{Tr}\boldsymbol{\Sigma}\right)\\ &= \cos^2\tilde{\theta}_i + q\operatorname{Tr}\boldsymbol{\Sigma}\cos^2\tilde{\theta}_i - q\,\lambda_i + O(q^2).\end{aligned} \tag{S13.37}$$

This isotropic expansion agrees, up to $O(T^{-1})$, with the factorization-based correction derived below. Recall that $\bar{\lambda} - \lambda_i$ captures the direction-dependent noise contribution and vanishes in the isotropic limit, leaving a uniform correction.

Bias-corrected angular occupations under factorization assumption: For comparison, we derive the simplified bias correction under the factorization assumption. Following Kafashan et al. (*17*), if the unbiased estimate of $\delta\boldsymbol{\mu}$ follows a normal distribution,

$$\delta\boldsymbol{\mu} \sim \mathcal{N}(\delta\mathbf{f}, 2\boldsymbol{\Sigma}/T), \tag{S13.38}$$

we have

$$\boldsymbol{\Sigma} = \frac{T}{2}\mathrm{E}\left[(\delta\boldsymbol{\mu} - \delta\mathbf{f})(\delta\boldsymbol{\mu} - \delta\mathbf{f})^{\mathrm{T}}\right]. \tag{S13.39}$$

Multiplying on the left by an eigenvector transpose $\mathbf{v}_i^{\mathrm{T}}$ of $\boldsymbol{\Sigma}$ and on the right by $\mathbf{v}_i$, we get

$$\begin{aligned}\mathbf{v}_i^{\mathrm{T}}\boldsymbol{\Sigma}\mathbf{v}_i &= \frac{T}{2}\mathbf{v}_i^{\mathrm{T}}\mathrm{E}\left[(\delta\boldsymbol{\mu} - \delta\mathbf{f})(\delta\boldsymbol{\mu} - \delta\mathbf{f})^{\mathrm{T}}\right]\mathbf{v}_i\\ \lambda_i &= \frac{T}{2}\mathbf{v}_i^{\mathrm{T}}\mathrm{E}[\delta\boldsymbol{\mu}\delta\boldsymbol{\mu}^{\mathrm{T}}]\mathbf{v}_i - \frac{T}{2}\mathbf{v}_i^{\mathrm{T}}\delta\mathbf{f}\delta\mathbf{f}^{\mathrm{T}}\mathbf{v}_i\\ &= \frac{T}{2}\mathrm{E}[\,|\delta\boldsymbol{\mu}|^2]\,\mathrm{E}[\cos^2\hat{\theta}_i] - \frac{T}{2}|\delta\mathbf{f}|^2\cos^2\theta_i.\end{aligned} \tag{S13.40}$$

Here, $\hat{\theta}_i$ denotes the random angle between the fixed true eigenvector $\mathbf{v}_i$ and the noisy estimate $\delta\boldsymbol{\mu}$. At the last equality, we assumed the factorization

$$\mathrm{E}[|\delta\boldsymbol{\mu}|^2 \cos^2\hat{\theta}_i] = \mathrm{E}[|\delta\boldsymbol{\mu}|^2]\mathrm{E}[\cos^2\hat{\theta}_i].$$

Summing over i, we get

$$\sum_i \lambda_i = \frac{T}{2}(\mathrm{E}[\,|\delta\boldsymbol{\mu}|^2] - |\delta\mathbf{f}|^2) \tag{S13.41}$$

$$\mathrm{E}[\,|\delta\boldsymbol{\mu}|^2] = \frac{2\sum_i \lambda_i}{T} + |\delta\mathbf{f}|^2. \tag{S13.42}$$

Substituting Eq. S13.42 into Eq. S13.40 and replacing $\mathrm{E}[\cos^2\hat{\theta}_i]$ with the observed naive estimate $\cos^2\tilde{\theta}_i$, we find the bias-corrected estimate of the cosine squares as

$$\cos^2\theta_i^{\mathrm{bc}} = \left(\frac{2\sum_{i'}\lambda_{i'}}{T|\delta\mathbf{f}|^2} + 1\right)\cos^2(\tilde{\theta}_i) - \frac{2\lambda_i}{T|\delta\mathbf{f}|^2}. \tag{S13.43}$$

In the isotropic case, the factorization holds through first order in q, and the resulting correction agrees with the general perturbative correction in Eq. S13.37 to that order.

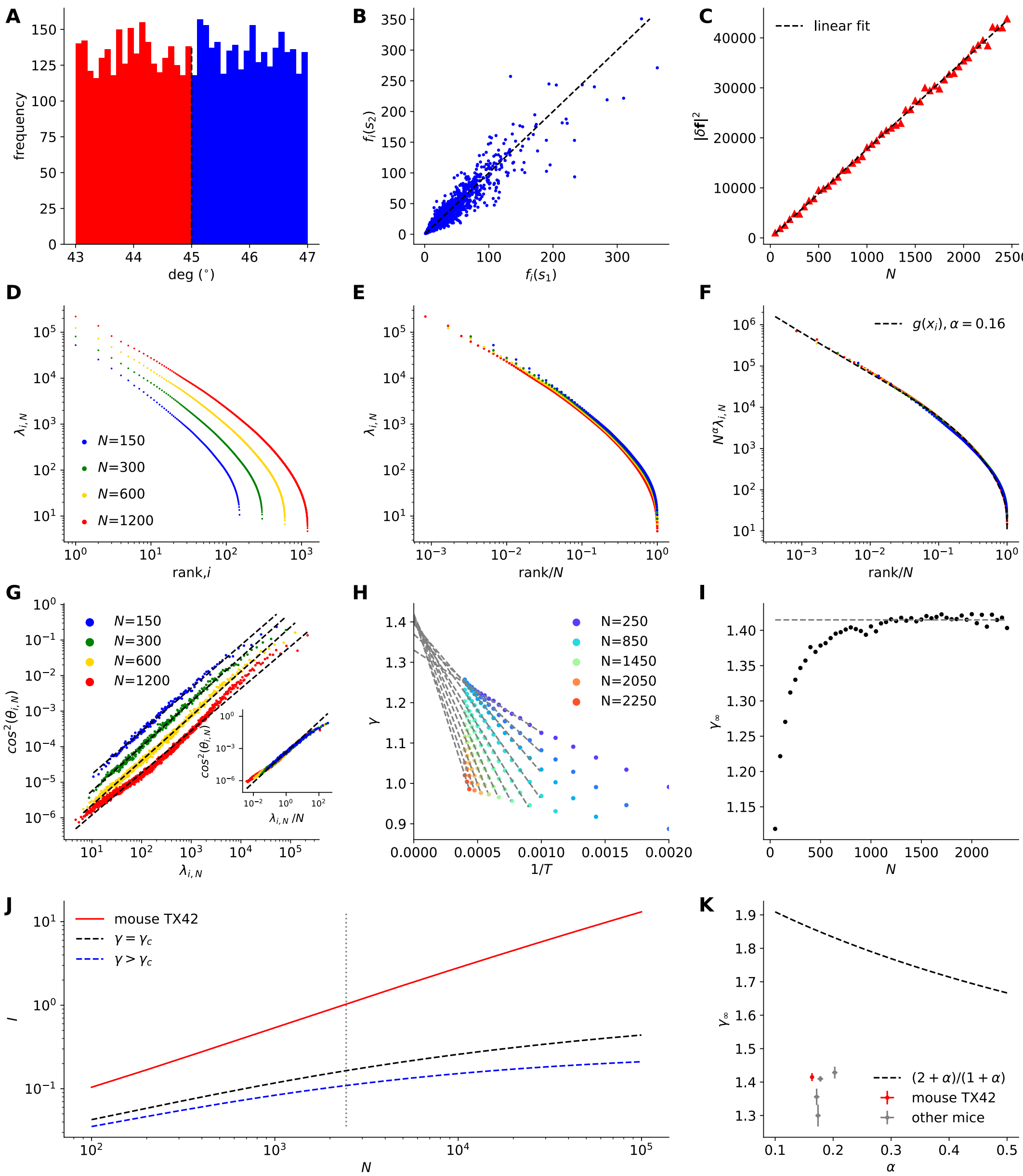

fig. S1. Analysis of mouse TX42. The figure styles follow Fig. 2 in the main text. The fitted scaling parameter is $\alpha = 0.164 \pm 0.002$. The fitted slope is $\gamma_\infty = 1.415 \pm 0.006$. **A**, A frequency histogram of grating stimulus orientations. **B**, Comparison of mean responses of neurons $f_i(s)$ to Stimuli 1 and 2. **C**, $|\delta \mathbf{f}_N|^2$ vs the subpopulation size N. **D**, Noise eigenspectra for different N. **E**, The eigenspectra vs the normalized rank, $(x_i =)i/N$. **F**, The re-scaled eigenspectra by multiplying N^α. The dashed line is the fitted $g(x_i)$. **G**, $\cos^2\theta_{i,N}$ vs $\lambda_{i,N}$ for different N. The dashed lines are linear fits. (Inset) $\cos^2\theta_{i,N}$ vs the scaled eigenvalue, $\lambda_{i,N}/N$. **H**, The fitted γ as a function of $1/T$ for different N. The solid lines are fitted linear functions. **I**, The intercept γ_∞ for different N. The average γ_∞ (dashed line) was 1.415 ± 0.006. **J**, The inferred LFI (solid line) as a function of N in the log-log plot. The dashed lines show theoretical LFI. **K**, The estimated γ_∞ vs α. The dashed line is a critical boundary.

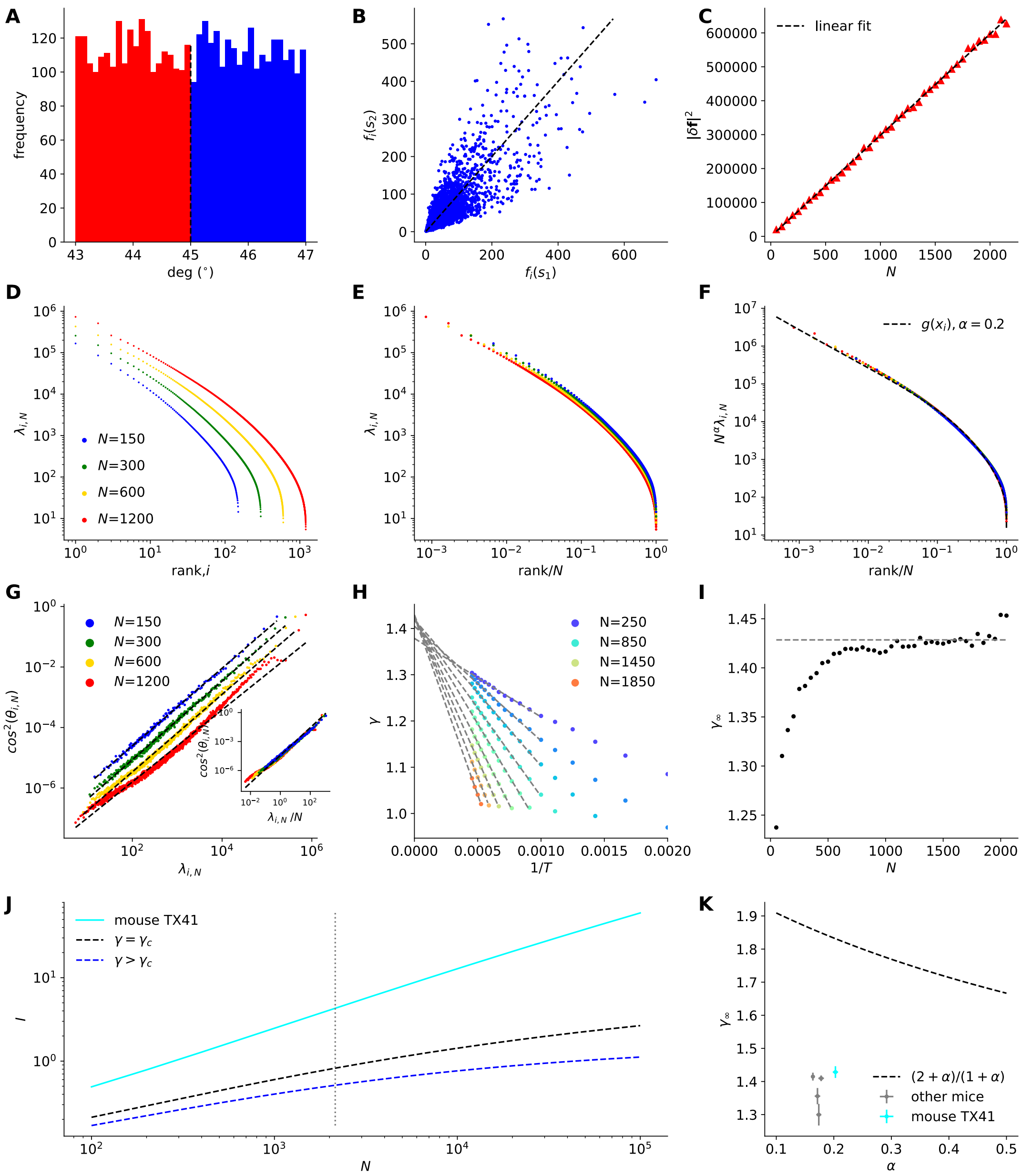

fig. S2. Analysis of mouse TX41. Same panels (A–K) as fig. S1. The fitted scaling parameter is $\alpha = 0.203 \pm 0.002$. The fitted slope is $\gamma_\infty = 1.428 \pm 0.009$.

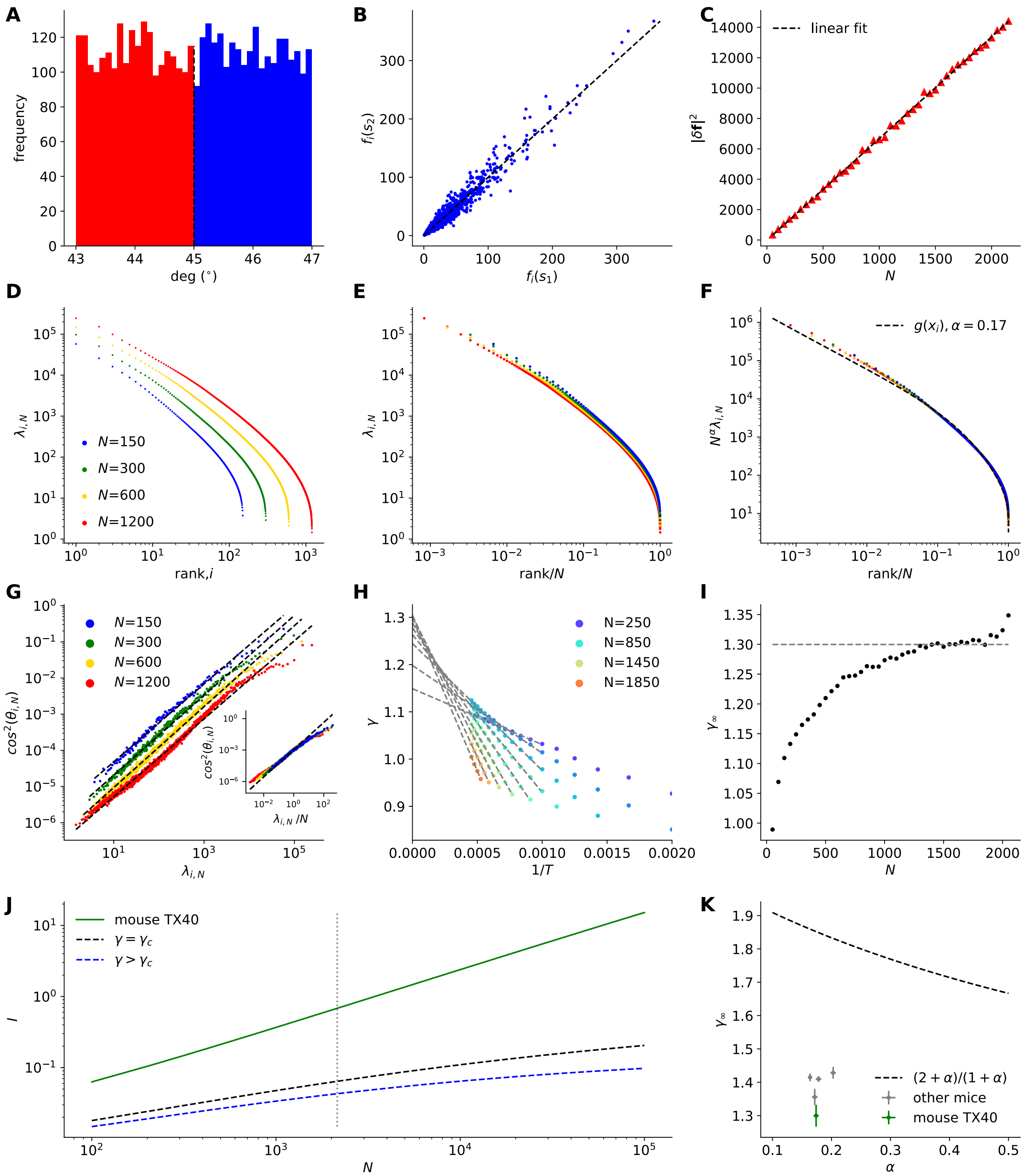

fig. S3. Analysis of mouse TX40. Same panels (A–K) as fig. S1. The fitted scaling parameter is $\alpha = 0.174 \pm 0.002$. The fitted slope is $\gamma_\infty = 1.3 \pm 0.016$.

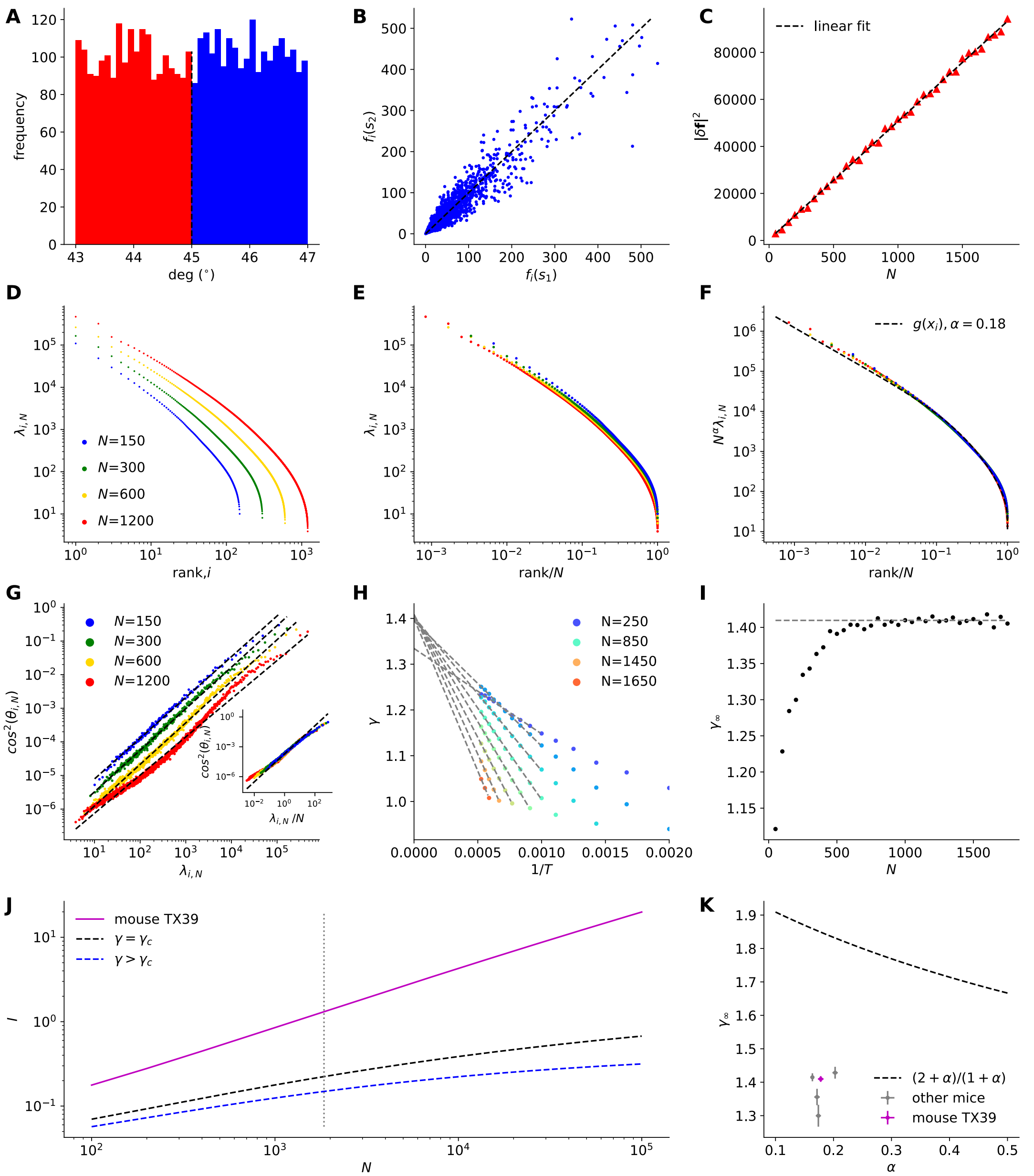

fig. S4. Analysis of mouse TX39. Same panels (A–K) as fig. S1. The fitted scaling parameter is $\alpha = 0.178 \pm 0.002$. The fitted slope is $\gamma_\infty = 1.41 \pm 0.004$.

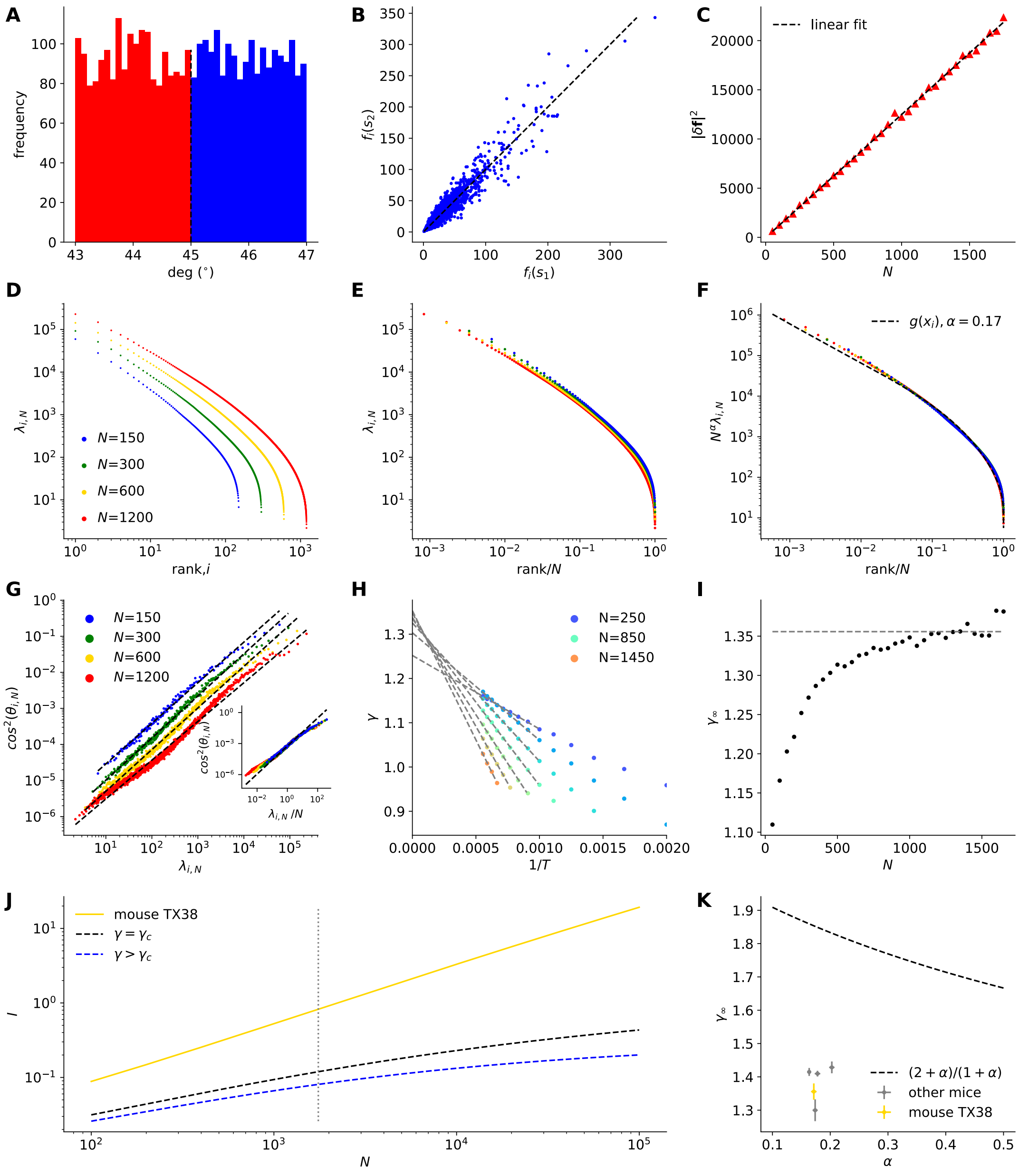

fig. S5. Analysis of mouse TX38. Same panels (A–K) as fig. S1. The fitted scaling parameter is $\alpha = 0.172 \pm 0.002$. The fitted slope is $\gamma_\infty = 1.356 \pm 0.012$.

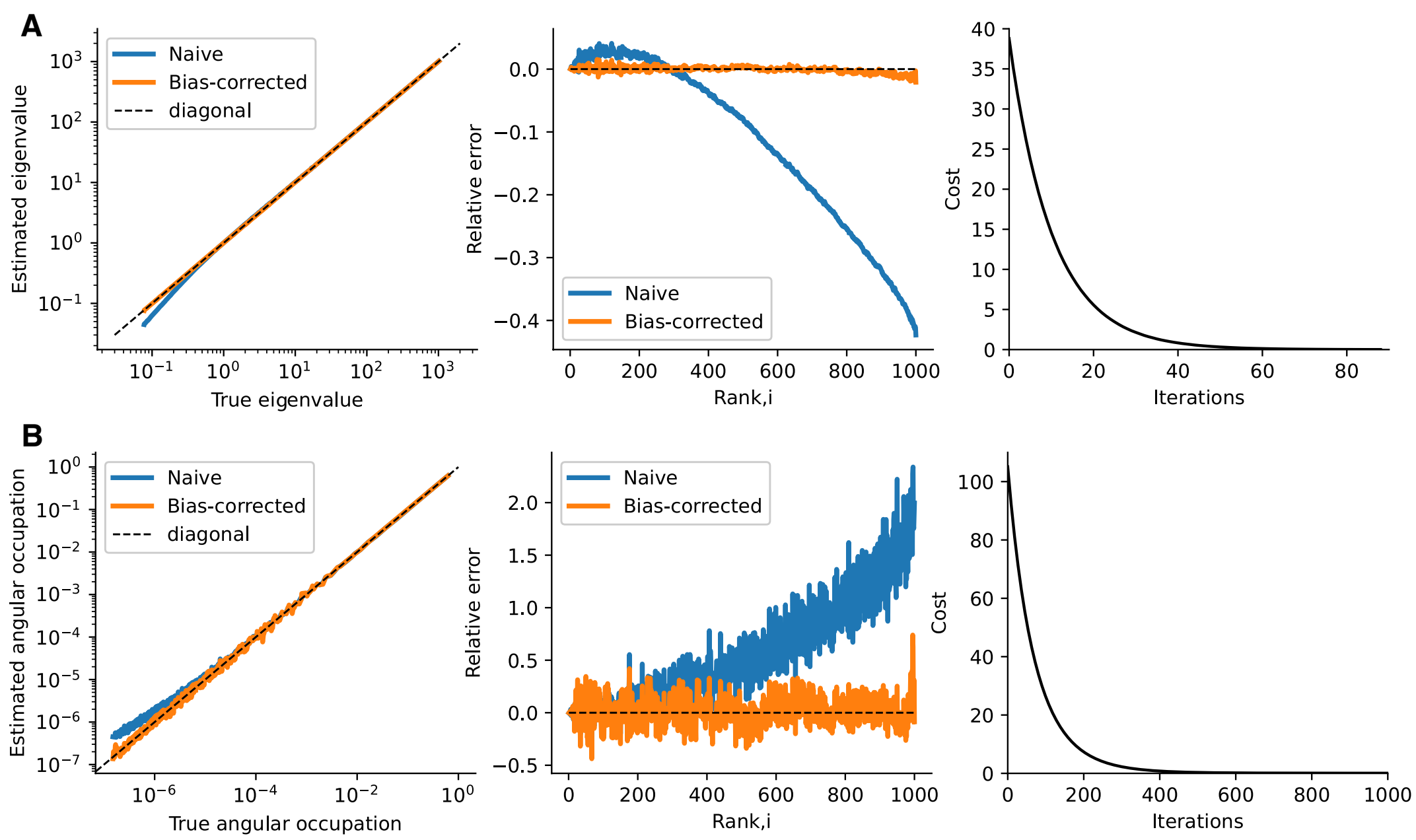

fig. S6. Validation of bias correction methods on simulated data. A, Covariance eigenvalue bias corrections. Left: Estimated versus true eigenvalues plotted on a log-log scale. The bias-corrected estimates fall on the diagonal, while the naive estimates deviate from it. Middle: To better visualize the error of the naive and bias-corrected estimates, the relative errors are shown versus the true eigenvalue ranks (lower ranks correspond to larger eigenvalues, $\lambda_1 > \lambda_2 > \cdots > \lambda_N$). Clearly, the naive estimates overstate the larger eigenvalues (yielding positive relative error) and understate the smaller eigenvalues (yielding negative relative error). The bias-corrected estimates show small errors. Right: The cost function of the algorithm $\sum_{i=1}^{N} \left[\log\left(\hat{\lambda}_i / \lambda'_i\right)\right]^2$ decreases to zero over iterations. **B**, The same as in **A**, but for the angular occupation corrections. The cost function plotted in the right panel is $\sum_{i=1}^{N} \left[\log\left(\cos^2\theta_{i,nv} / \cos^2\theta'_i\right)\right]^2$ for the angular occupation bias-correction algorithm. In this simulation, the subsampled population size is $N = 1000$, the number of subsampled populations is $m = 200$, the number of trials is $T = 2000$, $\alpha = 0.25$, and $\gamma = 1.6$.

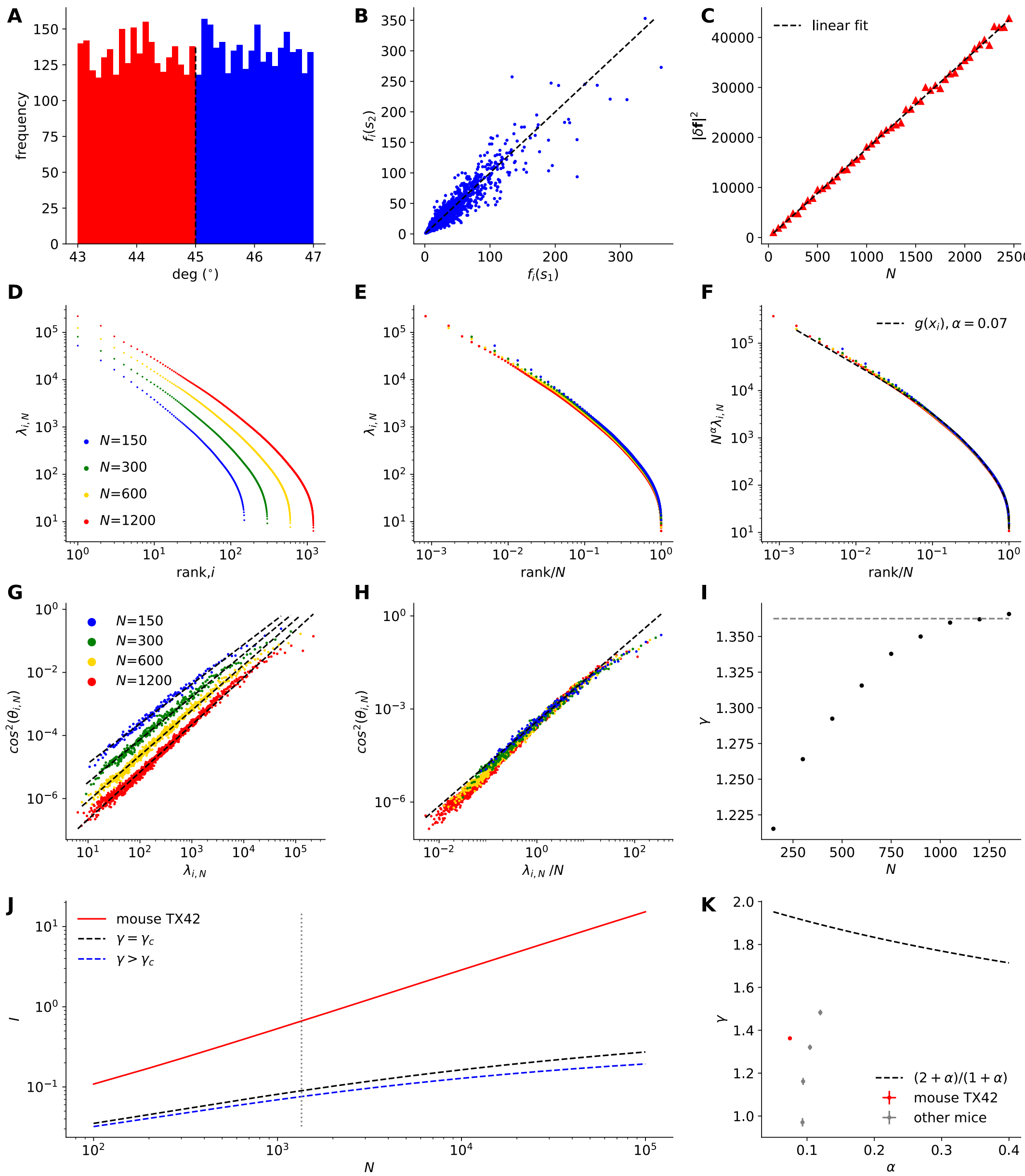

fig. S7. Bias-corrected analysis of mouse TX42 and the summary result from all mice. The figure styles follow Fig. 2 in the main text, except for **H** and **I**. The fitted scaling parameter is $\alpha = 0.074$. The fitted slope is $\gamma = 1.362 \pm 0.002$. **A**, A frequency histogram of grating stimulus orientations. **B**, Comparison of mean responses of neurons $f_i(s)$ to Stimuli 1 and 2. **C**, $|\delta \mathbf{f}_N|^2$ vs the subpopulation size N. **D**, Noise eigenspectra for different N. **E**, The eigenspectra vs the normalized rank, $(x_i =)i/N$. The fitted scaling parameter is $\alpha = 0.074$. The fitted parameters of $g(x_i)$ are $a = 2.118$, $b = 2.078$, $c = 2.407$, and $\nu = 0.213$. **F**, The re-scaled eigenspectra by multiplying N^α. The dashed line is the fitted $g(x_i)$. **G**, $\cos^2\theta_{i,N}$ vs $\lambda_{i,N}$ for different N. The dashed lines are linear fits. **H**, $\cos^2\theta_{i,N}$ vs the scaled eigenvalue, $\lambda_{i,N}/N$. The dashed line is a linear fit. **I**, The estimated γ for different N. The average γ for $N = 1000$–1350 (dashed line) was 1.362 ± 0.002. **J**, The inferred LFI (solid line) as a function of N in the log-log plot. The dashed lines show theoretical LFI. The vertical dashed line is the number of neurons used to estimate the parameters. **K**, The estimated γ vs α. The dashed line is a critical boundary.

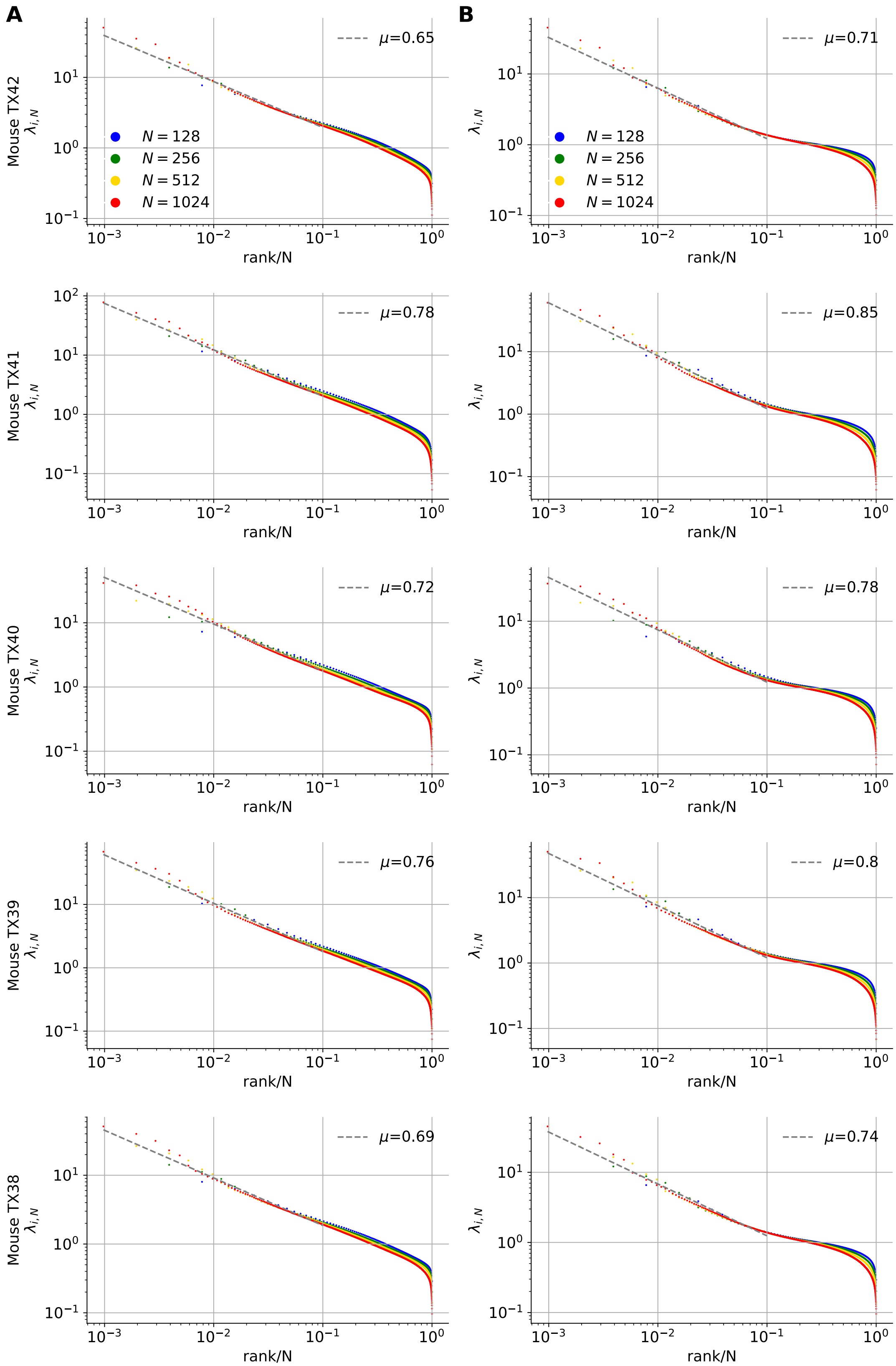

fig. S8. Eigenspectra of normalized covariance matrices for all mice. A, Eigenspectra of covariances of the activity normalized by nonzero mean for each neuron (Slope: $\mu = 0.72 \pm 0.05$ SD). The colors indicate $N = 128, 256, 512, 1024$. **B**, Eigenspectra of correlation coefficient matrices (Slope: $\mu = 0.78 \pm 0.05$ SD). The bias-correction methods were applied to this analysis.

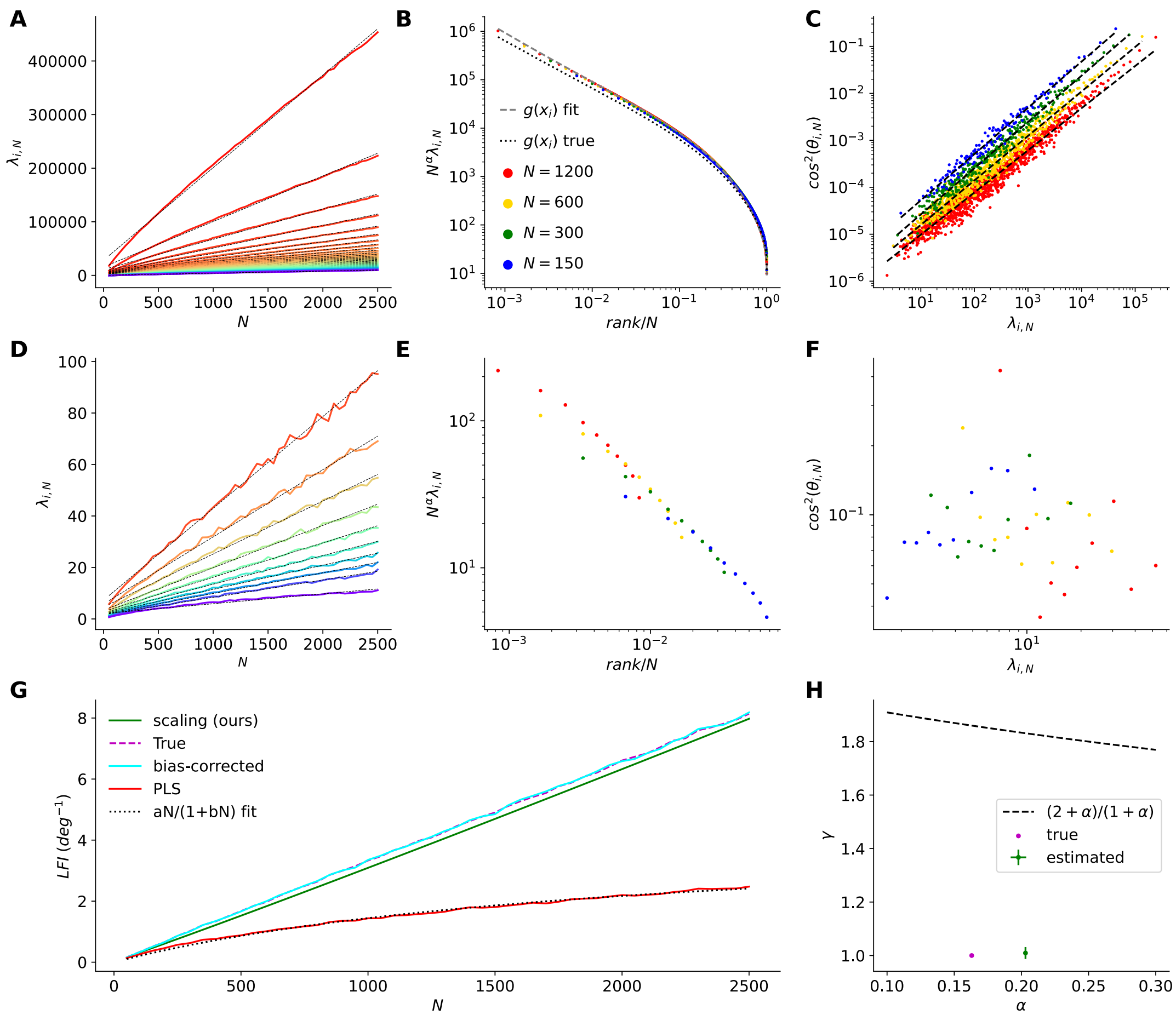

fig. S9. Comparison of the PLS analysis with the proposed scaling-based method using simulated data with nonzero α. The simulated data were generated from the eigenspectrum fitted to mouse TX42 ($\alpha = 0.163$). Angular occupations were obtained using Eq. 7 with $\gamma = 1$. **A**, Eigenvalues as a function of population size N. Only the largest 50 eigenvalues are shown. The leading eigenvalues scale linearly for $\alpha > 0$. Black lines, linear fits. **B**, Collapsed eigenspectrum of the simulated data (colored dots) with the true (dotted) and fitted (dashed) eigenspectra. The fitted scaling parameter is $\alpha = 0.203 \pm 0.0001$. **C**, Relation between the sampled eigenvalues and angular occupations. The estimated γ by the extrapolation method (see main text) is $\gamma = 1.009 \pm 0.011$. **D**, Eigenvalues of the 10-dimensional subspace obtained by the PLS method as a function of population size N. Black lines, linear fits. **E**, Collapsed eigenspectrum of the subspaces obtained by the PLS method. **F**, Relation between the sampled eigenvalues and angular occupations in the subspaces obtained by the PLS method. **G**, Comparison of the LFI estimated by the scaling-based method (green) and PLS method (red) with the true LFI (dashed magenta). The PLS estimate underestimates the true LFI and is well fitted by the bounded function $aN/(1 + bN)$ (dotted black), while the scaling-based method correctly estimates the true unbounded LFI. For comparison, the bias-corrected estimate of the LFI is shown (cyan). **H**, Estimated γ_{∞} vs α by the scaling-based method. The dotted line is a critical boundary (Eq. 8), below which the LFI is unbounded. Error bars, 2 SD.

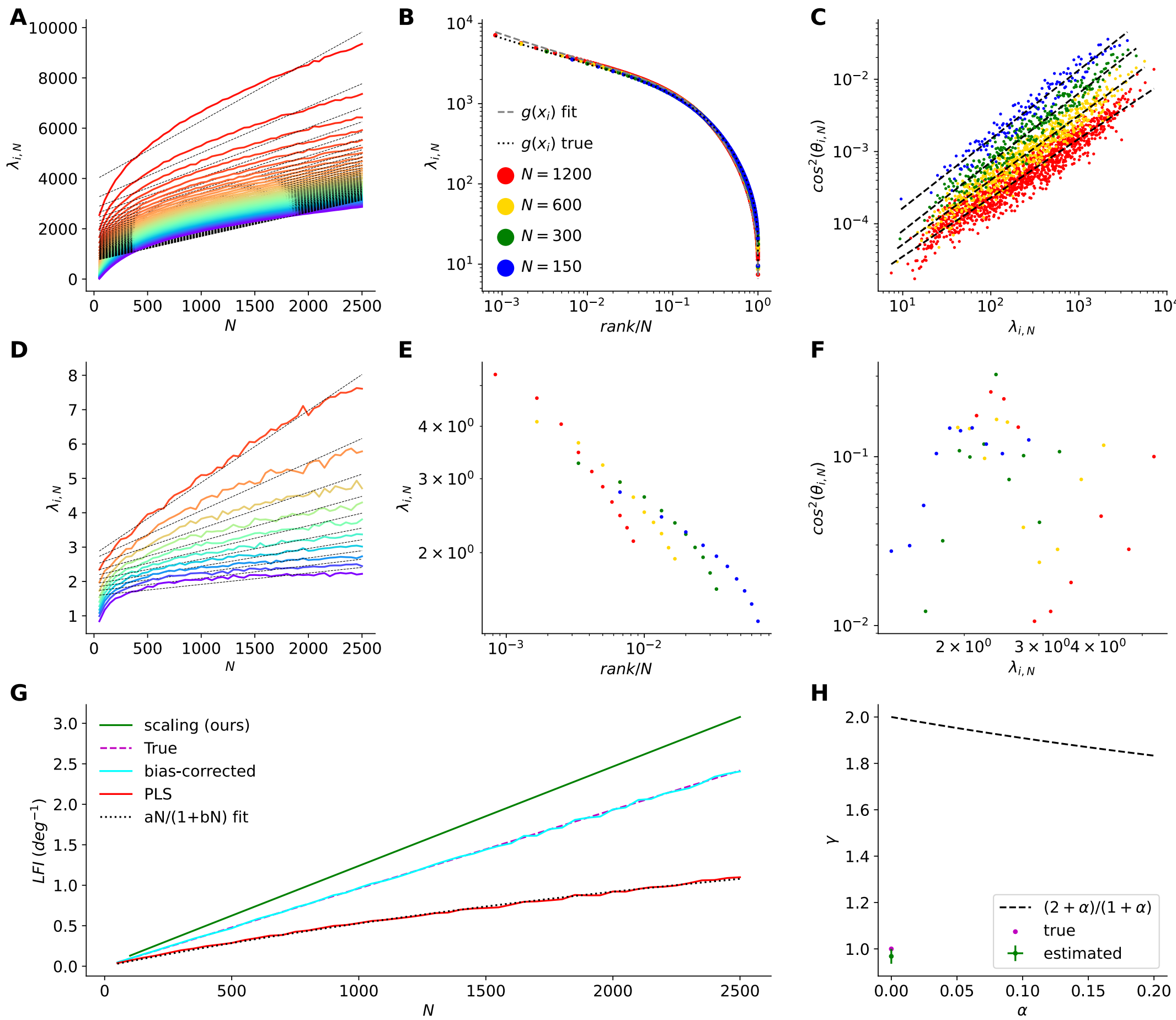

fig. S10. Comparison of the PLS analysis with the proposed scaling-based method using simulated data with $\alpha = 0$. The analysis is the same as in fig. S9, but with simulated data generated using $\alpha = 0$ and $g(x)$, whose parameters were those fitted to mouse TX42 except for the power-law exponent, which was set to 0.5. Angular occupations were obtained using Eq. 7 with $\gamma = 1$. **A**, Eigenvalues as a function of population size N. Only the largest 50 eigenvalues are shown. All eigenvalues scale sublinearly for $\alpha = 0$. Black lines, linear fits. **B**, Eigenspectrum of the simulated data (colored dots) with the true (dotted) and fitted (dashed) eigenspectra. The scaling-collapse fit is consistent with $\alpha = 0$. **C**, Relation between the sampled eigenvalues and angular occupations. The estimated γ by the extrapolation method (see main text) is $\gamma = 0.968 \pm 0.016$. **D**, Eigenvalues of the 10-dimensional subspace obtained by the PLS method as a function of population size N. One of the subspace eigenvalues scales linearly. Black lines, linear fits. **E**, Collapsed eigenspectrum of the subspaces obtained by the PLS method. **F**, Relation between the sampled eigenvalues and angular occupations in the subspaces obtained by the PLS method. **G**, Comparison of the LFI estimated by the scaling-based method (green) and PLS method (red) with the true LFI (dashed magenta). The PLS estimate underestimates the true LFI and is well fitted by the bounded function $aN/(1 + bN)$ (dotted black), while the scaling-based method overestimates the true unbounded LFI. For comparison, the bias-corrected estimate of the LFI is shown (cyan). **H**, Estimated γ_∞ vs α by the scaling-based method. The dotted line is a critical boundary (Eq. 8), below which the LFI is unbounded. Error bars, 2 SD.
